# A snapshot of gut microbiota of an adult urban population from Western region of India

**DOI:** 10.1371/journal.pone.0195643

**Published:** 2018-04-06

**Authors:** Disha Tandon, Mohammed Monzoorul Haque, Saravanan R., Shafiq Shaikh, Sriram P., Ashok Kumar Dubey, Sharmila S. Mande

**Affiliations:** 1 Bio-Sciences R&D Division, TCS Research, Tata Consultancy Services Ltd., Hadapsar Industrial Estate, Pune Maharashtra, India; 2 Tata Chemicals Ltd. Innovation Centre, Ambedveth, Mulshi, Pune, Maharashtra, India; 3 Veeda Clinical Research Pvt. Ltd. Near IIM, Ambawadi, Ahmedabad, Gujarat, India; 4 Genotypic Technology (P) Ltd., Poojari Layout, Bangalore, India; Babasaheb Bhimrao Ambedkar University, INDIA

## Abstract

The human gut microbiome contributes to a broad range of biochemical and metabolic functions that directly or indirectly affect human physiology. Several recent studies have indicated that factors like age, geographical location, genetic makeup, and individual health status significantly influence the diversity, stability, and resilience of the gut microbiome. Of the mentioned factors, geographical location (and related dietary/socio-economic context) appears to explain a significant portion of microbiome variation observed in various previously conducted base-line studies on human gut microbiome. Given this context, we have undertaken a microbiome study with the objective of cataloguing the taxonomic diversity of gut microbiomes sampled from an urban cohort from Ahmedabad city in Western India. Computational analysis of microbiome sequence data corresponding to 160 stool samples (collected from 80 healthy individuals at two time-points, 60 days apart) has indicated a *Prevotella*-dominated microbial community. Given that the typical diet of participants included carbohydrate and fibre-rich components (predominantly whole grains and legume-based preparations), results appear to validate the proposed correlation between diet/geography and microbiome composition. Comparative analysis of obtained gut microbiome profiles with previously published microbiome profiles from US, China, Finland, and Japan additionally reveals a distinct taxonomic and (inferred) functional niche for the sampled microbiomes.

## Introduction

Recent advances in healthcare and medical research suggest that human beings are actually a composite organism, and most of the genetic information contained on/ within a living human body can be attributed to the billions of microbes residing on/ within it [1]. Consequently the health of any individual is dependent (at least to a certain extent) on the dynamics of various microbial communities (referred to as microbiomes) inhabiting a human body. Technological developments in the field of metagenomics have further revolutionized our understanding of the human microbiome in health and disease. For example, an aberrant or deviant gut microbiome (one of the most studied microbiomes) has been implicated in several diseases and physiological disorders like enteric infections [2,3], obesity [4,5], Crohn’s disease [6], Type II diabetes [4,5], colorectal cancer[7,8], etc. Interestingly, even nutritional and neurological disorders such as malnutrition[9,10], Parkinson's disease[11,12], schizophrenia[13], etc. are indicated to have a strong microbial basis. Study of different microbiomes, therefore, holds the promise of complementing our existing knowledge of human physiology, and paves the way for the development of new therapeutic approaches. Understanding the structure and function of microbiomes sampled from multiple subject cohorts is expected to provide important insights into the healthy commensal microflora, dysbiotic patterns related to onset of different disease/ disorders, disease susceptibility, impact of various types of co-infections, and on an optimistic note—in discovering (and/ or defining) alternate, novel, and improved diagnostic methods as well as therapeutic regimes[14].

Factors like age, sex, dietary profile, genetic make-up (ethnicity), geographic location, disease status, etc. are known to govern the structure, function, and succession pattern of various microbial communities present on/ within an individual [15–17]. Significant structural and functional variations are therefore expected to exist between microbiomes obtained from individuals belonging to different geographical regions [15]. Consequently, prior to analysing microbiomes sampled from healthy and diseased states, it becomes imperative to understand the structure and function of microbial communities sampled from healthy cohorts from different geographies. Several individual studies as well as global scale-initiatives have been reported on the gut microbial communities sampled from individuals in developed economies[15–18]. In this context, it may be noted that, except for a previous study[19] with a small cohort size of 34, a comprehensive study from an Indian perspective is lacking.

The present study aims at filling this gap by performing a gut microbiome study with a cohort of 80 urbansubjects from Ahmedabad city in Western India (hereafter referred to as ‘Western India’ cohort). Besides helping in cataloguing the repertoire of gut microbes in Western Indian population, it is anticipated that the base-line data obtained in this study will enable in deciphering critical insights with respect to the correlation of the structure of the gut microbiome and various diseases/ metabolic disorders from a regional perspective. The cohort size (n = 80) taken in this study is reasonably large enough to facilitate comparison with datasets from other geographies, and for deciphering distinctive structural and functional aspects of the Western Indian Gut Microbiome. It may be noted that earlier studies [16,20] pertaining to region specific microbial community characteristics had more or less equivalent cohort sizes.

## Methods

### Subject recruitment and selection criteria

This study was conducted at Veeda Clinical Research, Ahmedabad, India. The protocol was approved by the Anveshhan Independent Ethics Committee (Ahmedabad, Gujarat, India) associated with the study centre. The study was conducted as per the pertinent requirements of the ICMR guidelines for Biomedical Research on Human Subjects, Good Clinical Practices for Clinical Research in India. The protocol was carried out in accordance with the approved guidelines, and was in agreement with Declaration of Helsinki principles. Prior to the start of the study, healthy, willing, volunteers from Ahmedabad city in Gujarat were individually provided with a subject informed consent document that provided complete information on the sampling procedure and the objectives of the study. Subjects reported to clinical facility for 1 and 60 days and provided ambulatory stool samples. All subjects were advised to consume a regular diet during the course of study. Screening data pertaining to enrolled subjects was captured on a paper format, and then collated in an excel sheet and verified.

### Demography and other baseline characteristics

A total of 80 subjects were enrolled in the study. The subjects were mainly from lower socio-economic background from Western part of India (Ahmedabad). Metadata information pertaining to various subjects is provided in S1 Table. It may be noted that the dietary habits of all 80 participants were more or less in line with local dietary habits and primarily comprised of fruits, vegetables, wheat, millet, sorghum, dairy products, sprouts, leafy vegetables, rice and pulses. Consumption of meat and fish is relatively lower as compared to other regions in India[21,22].

### Inclusion and exclusion criteria

During subject selection, the following inclusion criteria were considered:

1. Subjects aged from 18 to 55 years.
2. Subjects' weight within normal range according to normal values for Body Mass Index (18.50 to 30.00 kg/m2) with minimum of 45 kg weight.
3. Subjects with normal health as determined by personal medical history and physical examination
4. Subjects having negative alcohol breath test.
5. Subjects willing to adhere to the protocol requirements and to provide written informed consent.
6. Female subjects having negative urine pregnancy test at screening and on admission day 01 of study.

The exclusion criteria included:

1. History or presence of heavy smoking and alcohol or drug abuse.
2. History or presence of asthma, urticaria, gastric and duodenal ulceration, thyroid dysfunction, adrenal dysfunction, organic intracranial lesion and cancer.
3. Usage of any medication during last one month or OTC medication during last two weeks prior to day 01 of the study.
4. Major illness within past 3 months.
5. Consumption of grapefruit juice, xanthine-containing products, tobacco containing products or alcohol within 48 hours prior to day 01 of study.
6. Subjects who have been on an irregular or abnormal diet during the four weeks preceding the study.
7. Female subjects who are currently breast feeding.

### Sample collection and library preparation

Stool samples were collected preferably in the morning of scheduled visit at Veeda Clinical Research Pvt. Ltd. All subjects were instructed to visit the clinical facility for providing samples. Stool samples were collected in pre-labelled OMNIgene Gut (OMR 200) tubes procured from DNA Genotek, Canada. All the preparations and collection steps were followed as mentioned in the kit protocol. Transfer of the stool samples to the DNA extraction and sequencing facility was done as per recommendations and necessary precautions defined in protocol.

DNA from the collected sample was extracted using Qiagen DNeasy Blood & Tissue Kit. Nucleic acid concentration and purity was estimated using spectrophotometry. For library preparation, 4 ng of nanodrop quantified DNA was used for amplifying V3-V4 region of 16S gene using TAKARA ExTaq polymerase with specific primers which also have a 'tag' sequence that were complementary to Illumina sequence adapter and index primers from the Nextera XT index kit V2. This round of PCR generated single amplicon of ~500–550 bp. The amplified products were checked on the agarose gel and before proceeding for indexing PCR. In the next round of PCR (Indexing PCR), Illumina sequencing adapters and dual indexing barcodes were added to 25 ng (by nanodrop) of amplified product using limited cycle PCR to give a final product of ~600–650 bp. All the libraries after second round of PCR were normalized, quantified, estimated and validated for quality by running an aliquot on High Sensitivity Tape Station-Agilent prior to sequencing on Illumina MiSeq (Illumina, San Diego, USA). The V3-V4 primer and the adapter details are as mentioned below.

V3-V4 amplification primers

Read1:

5’-TCGTCGGCAGCGTCAGATGTGTATAAGAGACAGCCTACGGGNGGCWGCAG-3’

Read2:

5’-GTCTCGTGGGCTCGGAGATGTGTATAAGAGACAGGACTACHVGGGTATCTAATCC-3’

V3-V4 (Using Nextera XT Barcode Kit)

Index 2 Read

5’-AATGATACGGCGACCACCGAGATCTACAC[i5]TCGTCGGCAGCGTC-3’

Index 1 Read

5’-CAAGCAGAAGACGGCATACGAGAT[i7]GTCTCGTGGGCTCGG-3’

‘W’ represents a degenerate base.

### Pre-processing and taxonomic profiling

Raw (paired-end) sequence data (in fastq format) was initially pre-processed to remove sequences of low quality (minimum mean phred score less than 25) and insufficient length (less than 100bp). The resulting fasta files were then provided as input to the V-Xtractor 2.0 program[23] for retaining the V3-V4 specific region in each sequence. Taxonomic profiles (S2 Table) corresponding to each sample were obtained using the Ribosomal Database Project (RDP) classifier, version 2.11[24] executed using a bootstrap threshold of 80%. Rarefaction plot was generated using PAST software[25].

### Core taxa identification

Core taxa were identified using the following approach. 80% of the samples constituting the dataset were drawn randomly. The taxon which has the highest Median Abundance (MA) in the drawn subset was identified. Taxa with abundance value at least 80% of the identified MA value in the previous step were retained as core taxa for that subset. The entire process was iterated around 1000 times. Taxa that were identified in at-least 80% of iterations were designated as ‘core’ for the respective dataset. The rigorous boot-strapped procedure stated above was adopted to ensure stringency while calling a given taxon as ‘core’ for a given dataset.

### Computation of alpha diversity measures and functional potential

Shannon, Simpson and Chao were computed using functions implemented in the R-vegan package. Functional profiles at pathway and pathway-class levels (at various PEC levels) were obtained using Vikodak's Global Mapper Module[26].

### Differential ‘genera/pathways/pathway classes’ analysis using bootstrapped approach

Genera/ pathways/ pathway-classes with significantly different relative abundance between the three clusters were identified using the following bootstrapped approach–

Step 1: 25 samples (i.e. 75% of the total number of samples from the smallest cluster) were randomly chosen from each cluster (i.e. 1a, 1b, and 2).
Step 2: In each cluster, the median abundances of constituent genera/ pathways/ pathway classes in each randomly chosen subset of samples were computed.

The above steps were repeated 100 times to obtain 100 values of median abundances for each genera/ pathway/ pathway class in each cluster. Kruskal-Wallis rank sum test (as implemented in 'pgirmess' R package) was then carried out between the obtained median abundance values (for each genera/ pathway/ pathway class) in each of the clusters classes, and genera/ pathways/ pathway classes with significantly different abundance were identified using Benjamini-Hochberg (BH) p-value correction at an FDR of 0.0001. The entire procedure described above was bootstrapped 100 times. Genera/ pathways/ pathway classes which were observed as having a significantly different abundance (post BH correction) in at least 99% of iterations were retained.

## Results

To assess core taxonomic characteristics of gut microbiomes(sampled in the present study), taxonomic profiles of 160 samples (comprised of approximately 25 million high-quality sequences) taken from 80 (apparently healthy) Western Indian individuals at two time points 60 days apart (hereafter referred to as WIGM-T1 and WIGM-T60) were obtained. Rarefaction plot (S1 Fig) indicated that, for most samples the curves did level off. Only for a small subset of samples, they did not reach an ideal asymptote. However, Good’s coverage of >97% obtained for all samples, indicates adequate sequencing coverage.

### Community composition in the gut microbiomes of Western Indian subjects

**Fig 1:**
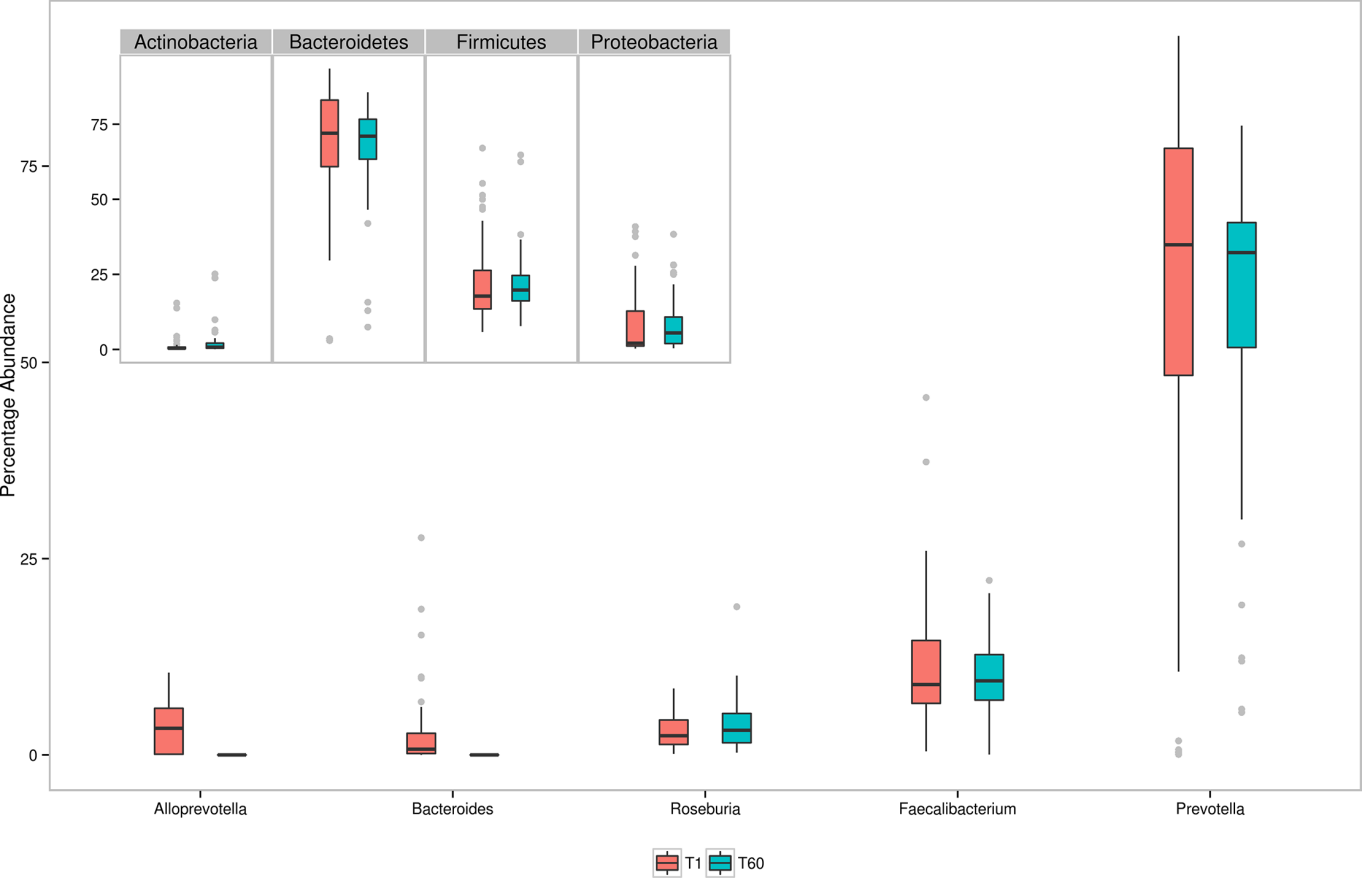
Box plots representing relative abundances of five most abundant bacterial genera at the two time points, i.e. IGM-T1 and IGM-T60. Inset indicates corresponding box plots at phylum level.

Analysis of taxonomic profiles indicates that the Western Indian Gut Microbiome is dominated by microbes belonging to four phyla, viz. *Bacteroidetes* (median abundance 71.5%), *Firmicutes* (18.7%), *Proteobacteria* (3.8%), and *Actinobacteria* (0.6%). In other words, approximately 95% of sequences are observed to be assigned to microbes belonging to these four phyla (the remaining 5% being assigned to 16 other phyla in miniscule proportions). At the genus level, 78 unique genera (90% of them common to both time points) are observed to have non-zero median abundance. Greater than 80% of the sequences are assigned to 5 genera viz. *Prevotella*, *Faecalibacterium*, *Alloprevotella*, *Roseburia*, and *Bacteroides*. Fig 1 depicts the abundance pattern of major genera and their corresponding phyla (as inset) in the Western Indian population. In summary, the taxonomic profiles corresponding to the Western Indian gut microbiome indicate a *Prevotella*-dominated community that closely resembles (a) the profiles obtained in an earlier Indian microbiome study(19), and (b) the taxonomic composition of gut microbiome samples clustered as 'Enterotype II' (as described in Arumugam et al., 2011[27]). Dietary habits prevalent in the Indian subcontinent (and particularly that prevalent in Ahmedabad, the geographic location of the recruited 80 participants) lend credence to this result. The typical diet of participants in the present study included simple and complex carbohydrates (i.e. rice, wheat, sorghum and millet) and fibre-rich components (i.e. fruits, vegetables, whole grains, sprouts, etc.)[21,22]. It may further be noted that previous studies [28,29] had also indicated gut microbiota to be enriched with *Prevotella* in individuals who consumed fibre-rich foods (as was the typical diet of individuals of the present study).

The two phyla viz. *Proteobacteria* and *Actinobacteria* are observed to be relatively minor constituents (as compared to the phyla *Bacteroidetes* and *Firmicutes*), therefore reflecting a balanced gut-associated microbial community [30] in the recruited participants. *Sutterella* and *Succinivibrio* are major genera that were found to be dominant among genera belonging to phylum *Proteobacteria*. Although *Sutterella* has been associated with human diseases such as IBD and Autism [31,32], recent studies have indicated that this intestinal wall adhering bacterium plays an important immune-modulatory role in the human gastrointestinal tract [33]. On the other hand, the fibre-degrading potential of *Succinivibrio* has also been well documented. In general, abundance of members belonging to phylum *Proteobacteria* has been found to be positively correlated to the amount of fibre intake. Notwithstanding this, the relatively lower abundance of members belonging to phylum *Proteobacteria* (as compared to the major phyla *Bacteroidtes* and *Firmicutes*) indicates an (apparently) favourable state of gut health, and likely absence of sustained epithelial dysfunction [34] in the recruited cohort.

Analysis further indicates the presence of phylum *Actinobacteria*, a few members of which are known for their beneficial probiotic effects. In particular, members of genus *Bifidobacterium* are observed to be present in the analysed taxonomic profiles, albeit in minor proportions. Species belonging to *Bifidobacterium* are especially known for their role in the breakdown of metabolic by-products which are generated as a result of partial digestion of complex dietary carbohydrates [35].

### Intra-community genera correlations in the gut microbiomes of Western India subjects

**Fig 2:**
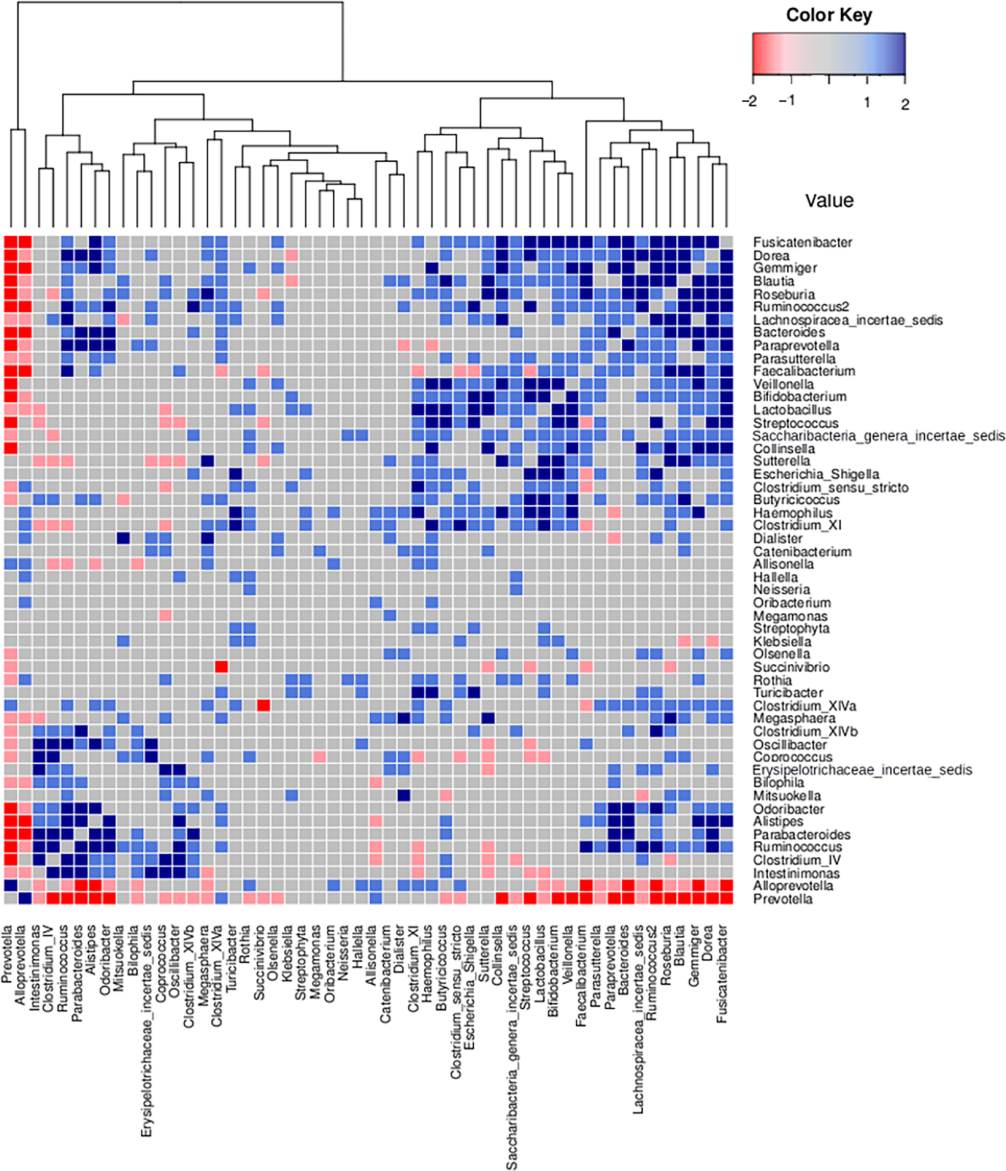
Heat map representing Pearson correlation values between various genera pairs. Positive or negative correlations (between a given pair of genera) identified at both time points (T1 and T60) are indicated as dark blue and dark red respectively. Interactions that are found at only 1 time point are depicted in respective lighter shades.

In order to investigate community-level relationships between various genera constituting the Western Indian Gut microbiome, correlation analysis was performed for identifying genera-pairs having significant positive or negative correlations (Benjamini-Hochberg corrected p-value < 0.1) between their patterns of abundance. The procedure was independently performed on WIGM-T1 and WIGM-T60. Results of these experiments (graphically depicted in Fig 2) indicate a negative correlation between *Prevotella* and (a significant subset of) other bacterial species constituting the microbial community. A cluster of bacterial species comprising of *Dorea*, *Blautia*, *Roseburia*, *Ruminococcus*, *Bacteroides*, *Paraprevotella*, *Faecalibacterium*, and *Collinsella* are observed to have positive correlation between their patterns of abundances. Given that these correlations are observed at both time-points, the confidence attributed to these (computed) pair-wise correlations (between the stated genera) can be considered to be on the higher side. It is important to note that these bacterial genera are known players in fermentation of complex carbohydrates (obtained through breakdown of dietary fibre), and significantly involve, as metabolic substrates/ by-products, short chain fatty acids (butyrate, proprionate, etc.) which are known to boost the immune system through induction of regulatory T-cells in the gut[36,37]. It may be noted that short chain fatty acids and hydrogen, typical end-products of metabolism of complex carbohydrates, have well-established clinical benefits[38,39].

### Comparison of taxonomic profiles generated fromgut microbiomes of Western Indian subjects and those corresponding toother geographies

**Fig 3:**
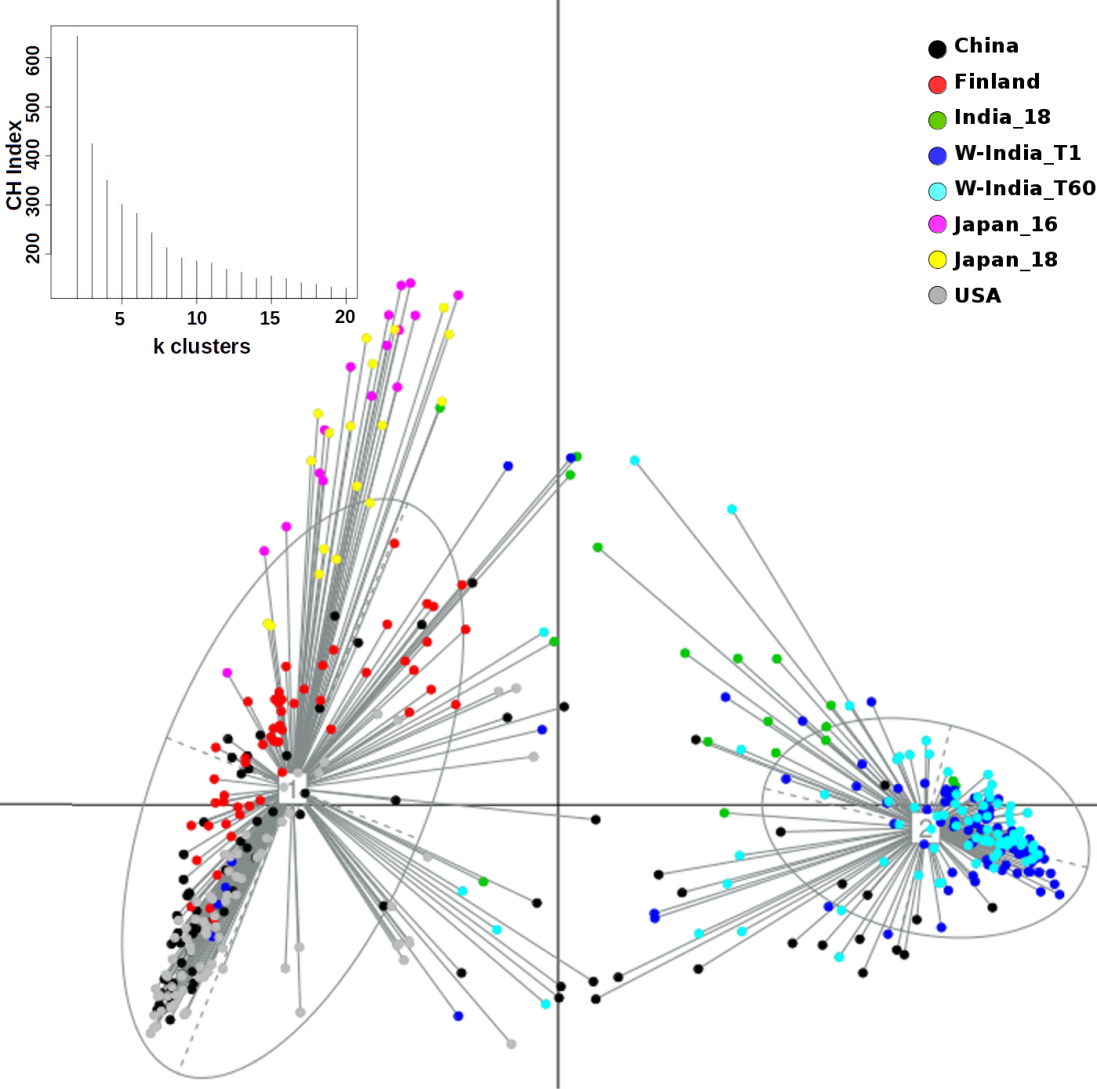
PCoA clustering of microbial abundance data based on Jensen Shannon divergence. Two distinct clusters were obtained. The bigger cluster corresponds to Cluster-1, while the smaller one depicts Cluster-2. The corresponding CH-index plot is depicted as inset within the main figure.

**Fig 4:**
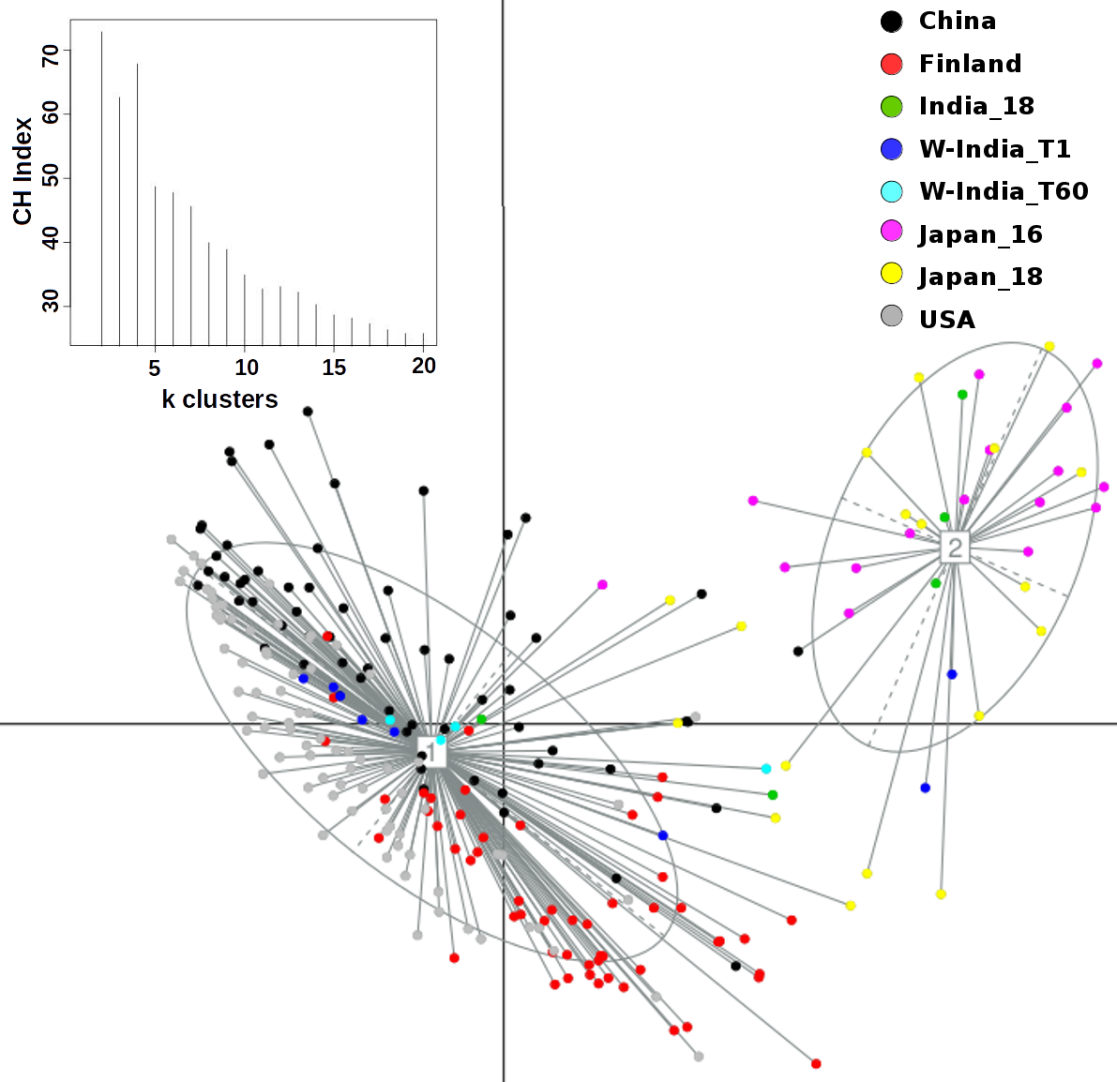
PCoA sub-clustering of samples in Cluster-1 (depicted in Fig 3). As indicated in the plot for CH-index (inset), two distinct sub-clusters were obtained. While samples in Cluster-1a are predominantly from three geographies, viz. USA, Finland and China, samples in Cluster-1b are predominantly from Japan.

**Fig 5:**
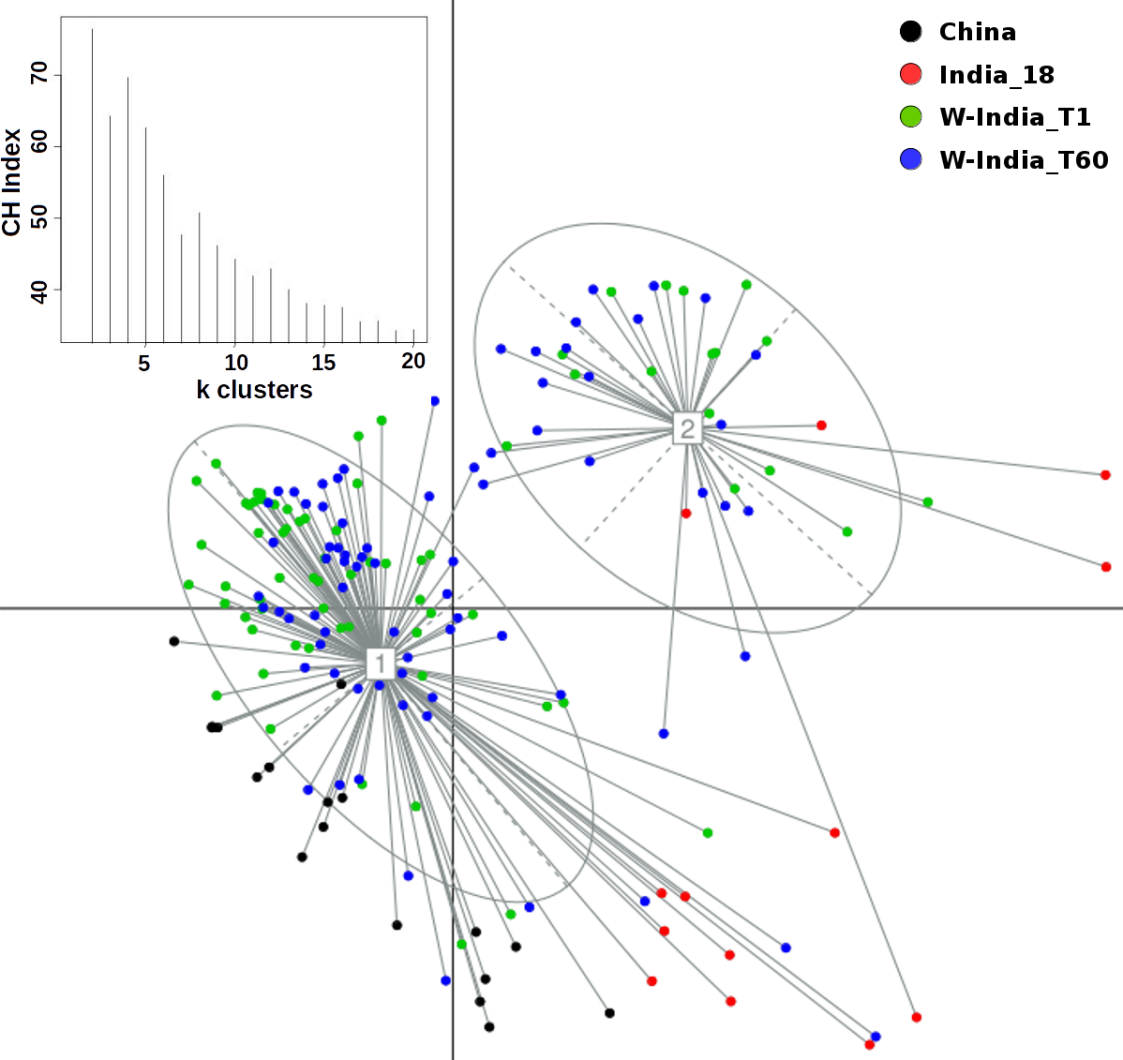
PCoA sub-clustering of samples in Cluster-2 (depicted in Fig 3). Although two distinct clusters were obtained, there is no visible separation of samples in terms of (dominant) geography or a sampling time-point (WIGM-T1 and WIGM-T60).

Principal Coordinate Analysis i.e. PCoA (using Jensen-Shannon divergence as a distance metric) was performed for comparing the taxonomic profiles in the Western Indian gut (obtained in the present study) with those obtained (from apparently healthy participants) in other geographies, viz. Japan, China, USA, and Finland[12,19,40–43]. S3 Table provides details of various studies from which these datasets were sourced. Results of this ordination analysis (depicted in Fig 3) indicate optimal grouping of microbiome sample profiles into two distinct clusters. Although, majority of non-Indian samples segregate into a distinct cluster (hereafter referred to as cluster 1), samples from Japan (in Cluster 1) appear to be spatially separated with respect to samples from other geographies in the same cluster. To confirm this observation, PCoA clustering was repeated using only the set of samples that were tagged to Cluster 1. The Calinski–Harabasz (CH) Index, a metric employed for inferring the statistically optimal number of clusters, obtained during this experiment (inset within Fig 4) indicates that samples in cluster 1 can be grouped optimally into either 2 or 4 clusters, the CH-Index value being almost similar for both cluster sizes. Fig 4 and S2 Fig depict the results of sub-clustering samples in cluster 1 into two and four groups, respectively. The two observations which are clearly evident from results in these figures are as follows. Irrespective of sub-clustering into two or four groups, the Japanese samples are observed to form a distinct sub-cluster with clear spatial segregation (of Japanese samples) with respect to samples belonging to China, US, and Finland. Samples of the latter three countries, however, do not clearly segregate as per their country of origin, suggesting a high level of similarity between their microbiome profiles. In contrast to results obtained by sub-clustering of samples in cluster 1, performing sub-clustering of samples in cluster 2 (which distinctly harboured >85% of Western Indian samples) indicates formation of 2 sub-clusters, with a CH-Index value of 2 being identified as most optimal (inset within Fig 5). However, in spite of sub-clustering, it is observed that samples in cluster 2 do not segregate/ sub-group in terms of sampling time-point (S3 Fig). Given these observations, overall three clusters, viz. the two sub-clusters (hereafter referred to as 1a and 1b) obtained from samples in cluster 1 (using a CH-Index value of 2) and cluster 2 (in its entirety) were considered for further comparative analysis.

### Comparison of core gut microbiota in individuals from different geographies

**Fig 6:**
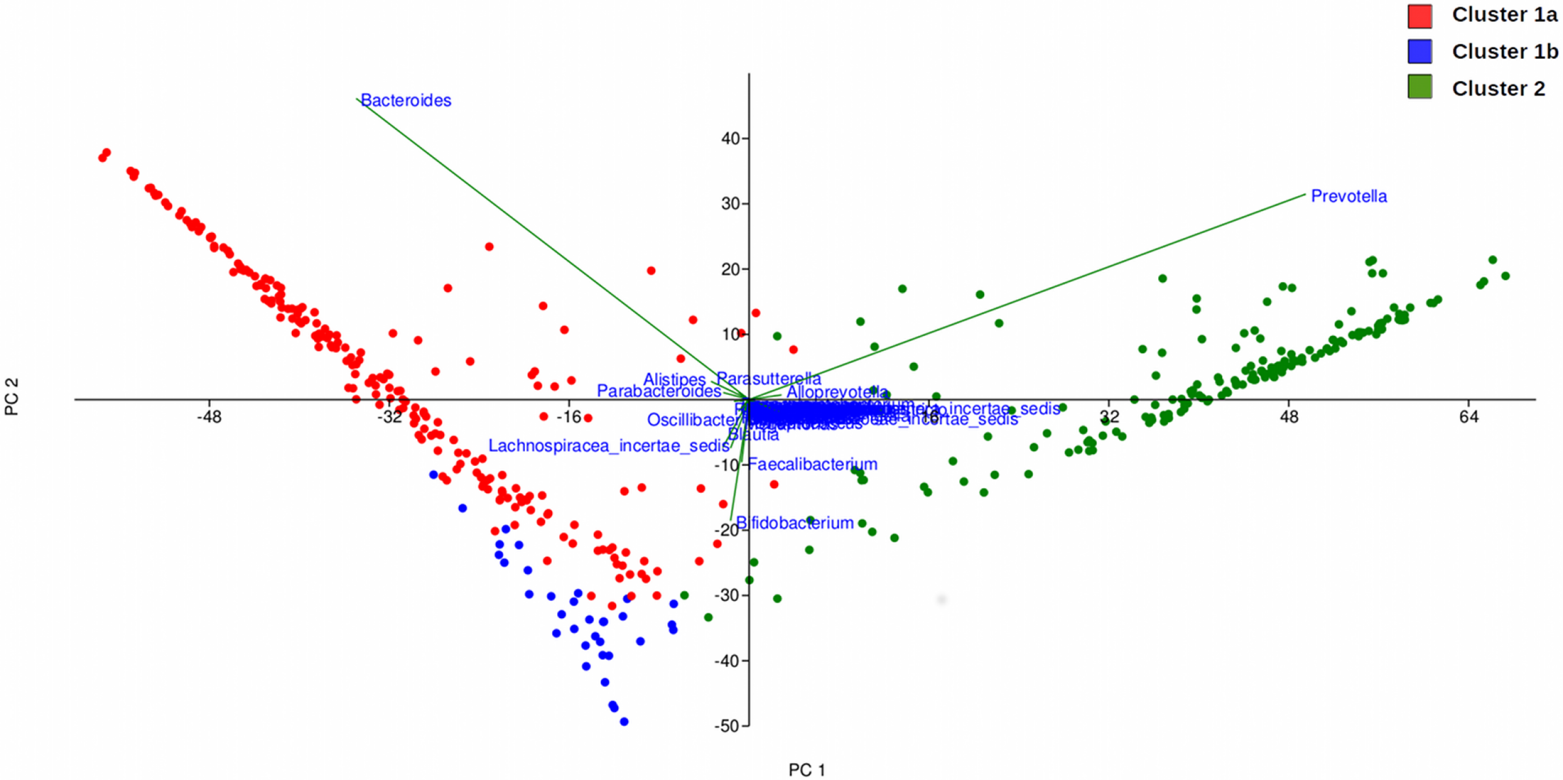
Principal component analysis of the abundance pattern of bacterial genera across the three clusters obtained during ordination analysis.

A rigorous boot-strapped method (a variant of the method described earlier[44]) was employed to identify, in each of the three clusters being compared, bacterial taxa that can be considered as 'core'. In brief, for each cluster being analysed, the identified 'core taxa' are those that are consistently present with a minimum threshold of abundance across various samples in that cluster. While *Bacteroides*, *Faecalibacterium*, and *Roseburia* are observed to constitute the core in cluster 1a, seven bacterial genera emerge as the core taxa in samples belonging to cluster 1b. These taxa included *Bacteroides*, *Faecalibacterium*, *Bifidobacterium*, *Blautia*, *Dorea*, *Lachnospiraceaincertaesedis*, and *Streptococcus*. In contrast, only two genera, namely, *Prevotella* and *Faecalibacterium* are identified as core in cluster 2 (S4 Fig). These results are consistent with those obtained by (a) performing a principal component analysis (PCA) of abundance pattern of taxa identified in various samples constituting all three clusters (Fig 6), (b) comparing the abundance pattern of the ten-most abundant genera and phyla in the three clusters (S5 and S6 Figs), and (c) employing Kruskal Wallis rank-sum test (coupled to a rigorous bootstrapped approach) for identifying genera whose abundance pattern is significantly different between the three clusters (Table 1). As seen in Fig 6, PCA analysis indicates *Bacteroides* and *Prevotella* as entities that drive/ define the separation of samples in clusters 1a and 2 respectively. Other core genera identified for cluster 1b are also consistent with results obtained in PCA analysis. The ratio of taxa belonging to phyla *Bacteroidetes* and *Firmicutes* appears to be a clear differentiator between the taxonomic profiles generated from various samples in each cluster (S5 and S6 Figs). Kruskal Wallis rank-sum test (with Benjamini-Hochberg corrected p-values < 0.001 at a False Discovery Rate of 0.0001) identified 80 genera as differentiators between the three clusters. Out of these, 21 genera (listed in Table 1) have a median percentage abundance of greater than equal to 0.2% in (samples belonging to) at least one of the clusters. Results in Table 1 appear to be in sync with results described earlier.

**Table 1:** List of differential genera.

List of Differentiating Genera	Median Percentage Abundance		
	Cluster_1a	Cluster_1b	Cluster_2
*Bacteroides*	*43*	9.8	0.7
*Roseburia*	*3*.*6*	0.3	2.4
*Alistipes*	*3*	0.2	0.1
*Parabacteroides*	*2*.*2*	0.3	0.1
*Ruminococccus*	*0*.*6*	0	0.2
*Gemmiger*	*0*.*5*	0	0.2
*Oscillibacter*	*0*.*5*	0.1	0
*Odoribacter*	*0*.*3*	0	0
*Ruminococcus2*	*0*.*2*	0	0
*Bifidobacterium*	0	*24*.*5*	0.4
*Faecalibacterium*	8.7	*12*.*8*	8.8
*Blautia*	1.1	*11*	0.3
*Lachnospiracea incertae sedis*	1.1	*10*.*2*	0.3
*Dorea*	0.3	*1*.*7*	0.2
*Streptococcus*	0.1	*1*.*6*	0.2
*Butyricicoccus*	0.1	*0*.*3*	0.1
*Prevotella*	0	0.1	*64*.*4*
*Alloprevotella*	0	0	*2*.*6*
*Sutterella*	0	0	*0*.*5*
*Dialister*	0	0	*0*.*5*
*Catenibacterium*	0	0	*0*.*2*

List of bacterial genera identified as having (significantly) different abundance between Cluster-1a, Cluster-1b and Cluster-2. These bacterial genera were identified using Kruskal-Wallis rank sum test (with Benjamini-Hochberg corrected p-values < 0.001 at a False Discovery Rate of 0.0001) coupled with a bootstrap approach (details in methods). Significantly different genera with median percentage abundance > = 0.2% in at least one of the three clusters are reported in the table. Numerals in blue represent the highest median abundance across the three clusters.

### Microbial community structure in terms of alpha-diversity measures in gut microbiome of individuals from different geographies

**Fig 7:**
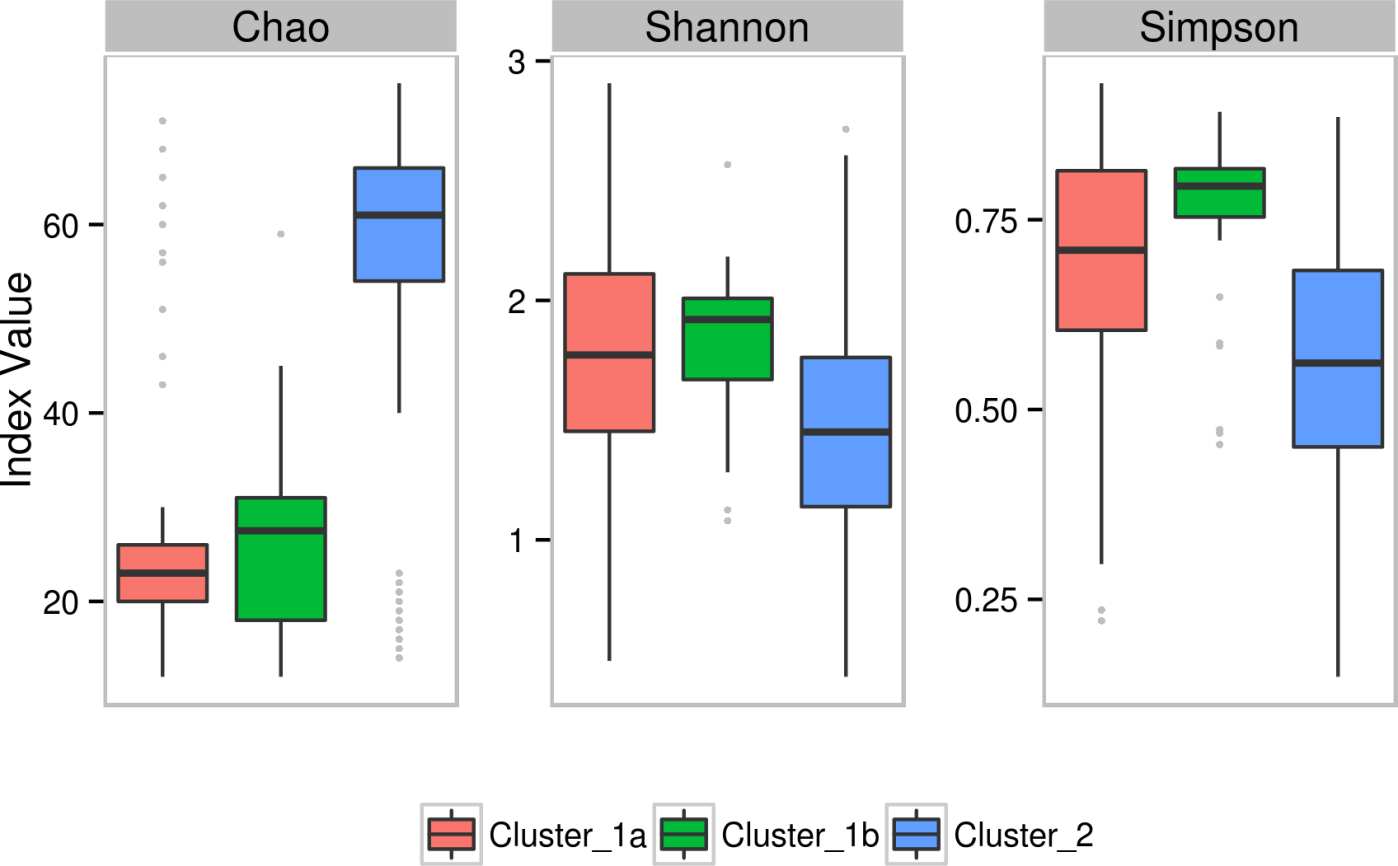
Box plots representing comparison between the diversity indices (Chao, Shannon and Simpson) for Cluster-1a, Cluster-1b and Cluster-2.

Employing various diversity metrics for inferring the structural aspects (viz. richness, diversity, and evenness) of the microbial community (in each of the three clusters) indicated the following interesting patterns. Analysis of various samples in the three clusters indicates that Western Indian microbiome samples (most of which are grouped in cluster 2) have significantly higher Chao values (a measure of richness) as compared to samples belonging to clusters 1a and 1b (Fig 7). However, values of Shannon index (a measure of diversity) and Simpson index (a measure of evenness) are significantly lower than that obtained with samples from other geographies (clusters 1a and 1b), indicating a biased community structure dominated by a few bacterial genera.

### Functional characteristics of gut microbiomes of individuals from different geographies

The functional potential of samples in each individual cluster was computationally inferred using Vikodak's Global Mapper module[26]. The (inferred) abundances of various pathways as well as pathway-classes predicted at 'pathway exclusion cut-off' (PEC) values of 50, 60, 70, 80 and 90 were subjected to Kruskal Wallis rank-sum test (with Benjamini-Hochberg corrected p-values < 0.001 at a False Discovery Rate of 0.0001) to identify significant differentiators between the three clusters. It may be noted that a PEC represents the threshold value (w. r. t. the proportion of constituent genes/ enzymes) that is used for inferring/ reporting a pathway (or pathway-class) to be present/ functional in the given sample. S4 Table and Table 2 list pathways and pathway classes that are found not only significantly differentiating buthad (predicted) non-zero median abundance values in at least one of the three clusters.

**Table 2:** List of differentially-abundant pathway classes.

Pathway classes that significantly differentiate between three clusters	(Median) Pathway Class Abundance (at PEC-70)			Vikodak Pathway Exclusion Cut-off (PEC) threshold				
	Cluster 1a	Cluster 1b	Cluster 2	50	60	70	80	90
Folding sorting and degradation	16059	17677	13281					
Membrane transport	11291	12558	7452					
Immune diseases	2366	3056	1393					
Signaling molecules and interaction	1572	2062	981					
Cell motility	4583	5383	2629					
Glycan biosynthesis and metabolism	28951	37763	18875					
Lipid metabolism	11971	15525	7795					
Metabolism of terpenoids and polyketides	17471	22573	12049					
Infectious diseases Bacterial	1178	1508	849					
Metabolism of other amino acids	9603	12268	7111					
Energy metabolism	46982	57159	33639					
Cell growth and death	7081	8942	5820					
Replication and repair	30060	36443	25571					
Translation	21133	24844	18784					
Transcription	2715	3342	2544					
Metabolism of cofactors and vitamins	40900	50412	49879					
Carbohydrate metabolism	104526	117542	151564					
Nucleotide metabolism	40603	48813	67972					
Amino acid metabolism	34599	38770	77688					
Xenobiotics biodegradation and metabolism	3971	4869	23764					
Biosynthesis of other secondary metabolites	454	543	4512					
Chemical structure transformation maps	3	2	176					

List of differentially abundant pathway-classes (identified between Cluster-1a, Cluster-1b and Cluster-2). Significantly different pathway classes were identified using Kruskal-Wallis rank sum test (with Benjamini-Hochberg corrected p-values < 0.001 at a False Discovery Rate of 0.0001) coupled with a bootstrap approach. Pathway-classes with significantly different median abundances in at least 99% of iterations are shown in this table. The last column titled ‘Vikodak Pathway Exclusion Cut-off (PEC) threshold’ indicates the PEC value thresholds at which the pathway-class was reported by Vikodak. Green indicates presence and red indicates absence.

Results in Table 2 and S4 Table indicate the following noticeable differences between the functional potential encoded by microbiomes belonging to each of the three compared clusters. The median abundance of the pathway class 'carbohydrate metabolism' is observed to be significantly higher for cluster 2 which overwhelmingly harbours samples from Indian subjects (Table 2). The pathway corresponding to 'Starch and sucrose metabolism' (belonging to pathway class 'carbohydrate metabolism') is also observed to be a distinct differentiator between the functional potential of the three clusters. Similarly, a number of metabolic pathways corresponding to pathway-classes 'glycan biosynthesis' and 'lipid metabolism' are observed to be over-represented in the microbiomes of cluster 1b (which are predominated by Japanese samples). In summary, the median abundances of the predicted pathways and pathway classes (emerging as significantly differentiating between the three clusters) appear to have a pattern of distinct functional divergence that grossly corresponds to dietary habits associated to geographies that each cluster represents.

## Discussion

Microbial consortia colonize various sites of the human body. The microbiome (i.e. the collective genetic repertoire of microbial consortia/ community inhabiting various human body sites) not only help in enriching our own functional potential, but also play a key role in our physiology, development, nutritional status, health, and immunity. Recent studies, which have deciphered various taxonomic and functional aspects pertaining to human gut microbiomes sampled across various geographies, indicate interesting associations between (geography-specific) dietary patterns and the composition of the gut microbiome sampled from subjects in specific geographies[15–19]. The present study attempts to expand the horizon of existing gut microbiome studies (aimed at cataloguing the repertoire of gut microbes) to the Western Indian geography. A reasonably participant cohort, comprising of 80 individuals residing within the geographical limits of the same city i.e. Ahmedabad, was recruited in the present study. Moreover, samples were taken from each participant at two time points (60 days apart). This study design ensured two aspects. As a country, India, with its large geographical spread, has significant variations with respect to dietary habits as well as climatic conditions[21,22] Given that objective of the present study was to establish base-line data that comprehensively represents at least one of the various dietary preferences prevalent in India, the selection of a subject cohort was restricted to a single region i.e. Western India (particularly the city of Ahmedabad). Obtaining samples at two time points was done with the objective of studying the dynamics and stability aspects of human gut microbiomes within the same individual. A comparison of microbiome samples obtained from the same individuals at two time points indicated no significant alteration of microbiome community structure over time (Fig 1). Results of ordination analysis also indicates grouping of samples from both time-points into the same cluster i.e. cluster 2 (Figs 3 and 5). Results of (further) sub-clustering of samples in cluster 2 also indicate the high level of similarity of samples obtained at both time points (S3 Fig). Further studies will however be required to explore the vast heterogeneity of gut microbial communities within individuals from various parts of India.

**Fig 8:**
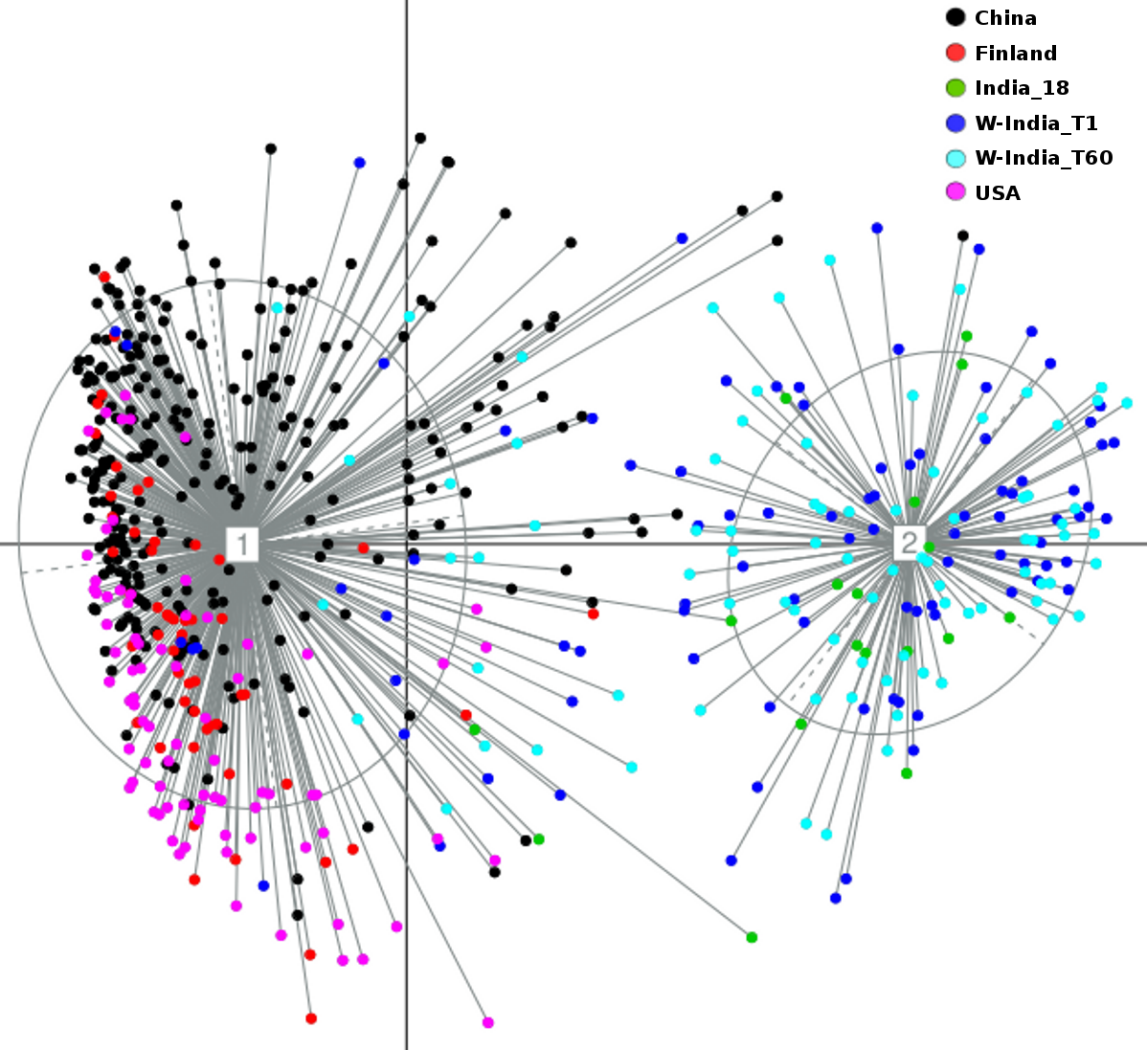
PCoA clustering of microbial abundance data that was generated using only the V3 region of the 16S rRNA gene sequences in various datasets used for comparison.

Results of various ordination experiments performed in this study primarily indicate (sub-grouping)and spatial segregation of microbiome samples into two distinct clusters. Although this may be indicative of some extent of correlation of microbiome profiles with dietary preferences prevalent in respective (compared) geographies, the possible contribution of confounding factors (viz. sequencing platform, PCR primers, DNA extraction protocols, target region of the 16S rRNA gene, and various sample collection/ handling methods used in different studies) to the observed spatial segregation cannot be ruled out[45]. In order to check the impact of minimising (to whatever extent possible) the effect/ bias of the stated confounding factors on the clustering pattern of microbiome samples, PCoA clustering was redone using only those taxonomic profiles that were generated using only the V3 variable region. The latter region was the common variable region within sequencing reads from most of the studies used in this comparison (S3 Table). Samples from the Japanese cohort (which employed used the V1-V2 region in their study) were omitted from this analysis. Similar to the overall clustering pattern (as observed in Fig 3), results of this ordination analysis (using only the V3 regions) again indicate optimal grouping of microbiome sample profiles into two distinct clusters (Fig 8). In addition to the re-iteration of the observation that Western Indian samples grouping into a distinct separate cluster, it is interesting to note that samples from the 2 studies in Western India, cluster together despite utilizing different protocols in the two labs.

In the present study, an interesting pattern of correlation between bacterial community structure (in terms of species richness) and the functional repertoire of enzymes contributing to various identified pathways (inferred as being present/ functional) was observed. Results in S4 Table indicate the presence of several differentiating pathways having a higher median abundance in the Western Indian samples (cluster 2). These included starch and sucrose metabolism, amino sugar and nucleotide sugar metabolism, arginine and proline metabolism, glycine serine and threonine metabolism, cysteine and methionine metabolism, propanoate metabolism, porphyrin and chlorophyll metabolism. Interestingly, the median abundances of these pathways are null in the other two clusters. For a pathway to be reported by Vikodak (at PEC value of 70) as present/ functional in a sample, various microbes present in a sample should encode at least 70% of genes/ enzymes constituting that pathways. A null median abundance of a pathway in a particular cluster indicates that greater than 50% of samples in that cluster lacked the necessary microbial diversity to encode for the minimum quorum of genes/ enzymes required for the functioning of the pathway. Given this, it may be logical to expect (from results depicted in S4 Table) that samples in cluster 2 (followed by cluster 1b) to have relatively higher microbial richness as compared to that in cluster 1. Results of diversity (Fig 7) are observed to be perfectly in sync with this line of thought. Microbiome samples in the 'Western India-specific' cluster have significantly higher Chao values (a measure of richness) as compared to samples from other geographies (clusters 1a and 1b).

Besides the presence of a distinct cluster of genera which have known functional roles in metabolism of butyrate, acetate, and proprionate (as seen in results depicted in Fig 2), analysis of correlations between the abundance patterns of various genera identified in the Western Indian gut microbiomes also revealed the presence of a specific subset of ‘independent’ genera. The abundances of these genera are observed to share no significant correlation with *Prevotella* as well as with the sub-group of genera with known butyrate metabolism capabilities. Most of these ‘independent’ genera, comprising of *Dialister*, *Catenibacterium*, *Allisonella*, *Hallella*, *Neisseria*, *Oribacterium*, *Megamonas*, *Streptophyta*, *Klebsiella*, *Olsenella*, *Succinivibrio*, *Rothia*, and *Turicibacter*, are known to be potential pathogens occurring in meagre proportions in a typical healthy human gut. The role of these genera in the Western Indian gut microbiome remains to be probed.

Two earlier published studies[46,47] had attempted elucidating cross-geography inferences with respect to the relationship between microbiome composition and the abundance as well as diversity of the carbohydrate/ xenobiotic metabolizing capabilities. In this context, it is interesting to note that Western Indian samples in cluster 2 appear to be highly enriched with bacterial genera that encode functions associated with three other pathway classes, namely 'Amino acid metabolism', 'Xenobiotics biodegradation and metabolism', and 'Biosynthesis of other secondary metabolites'. Earlier studies[48,49] have indicated associations between the abundances of taxa belonging to phylum *Bacteroidetes* with the abundances of two functions, namely, degradation of antibiotics (a classic example of a xenobiotic) and metabolism of complex glycans. The *Prevotella* (i.e. *Bacteroidetes*-rich) Western Indian gut microbiomes appear to justify this hypothesized association. These findings justify the need for initiating further 'Whole Genome Sequencing' based studies to probe/ understand associations between various predicted functions and the structure of the Western Indian microbiome.

Although comparative analysis of microbiome samples (spanning 5 geographies) performed in this study (S3 Table) indicate some extent of correlation of microbiome profiles with dietary preferences prevalent in specific geographies, it is likely that apart from diet, several other factors play a role in shaping of the gut microbiome.Further studies will be required to assert this correlation.

## Declarations

### Ethics approval and consent to participate

The protocol used in this study was approved by the Anveshhan Independent Ethics Committee (situated at B-8, Simandhar residency, Near Gulab Tower, Behind Utopia school, Thaltej, Ahmedabad-380054, Gujarat, India) with Registration Number ECR/24/Indt/GJ/2013/RR-16. This committee is associated with the Veeda Clinical Research, Ahmedabad, India.

### Consent for publication

Healthy volunteers from Ahmedabad city in Gujarat were individually provided with a subject informed consent document that provided complete information on the sampling procedure and the objectives of the study.

## Supporting information

S1 Fig A rarefaction plot of samples for the two time-points IGM-T1 and IGM-T60 involved in the present study. (TIF)

S2 Fig PCoA sub-clustering of Cluster-1 obtained in Fig 3. Apart from the two distinct sub-clusters (Fig 4), Cluster-1 can also be separated into four distinct clusters (indicated in CH-index plot in inset).
(TIF)

S3 Fig PCoA sub-clustering of Cluster-2 obtained in Fig 3. Apart from the two distinct sub-clusters (Fig 5), Cluster-2 can also be separated into four distinct clusters (indicated in CH-index plot in inset).
(TIF)

S4 Fig A venn diagram representing the 'core' genera constituting the three clusters obtained during ordination analysis. (TIF)

S5 Fig **Area curve representing the bacterial genera across Cluster-1a, Cluster-1b and Cluster-2.** Abundance data of the most abundant taxa across the three clusters was included to plot the curve.
(TIF)

S6 Fig **Area curve representing the bacterial phyla across Cluster-1a, Cluster-1b and Cluster-2.** Abundance data of the most abundant taxa across the three clusters was included to plot the curve.
(TIF)

S1 Table Metadata corresponding to various subjects enrolled in the present study. (PDF)

S2 Table Taxonomic profiles of Indian gut microbiomes. (PDF)

S3 Table Details of various studies (and corresponding number of samples) that were used for comparative analysis with Indian microbiome samples obtained in the present study. (PDF)

S4 Table **List of differentially abundant pathways (identified between Cluster-1a, Cluster-1b and Cluster-2).** Significantly different pathways were identified using Kruskal-Wallis rank sum test (with Benjamini-Hochberg corrected p-values < 0.001 at a False Discovery Rate of 0.0001) coupled with a bootstrap approach. Pathways with significantly different median abundances in at least 99% of iterations are shown in this table. For ease of readers, the corresponding pathway classes are also indicated. The last column titled ‘Vikodak Pathway Exclusion Cut-off (PEC) threshold’ indicates the PEC value thresholds at which the pathway was reported by Vikodak. Green indicates presence and red indicates absence.
(PDF)
