# Supplementary figures and images for: A snapshot of gut microbiota of an adult urban population from Western region of India
Source: PLoS One. 2018 Apr 6;13(4):e0195643. doi: 10.1371/journal.pone.0195643 (PMC5889170; doi:10.1371/journal.pone.0195643)

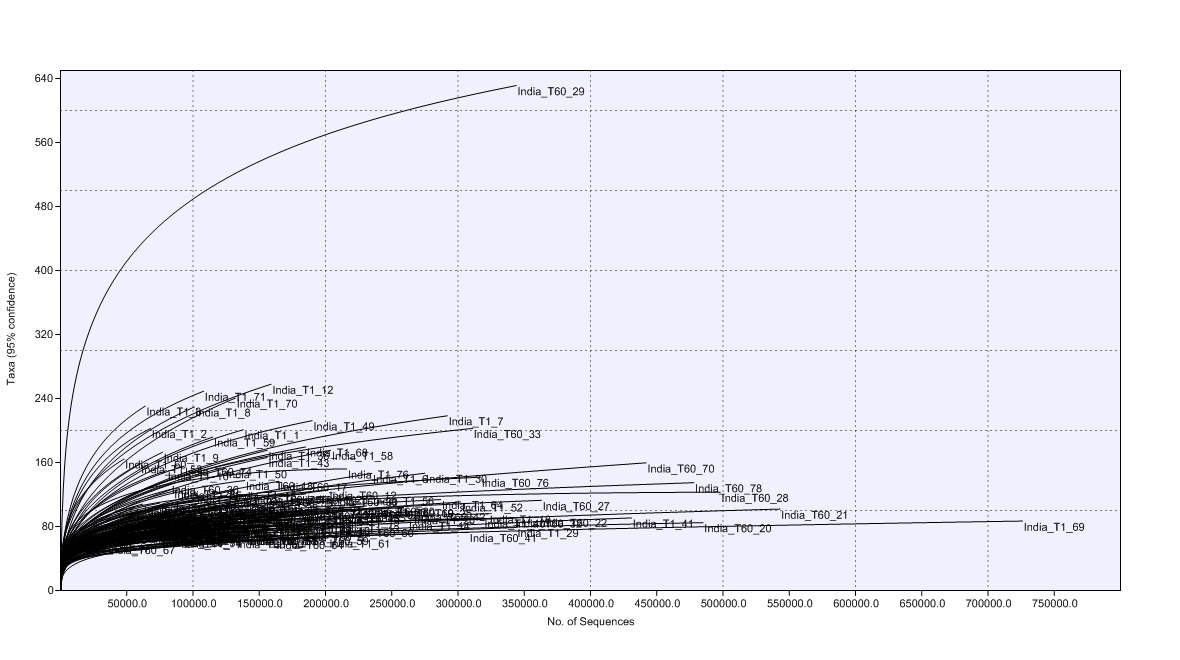

Supplement: S1 Fig — (TIF) [file pone.0195643.s001.tif]

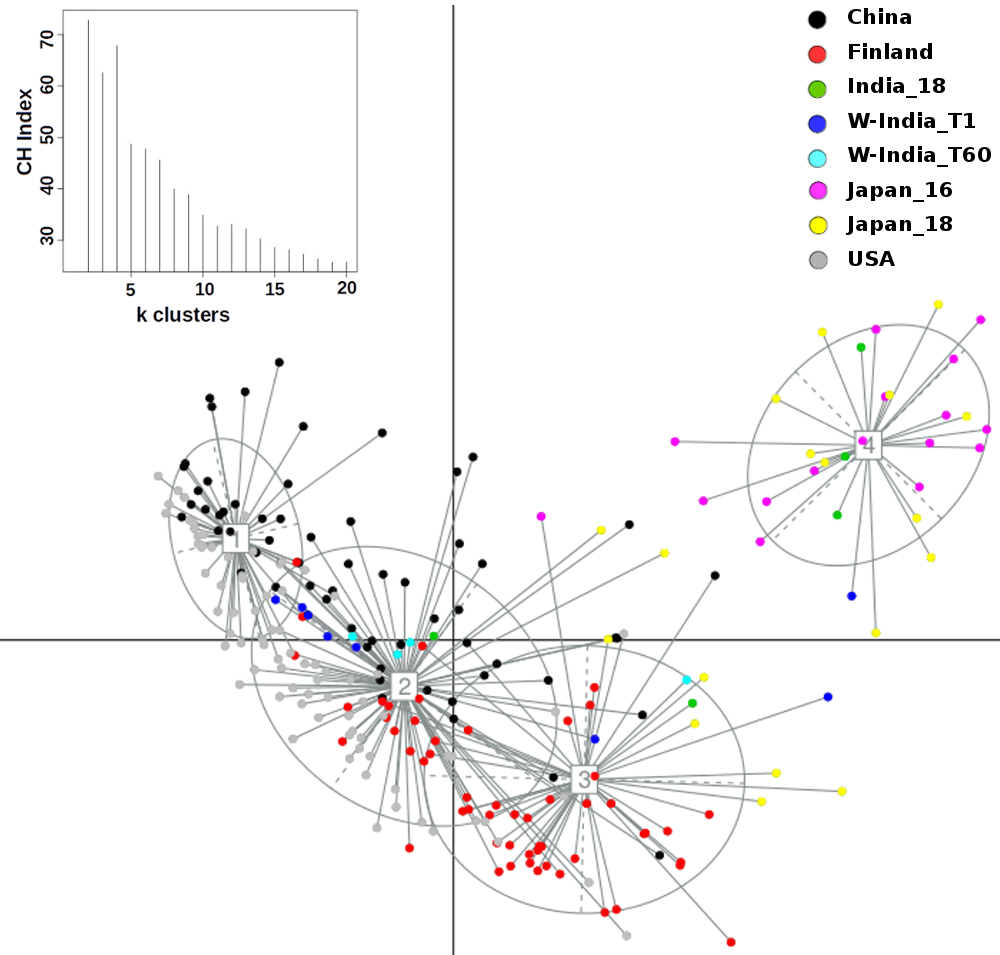

Supplement: S2 Fig — Apart from the two distinct sub-clusters (Fig 4), Cluster-1 can also be separated into four distinct clusters (indicated in CH-index plot in inset). (TIF) [file pone.0195643.s002.tif]

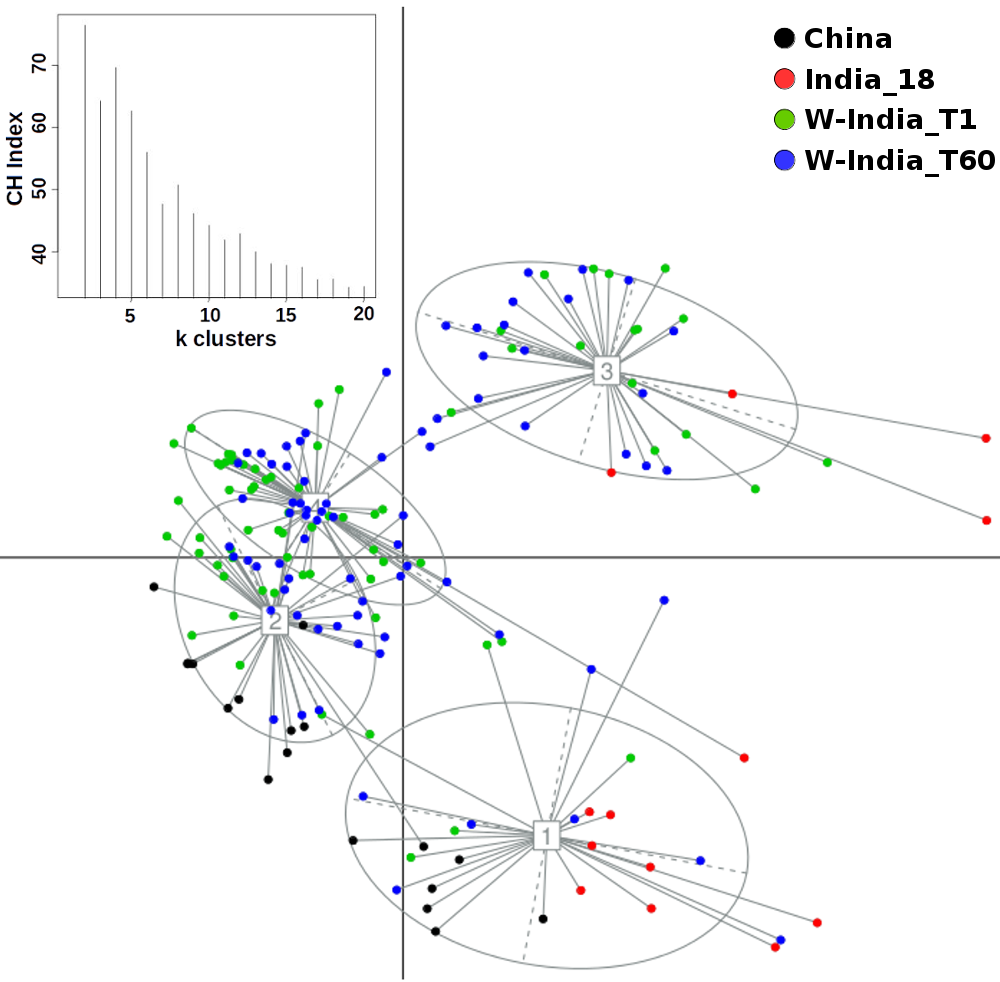

Supplement: S3 Fig — Apart from the two distinct sub-clusters (Fig 5), Cluster-2 can also be separated into four distinct clusters (indicated in CH-index plot in inset). (TIF) [file pone.0195643.s003.tif]

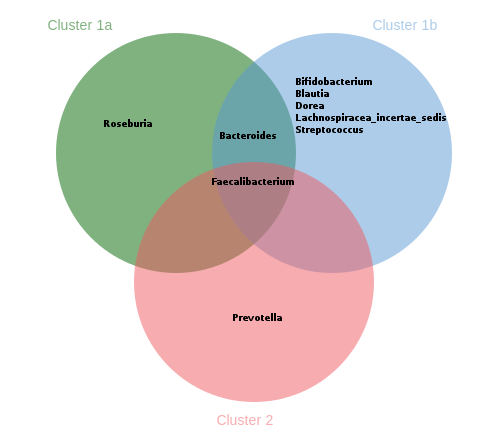

Supplement: S4 Fig — (TIF) [file pone.0195643.s004.tif]

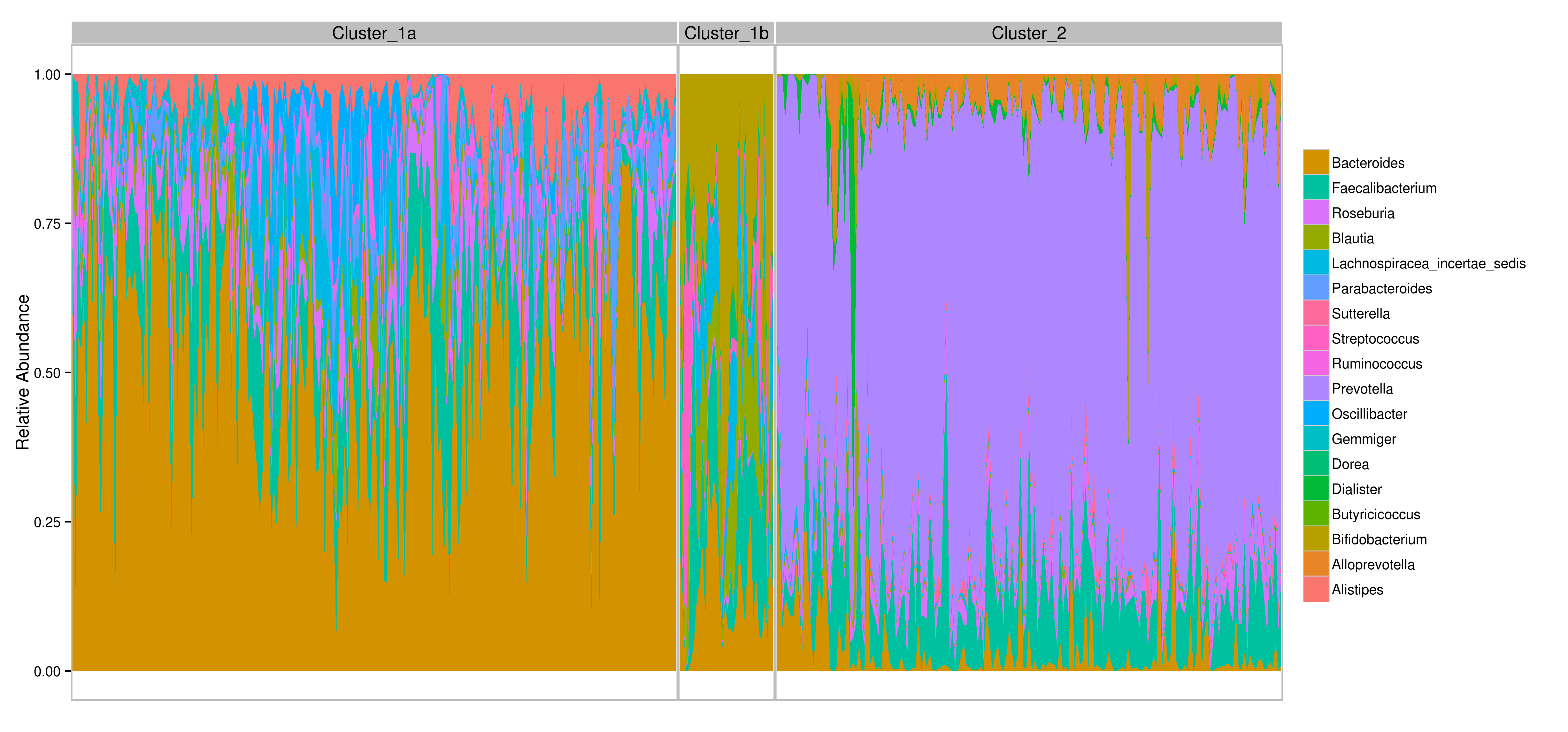

Supplement: S5 Fig — Area curve representing the bacterial genera across Cluster-1a, Cluster-1b and Cluster-2. Abundance data of the most abundant taxa across the three clusters was included to plot the curve. (TIF) [file pone.0195643.s005.tif]

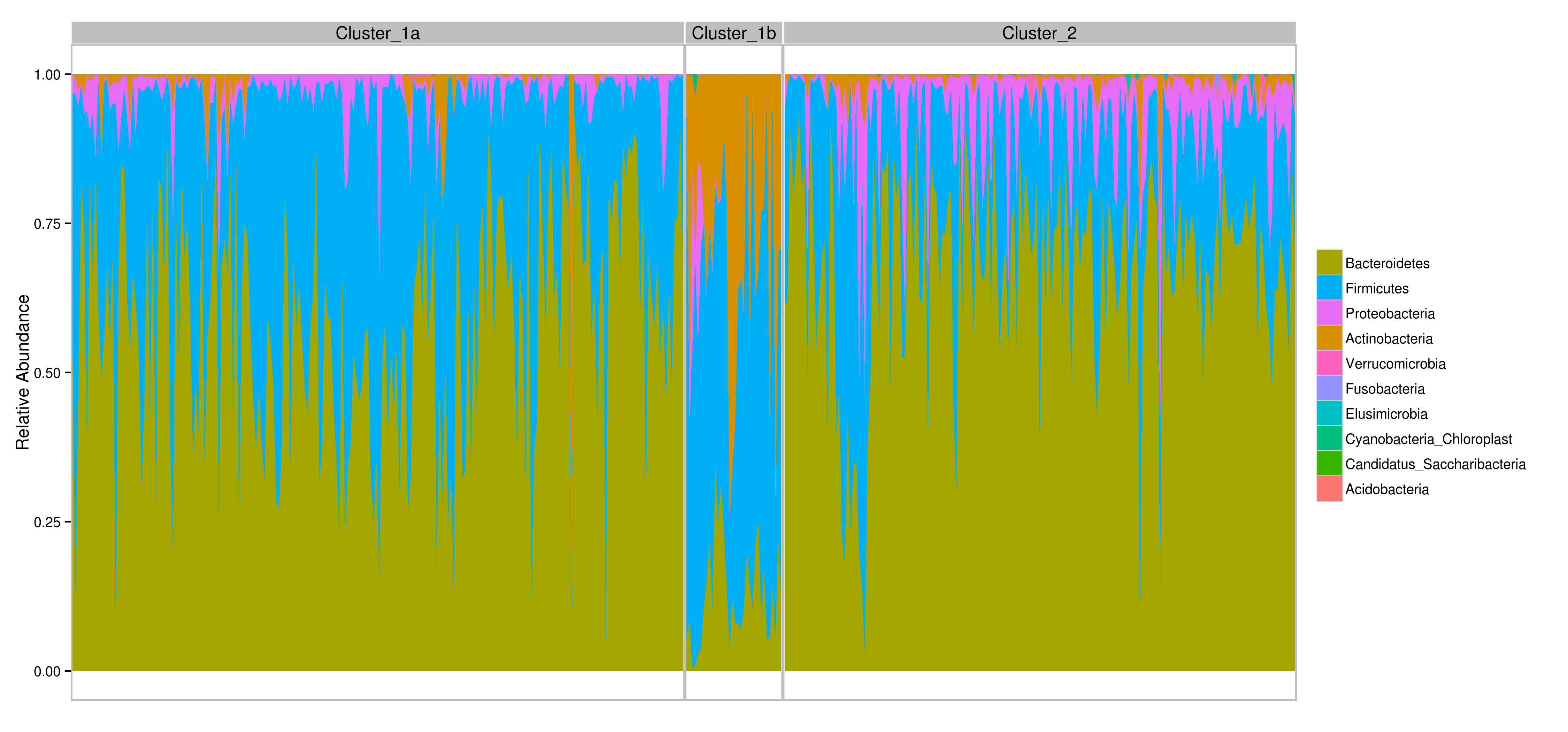

Supplement: S6 Fig — Area curve representing the bacterial phyla across Cluster-1a, Cluster-1b and Cluster-2. Abundance data of the most abundant taxa across the three clusters was included to plot the curve. (TIF) [file pone.0195643.s006.tif]
