# Supplementary material for: A snapshot of gut microbiota of an adult urban population from Western region of India
Source: PLoS One. 2018 Apr 6;13(4):e0195643. doi: 10.1371/journal.pone.0195643 (PMC5889170; doi:10.1371/journal.pone.0195643)
Supplement: S1 Table — (PDF) [file pone.0195643.s007.pdf]

S1 Table: Metadata corresponding to various subjects enrolled in the present study

Subj. ID	Sex	Asian	Diet (V - Veg; NV - NonVeg)	Radial Pulse (/min)		Sitting Blood Pressure (mm Hg)		Respiratory rate (/min)		Oral Temperature (C)	
				Day01	Day60	Day01	Day60	Day01	Day60	Day01	Day60
1	Male	Y	V	78	72	122/82	117/72	16	17	36.4	36.4
2	Male	Y	NV	72	72	120/76	114/70	16	18	36	36.5
3	Male	Y	NV	76	72	122/72	114/68	15	15	36.3	36.7
4	Male	Y	V	70	72	116/74	120/76	14	16	36.4	36.6
5	Male	Y	NV	76	76	118/76	118/78	15	18	36.1	36.2
6	Male	Y	V	76	80	124/82	122/78	14	17	36.4	36.8
7	Male	Y	V	68	74	120/72	118/76	15	16	36.4	36.5
8	Male	Y	NV	70	70	114/72	114/76	16	16	36.5	36
9	Male	Y	NV	68	70	116/72	124/80	14	18	36.2	36.8
10	Male	Y	V	74	68	122/80	126/80	17	16	36.6	36.2
11	Female	Y	NV	74	70	116/74	112/68	18	14	36.1	36.2
12	Female	Y	NV	82	68	120/80	116/76	14	18	36.2	36.2
13	Female	Y	NV	86	70	116/78	112/70	18	16	36.7	36.4
14	Female	Y	NV	90	76	112/74	118/78	16	15	36.4	36.4
15	Male	Y	NV	76	72	116/74	118/74	15	15	36.6	36.1
16	Male	Y	NV	72	68	116/76	122/76	14	16	36.1	36.4
17	Female	Y	V	84	72	122/80	122/80	14	16	36.1	36.2
18	Female	Y	NV	90	76	126/82	118/74	14	17	36.4	36.2
19	Female	Y	V	76	68	116/78	126/80	18	14	36.7	36
20	Female	Y	V	78	76	114/76	118/76	18	14	36.3	36.2
21	Male	Y	NV	72	64	114/78	122/80	13	18	36.1	36.7
22	Male	Y	V	76	76	126/80	118/72	16	16	36.6	36.4
23	Male	Y	NV	72	68	118/76	116/76	16	18	36.6	36.1
24	Male	Y	NV	72	66	120/76	114/66	14	16	36.4	36.7
25	Male	Y	NV	72	78	120/76	124/80	16	17	36.4	36.8
26	Male	Y	NV	72	70	118/76	116/72	14	14	36.1	36.2
27	Male	Y	NV	70	70	116/76	124/74	14	16	36.6	36.2
28	Male	Y	NV	76	70	122/72	128/80	16	16	36.1	36.2
29	Male	Y	NV	72	72	120/76	116/70	14	16	36.2	36.4
30	Male	Y	NV	70	68	118/72	116/74	16	18	36.1	36.6
31	Female	Y	V	74	76	120/76	118/80	16	16	36.2	36.2
32	Female	Y	V	78	68	114/76	114/72	16	14	36.5	35.8
33	Female	Y	NV	76	70	118/74	120/80	14	18	36.3	36
34	Female	Y	NV	92	70	124/82	116/76	18	16	36.1	36
35	Female	Y	NV	90	76	128/82	118/72	14	18	36.2	36.2
36	Female	Y	NV	74	72	114/72	116/78	15	15	36.7	36.6
37	Female	Y	V	72	68	118/76	118/74	16	16	36.7	36.6
38	Female	Y	NV	78	70	116/74	116/74	18	16	36.7	36.9
39	Female	Y	NV	72	74	124/82	122/80	18	16	36.5	36.2

40	Female	Y	NV	72	72	122/80	114/74	14	16	36.8	36.2
41	Male	Y	NV	76	68	124/80	116/72	14	15	36.5	36.1
42	Male	Y	NV	76	70	124/80	122/70	17	14	36	36.2
43	Male	Y	NV	72	64	116/76	118/68	14	12	36.6	36.1
44	Male	Y	V	70	72	116/72	120/76	14	14	36.1	36.1
45	Male	Y	NV	70	74	116/72	120/80	16	18	36.2	36.7
46	Male	Y	NV	76	66	116/72	122/78	15	12	36.1	36.4
47	Male	Y	NV	70	70	118/72	118/72	16	16	36.4	36.4
48	Male	Y	NV	68	68	114/72	118/74	17	14	36	36.1
49	Male	Y	NV	70	74	118/72	124/78	16	16	36.1	36.5
50	Male	Y	NV	74	66	120/76	120/74	15	16	36.1	36.2
51	Male	Y	NV	70	68	120/74	120/80	16	16	36.3	36.2
52	Male	Y	V	70	72	118/70	118/74	14	14	36.9	36.4
53	Female	Y	NV	70	70	112/74	116/72	18	15	36.1	36
54	Female	Y	NV	76	76	118/80	120/80	14	14	36.7	36.4
55	Female	Y	NV	72	72	120/74	116/76	18	14	36.2	36.6
56	Female	Y	V	76	66	122/80	124/72	16	16	36.2	36.1
57	Female	Y	NV	78	70	114/72	124/76	18	18	36.5	36.2
58	Female	Y	NV	70	72	120/76	126/80	14	16	36.4	36.4
59	Female	Y	NV	74	64	114/72	128/82	16	14	36.8	36.5
60	Male	Y	V	76	70	116/72	126/80	14	16	36.2	36.4
61	Male	Y	V	70	68	116/78	116/70	14	17	36.4	36.8
62	Male	Y	NV	72	66	122/82	124/70	15	16	36.1	36.2
63	Male	Y	NV	74	72	124/82	118/68	18	16	36.2	36.4
64	Male	Y	NV	72	76	116/72	120/76	14	17	36.1	36.8
65	Male	Y	NV	72	74	116/74	112/70	15	13	36.1	36.3
66	Male	Y	Veg/NV	72	68	116/76	120/76	15	16	36.6	36.4
67	Male	Y	NV	68	72	116/72	118/68	14	14	36.1	36.2
68	Male	Y	NV	74	70	116/72	122/72	14	12	36.6	36.1
69	Male	Y	V	74	70	122/76	124/76	16	14	36.4	36.2
70	Male	Y	NV	72	74	118/76	122/72	16	16	36	36.4
71	Female	Y	V	72	70	122/76	122/70	14	18	36.3	36.5
72	Female	Y	V	78	62	112/74	118/70	16	12	36.3	36.5
73	Female	Y	NV	72	74	118/74	120/76	16	16	37.2	36.4
74	Female	Y	NV	68	78	124/76	118/76	14	17	36.9	36.3
75	Female	Y	NV	76	70	118/70	114/76	15	16	36.9	36.1
76	Female	Y	V	78	62	110/70	120/68	14	16	36.4	36.4
77	Female	Y	NV	78	66	114/72	116/66	18	12	36.5	36.2
78	Male	Y	V	70	76	114/70	126/72	16	18	36.7	36.4
79	Female	Y	NV	74	74	110/68	118/68	17	12	36.1	36.1
80	Male	Y	NV	70	68	118/72	114/74	15	15	36.4	36.1

Age (Yrs)	Height (cm)	Weight (Kg)	BMI (Kg/m²)	Pre/ Post menopausal	Habits
30	176	78.6	25.37		
33	168	74.5	26.4		Tea [Taken 3 before days]
29	170	71	24.57		Pan Masala (Non Tobacco) [Left 4 months back]
26	186.5	66.6	19.15		
32	175	74	24.16		
33	164	62.2	23.13		
32	166	65.3	23.7		Tea(one cup/day)
27	157	60	24.34		Tea (one cup/ day)
32	153	50	21.36		
41	167	73	26.18		Non tobacco pan masala; (2/ day; Left 6 months ago)
30	147	46.5	21.52	Pre	Tea (one cup/ day)
33	146.5	53	24.69	Pre	Tea (one cup/ day)
30	154	48.6	20.49	Pre	
39	155	49.3	20.52	Pre	
35	166	69.5	25.22		Tea (two cups/ day)
31	162	56.5	21.53		
42	162	74.5	28.39	Post	Tea (two cups/ day)
32	158.5	72	28.66	Pre	Tea (one cup/ day)
33	154	70	29.52	Pre	
28	157	65	26.37	Pre	
39	160	55	21.48		
40	173	81	27.06		Non tobacco pan masala (2/day); Left since 3 years
30	163	62.8	23.64		Tea (one cup/ day)
29	157	45.8	18.58		Tea (one cup/ day)
40	163	54.8	20.63		
38	170	56.5	19.55		
29	170	55.3	19.13		Tea (one cup/ day)
27	176	58	18.72		
41	164	62.3	23.16		
40	164	73.5	27.33		Tea (one cup/ day)
28	155	70	29.14	Pre	Tea (one cup/ day)
23	147	49	22.68	Pre	Tea (one cup/ day)
37	152	47.7	20.65		Tea (one cup/ day)
37	150	63.8	28.36	Pre	Tea (one cup/ day)
37	151	52	22.81	Pre	Tea (one cup/ day)
34	149	45.8	20.63	Pre	Tea (one cup/ day)
40	150	67	29.78	Pre	Tea (two cups/ day)
35	149	55	24.77	Pre	Tea (one cup/ day)
35	147.5	60.6	27.85	Pre	Tea (two cups/ day)

35	153	68	29.05	Pre	Tea (two cups/ day)
36	167.5	83.5	29.76		
39	157	55.6	22.56		Tea (one cup/ day)
34	161	56	21.6		Non tobacco pan masala (2/day; Left 12 months ago)
30	188	67.5	19.1		Tea (two cups/ day)
39	168	63	22.32		
35	161.5	68.2	26.15		Tea (one cup/ day); Non tobacco pan masala; (left since 4 years)
40	165	52	19.1		Non tobacco pan masala (1/day; Left 12 months ago)
37	165	51.6	18.95		Tea (two cups/ day)
32	171.5	64	21.76		Tea (two cups/ day)
36	152	62.5	27.05		Tea (one cup/ day)
31	162.5	70.2	26.58		
28	163.5	64.3	24.05		Tea (one cup/ day); Non tobacco pan masala; (left since 7 years)
38	138	45.5	23.89	Pre	Tea (two cups/ day)
25	155	60.7	25.27	Pre	Tea (two cups/ day)
29	144	52.7	25.41	Pre	Tea (one cup/ day)
33	152	63.5	27.48	Pre	Tea (one cup/ day)
38	146	51.2	24.02	Pre	Tea (one cup/ day)
43	149	55.3	24.91	Pre	
44	154	71	29.94	Pre	Tea (one cup/ day)
44	166	75	27.22		
39	160	54.6	21.33		
44	166	72	26.13		
31	169	76	26.61		
26	164	64.5	23.98		
37	154.5	46.8	19.61		
41	170	54	18.69		
34	158	58.6	23.47		
31	163	54	20.32		Non tobacco pan masala; (left 6 years ago)
30	171	77	26.33		
34	157	52.8	21.42		
38	149	45.8	20.63	Pre	Tea (two cups/ day)
38	148	46.5	21.23	Pre	Tea (two cups/ day)
26	156	64.2	26.38	Pre	Tea (two cups/ day)
38	143	48.5	23.72	Pre	Tea (two cups/ day)
35	150.5	67	29.58	Pre	Tea (two cups/ day)
27	146.5	54.5	25.39	Pre	Tea (two cups/ day)
30	144	48.5	23.39	Pre	Tea (two cups/ day)
41	163.5	56.5	21.14		Tea (two cups/ day)
44	147	48.5	22.44	Pre	Tea (two cups/ day)
31	165	56.7	20.83		Tea (one cup/ day)
