# Supplementary material for: A snapshot of gut microbiota of an adult urban population from Western region of India
Source: PLoS One. 2018 Apr 6;13(4):e0195643. doi: 10.1371/journal.pone.0195643 (PMC5889170; doi:10.1371/journal.pone.0195643)
Supplement: S2 Table — (PDF) [file pone.0195643.s008.pdf]

S2 Table: Taxonomic profiles of Indian Gut Microbiomes

Genus	W_India_T1_1	W_India_T1_10	W_India_T1_11
Acinetobacter	0.00072	0.00000	0.00000
Actinomyces	0.00000	0.00000	0.00228
Akkermansia	0.00000	0.00000	0.00000
Alistipes	0.00644	0.23347	0.44199
Allisonella	0.01859	0.01222	0.00457
Alloprevotella	9.60152	0.00489	8.48566
Anaerostipes	0.00000	0.00122	0.02284
Bacteroides	0.12014	27.66870	0.78118
Barnesiella	0.00072	0.00122	0.00114
Bifidobacterium	0.22313	0.74318	0.90453
Bilophila	0.00000	0.00367	0.03997
Blautia	0.23242	0.60628	0.66355
Butyricicoccus	0.18951	0.04767	0.14733
Butyricimonas	0.00143	0.00000	0.00685
Campylobacter	0.00000	0.00122	0.00228
Catenibacterium	0.23957	0.00000	1.15008
Clostridium_IV	0.00000	0.02322	0.16446
Clostridium_sensu_stricto	1.59477	0.15646	0.09137
Clostridium_XI	0.66079	0.09412	0.09822
Clostridium_XlVa	0.07509	0.23958	0.09708
Clostridium_XlVb	0.00501	0.03178	0.08566
Collinsella	0.05578	0.00367	0.33577
Comamonas	0.00000	0.00000	0.00000
Coprococcus	0.05221	0.25180	0.79032
Dialister	1.18213	0.16379	0.76063
Dorea	0.10084	0.25547	0.21129
Elusimicrobium	0.00000	0.00000	0.00000
Enterobacter	0.00143	0.00000	0.00343
Erysipelotrichaceae_incertae_sedis	0.02718	0.00000	0.30265
Escherichia_Shigella	0.05078	0.03667	0.19415
Faecalibacterium	3.52351	7.24475	7.90434
Flavonifractor	0.00072	0.00244	0.00000
Fusicatenibacter	0.01502	0.17235	0.05482
Fusobacterium	0.00000	0.00000	0.00799
Gemella	0.00000	0.00000	0.00343
Gemmiger	0.18308	0.14912	0.18673
Granulicatella	0.00000	0.00000	0.00457
Haemophilus	0.03147	0.37037	1.81020
Hallella	0.00000	0.00733	0.01028
Hespellia	0.00358	0.01100	0.00000
Howardella	0.00358	0.00000	0.01256
Intestinimonas	0.00215	0.02934	0.10736
Klebsiella	0.00072	0.00733	0.00799
Lachnospiracea_incertae_sedis	0.48558	5.75228	0.22385
Lactobacillus	0.03290	0.10023	0.05596
Leptotrichia	0.00000	0.00000	0.00000
Megamonas	0.00000	0.00000	0.00799
Megasphaera	0.00000	0.15524	0.08337
Methylobacterium	0.00000	0.00000	0.00000
Mitsuokella	0.00000	0.35081	0.32207
Neisseria	0.00286	0.00367	0.01371
Odoribacter	0.00000	0.08923	0.00343
Olsenella	0.00930	0.01222	0.01256
Oribacterium	0.00143	0.00978	0.03426

Genus	W_India_T1_1	W_India_T1_10	W_India_T1_11
**Oscillibacter**	0.00215	0.00367	0.19073
**Parabacteroides**	0.00858	0.80307	0.52079
**Paraprevotella**	0.01645	0.66495	0.00000
**Parasutterella**	0.00000	0.07456	0.00000
**Peptostreptococcus**	0.00072	0.00000	0.00343
**Prevotella**	77.19760	41.06900	46.59350
**Pseudoflavonifractor**	0.00000	0.00000	0.00228
**Pseudomonas**	0.00215	0.00244	0.00000
**Ralstonia**	0.00000	0.00000	0.04226
**Rhizobium**	0.00000	0.00000	0.00000
**Roseburia**	0.85102	8.22017	2.64163
**Rothia**	0.00501	0.00122	0.04111
**Ruminococcus**	0.00143	1.39101	0.12677
**Ruminococcus2**	0.00715	0.08312	0.06624
**Saccharibacteria_genera_incertae_sedis**	0.01001	0.00611	0.00343
**Slackia**	0.00000	0.00000	0.00000
**Solobacterium**	0.00000	0.00000	0.00114
**Streptococcus**	0.87963	0.65150	0.25240
**Streptophyta**	0.01931	0.02567	0.44998
**Succinivibrio**	0.00143	0.00000	20.51980
**Sutterella**	1.41169	1.35190	1.50640
**Turicibacter**	0.01287	0.04034	0.16560
**Veillonella**	0.08725	0.07823	0.08680
**Weissella**	0.00143	0.00000	0.00000

Genus	W_India_T1_12	W_India_T1_13	W_India_T1_14
Acinetobacter	0.00124	0.00285	0.00227
Actinomyces	0.00436	0.00047	0.00417
Akkermansia	0.00000	0.00000	0.00000
Alistipes	0.19537	0.08489	0.00000
Allisonella	0.02302	0.00000	0.00379
Alloprevotella	0.73917	4.71631	7.86255
Anaerostipes	0.00933	0.00617	0.00038
Bacteroides	18.56460	2.59314	0.16564
Barnesiella	0.78522	1.17848	0.00000
Bifidobacterium	3.62929	0.27079	0.13872
Bilophila	0.00684	0.00000	0.00000
Blautia	2.76070	0.46049	0.48402
Butyricicoccus	0.79082	0.07588	0.11939
Butyricimonas	0.04978	0.00000	0.00076
Campylobacter	0.00187	0.00047	0.00038
Catenibacterium	2.20819	0.84415	0.15578
Clostridium_IV	0.08337	0.00047	0.00000
Clostridium_sensu_stricto	0.26630	0.00901	0.56703
Clostridium_XI	0.83997	0.02466	0.23727
Clostridium_XlVa	0.29990	0.18353	0.10575
Clostridium_XlVb	0.09146	0.03177	0.02426
Collinsella	0.54256	0.06402	0.06368
Comamonas	0.00000	0.00000	0.00000
Coprococcus	1.33897	0.19112	0.18686
Dialister	0.74104	0.18258	0.23272
Dorea	0.42123	0.22147	0.42792
Elusimicrobium	0.00000	0.00000	0.00000
Enterobacter	0.00000	0.00000	0.00569
Erysipelotrichaceae_incertae_sedis	0.01618	0.00047	0.00000
Escherichia_Shigella	3.62680	0.13943	0.08490
Faecalibacterium	19.94900	7.13493	7.93456
Flavonifractor	0.00560	0.00190	0.00000
Fusicatenibacter	0.32977	0.02845	0.01061
Fusobacterium	0.01867	0.00000	0.00038
Gemella	0.00311	0.00047	0.00227
Gemmiger	0.61785	0.08347	0.13001
Granulicatella	0.00062	0.00142	0.00493
Haemophilus	0.25635	0.01470	0.11977
Hallella	0.00062	0.00000	0.00644
Hespellia	0.00000	0.00617	0.00227
Howardella	0.01493	0.00000	0.00000
Intestinimonas	0.10017	0.00806	0.00910
Klebsiella	0.00062	0.00047	0.00417
Lachnospiracea_incertae_sedis	0.87544	0.24708	0.12281
Lactobacillus	1.11996	0.09390	0.15540
Leptotrichia	0.00000	0.00190	0.00076
Megamonas	0.00124	0.09390	0.56627
Megasphaera	2.07130	0.00000	0.00114
Methylobacterium	0.00000	0.00000	0.00000
Mitsuokella	1.71976	0.20677	0.00000
Neisseria	0.00000	0.00190	0.00190
Odoribacter	0.22586	0.00000	0.00076
Olsenella	0.13937	0.00000	0.00000
Oribacterium	0.00622	0.00095	0.00152

Genus	W_India_T1_12	W_India_T1_13	W_India_T1_14
**Oscillibacter**	0.08089	0.06165	0.03184
**Parabacteroides**	1.71603	0.71420	0.03563
**Paraprevotella**	0.01120	0.00047	0.00000
**Parasutterella**	0.00996	0.00047	0.00000
**Peptostreptococcus**	0.01556	0.00190	0.00000
**Prevotella**	10.60660	75.12570	75.33440
**Pseudoflavonifractor**	0.00187	0.00000	0.00038
**Pseudomonas**	0.00436	0.00095	0.00227
**Ralstonia**	0.06844	0.00996	0.00114
**Rhizobium**	0.00000	0.00000	0.00000
**Roseburia**	8.46752	4.10075	3.11371
**Rothia**	0.00062	0.00047	0.01213
**Ruminococcus**	0.90841	0.20535	0.24561
**Ruminococcus2**	0.71242	0.01518	0.03298
**Saccharibacteria_genera_incertae_sedis**	0.00809	0.00190	0.00227
**Slackia**	0.00000	0.00000	0.00000
**Solobacterium**	0.00062	0.00000	0.00038
**Streptococcus**	0.17608	0.04600	0.58181
**Streptophyta**	0.02738	0.00000	0.00190
**Succinivibrio**	1.57106	0.00142	0.00265
**Sutterella**	7.31583	0.44626	0.42792
**Turicibacter**	0.28372	0.00711	0.17663
**Veillonella**	0.01493	0.01186	0.02047
**Weissella**	0.00124	0.00000	0.00038

Genus	W_India_T1_15	W_India_T1_16	W_India_T1_17
Acinetobacter	0.03540	0.01045	0.00283
Actinomyces	0.00151	0.00174	0.00071
Akkermansia	0.00000	0.00000	0.00000
Alistipes	0.05573	0.00523	0.33444
Allisonella	0.00000	0.01394	0.00779
Alloprevotella	8.28513	1.22147	0.00567
Anaerostipes	0.03163	0.00000	0.00000
Bacteroides	0.70191	0.04530	9.94112
Barnesiella	0.00000	0.00348	0.00000
Bifidobacterium	0.16945	1.55602	0.08857
Bilophila	0.00000	0.01568	0.02763
Blautia	0.11975	4.30389	0.20548
Butyricicoccus	0.02787	0.17773	0.04889
Butyricimonas	0.00000	0.00000	0.23028
Campylobacter	0.68158	0.00000	0.00071
Catenibacterium	0.00075	0.47395	0.12612
Clostridium_IV	0.00000	0.09758	0.10274
Clostridium_sensu_stricto	0.03690	0.05576	0.05739
Clostridium_XI	0.00527	0.05750	0.02693
Clostridium_XlVa	0.14008	0.21955	0.21753
Clostridium_XlVb	0.02109	0.04705	0.01134
Collinsella	0.02711	0.04008	0.02905
Comamonas	0.00000	0.00000	0.00000
Coprococcus	0.11749	1.19185	0.15305
Dialister	0.46619	3.46750	0.33869
Dorea	0.10167	0.87472	0.11762
Elusimicrobium	0.00000	0.00000	0.00000
Enterobacter	0.00000	0.00000	0.01275
Erysipelotrichaceae_incertae_sedis	0.00000	0.18470	0.30326
Escherichia_Shigella	0.14460	0.00871	0.05456
Faecalibacterium	4.96762	17.00470	8.22923
Flavonifractor	0.00151	0.00000	0.00071
Fusicatenibacter	0.03238	0.03485	0.04889
Fusobacterium	0.31707	0.00174	0.00071
Gemella	0.00151	0.00174	0.00000
Gemmiger	0.03163	0.15334	0.10274
Granulicatella	0.00377	0.00000	0.00000
Haemophilus	0.27866	0.17773	0.00354
Hallella	0.00151	0.03136	0.00000
Hespellia	0.00075	0.03136	0.00425
Howardella	0.00828	0.00000	0.00354
Intestinimonas	0.00828	0.06447	0.00921
Klebsiella	0.00075	0.00000	0.38475
Lachnospiracea_incertae_sedis	0.31029	1.00889	0.12542
Lactobacillus	0.27866	0.24743	0.35428
Leptotrichia	0.00075	0.00174	0.00142
Megamonas	0.74785	0.00000	0.00071
Megasphaera	0.00301	1.14131	0.09778
Methylobacterium	0.00000	0.00000	0.00000
Mitsuokella	0.12803	5.49573	0.00000
Neisseria	0.01356	0.01742	0.00213
Odoribacter	0.00000	0.01220	0.09353
Olsenella	0.00075	0.01917	0.00000
Oribacterium	0.00753	0.00348	0.00213

Genus	W_India_T1_15	W_India_T1_16	W_India_T1_17
**Oscillibacter**	0.01054	0.05924	0.00921
**Parabacteroides**	0.03013	0.03311	0.52859
**Paraprevotella**	0.03464	0.00348	0.14455
**Parasutterella**	0.00000	0.00000	0.21469
**Peptostreptococcus**	0.00075	0.00348	0.00071
**Prevotella**	78.72800	39.88150	75.37470
**Pseudoflavonifractor**	0.00000	0.00523	0.00000
**Pseudomonas**	0.00603	0.00871	0.00425
**Ralstonia**	0.00000	0.00174	0.00000
**Rhizobium**	0.00000	0.00000	0.00000
**Roseburia**	1.32399	7.75571	1.22156
**Rothia**	0.00753	0.00697	0.00071
**Ruminococcus**	0.04142	1.87141	0.41451
**Ruminococcus2**	0.03163	0.11152	0.03401
**Saccharibacteria_genera_incertae_sedis**	0.00904	0.00871	0.00071
**Slackia**	0.00000	0.00000	0.00000
**Solobacterium**	0.00151	0.00000	0.00071
**Streptococcus**	0.11824	0.37289	0.00567
**Streptophyta**	0.00000	0.00000	0.00142
**Succinivibrio**	0.01356	0.00348	0.00425
**Sutterella**	0.90827	0.20910	0.00000
**Turicibacter**	0.00075	0.00174	0.01417
**Veillonella**	0.01582	0.04705	0.00567
**Weissella**	0.00075	0.00174	0.00142

Genus	W_India_T1_18	W_India_T1_19	W_India_T1_2
**Acinetobacter**	0.00062	0.00142	0.00000
**Actinomyces**	0.00154	0.00332	0.00000
**Akkermansia**	0.00000	0.00000	0.00000
**Alistipes**	0.11416	0.27021	0.02880
**Allisonella**	0.00987	0.00142	0.00432
**Alloprevotella**	8.94946	0.00522	3.03375
**Anaerostipes**	0.00000	0.00142	0.00864
**Bacteroides**	4.31908	1.18864	0.27213
**Barnesiella**	0.00000	0.02184	0.02016
**Bifidobacterium**	0.20918	0.28826	0.23901
**Bilophila**	0.00000	0.05129	0.01440
**Blautia**	0.42855	0.41648	0.30669
**Butyricicoccus**	0.02036	0.05841	0.17854
**Butyricimonas**	0.00000	0.21323	0.04320
**Campylobacter**	0.00771	0.00000	0.00288
**Catenibacterium**	0.75435	0.52000	0.21886
**Clostridium_IV**	0.00247	0.27686	0.22750
**Clostridium_sensu_stricto**	0.13575	0.40793	0.64073
**Clostridium_XI**	0.31161	0.25074	0.22462
**Clostridium_XlVa**	0.23973	0.09118	0.08063
**Clostridium_XlVb**	0.02653	0.04606	0.05183
**Collinsella**	0.02561	0.03704	0.00000
**Comamonas**	0.00000	0.00000	0.00000
**Coprococcus**	0.11138	0.08833	1.38801
**Dialister**	0.40880	0.13629	0.68681
**Dorea**	0.18604	0.21702	0.33836
**Elusimicrobium**	0.00000	0.00000	0.00000
**Enterobacter**	0.29033	0.00000	0.00000
**Erysipelotrichaceae_incertae_sedis**	0.00000	0.11207	0.32828
**Escherichia_Shigella**	4.64242	0.00332	0.03024
**Faecalibacterium**	3.80600	8.98773	13.61230
**Flavonifractor**	0.00370	0.13677	0.00288
**Fusicatenibacter**	0.04998	0.06031	0.09215
**Fusobacterium**	0.00802	0.00380	0.00000
**Gemella**	0.00123	0.00095	0.00144
**Gemmiger**	0.28261	0.26356	0.31533
**Granulicatella**	0.00123	0.00142	0.00000
**Haemophilus**	0.19900	0.01425	0.11663
**Hallella**	0.00031	0.00000	0.00720
**Hespellia**	0.00000	0.00475	0.00720
**Howardella**	0.00000	0.00522	0.00000
**Intestinimonas**	0.01697	0.16621	0.18718
**Klebsiella**	1.87616	0.00047	0.00000
**Lachnospiracea_incertae_sedis**	0.07590	0.22415	0.53274
**Lactobacillus**	0.12310	0.06269	0.19438
**Leptotrichia**	0.00062	0.00285	0.00000
**Megamonas**	0.05924	0.00000	0.00144
**Megasphaera**	0.56183	0.22130	0.32253
**Methylobacterium**	0.00000	0.00000	0.00000
**Mitsuokella**	0.40942	0.46587	0.69832
**Neisseria**	0.00524	0.00142	0.01152
**Odoribacter**	0.22214	0.10638	0.01728
**Olsenella**	0.00031	0.03324	0.07775
**Oribacterium**	0.00093	0.00190	0.01152

Genus	W_India_T1_18	W_India_T1_19	W_India_T1_2
**Oscillibacter**	0.02653	0.07646	0.03744
**Parabacteroides**	0.70159	0.64015	0.33116
**Paraprevotella**	0.36591	0.25597	0.00288
**Parasutterella**	0.01018	0.00237	0.00144
**Peptostreptococcus**	0.00309	0.00000	0.00000
**Prevotella**	64.67270	80.96320	48.67390
**Pseudoflavonifractor**	0.00000	0.00142	0.00864
**Pseudomonas**	0.00123	0.00285	0.00144
**Ralstonia**	0.00000	0.00000	0.00000
**Rhizobium**	0.00000	0.00000	0.00000
**Roseburia**	2.84432	0.87949	2.16984
**Rothia**	0.00401	0.00190	0.00144
**Ruminococcus**	0.58929	0.69999	0.91574
**Ruminococcus2**	0.01234	0.07741	0.09503
**Saccharibacteria_genera_incertae_sedis**	0.00339	0.00617	0.01440
**Slackia**	0.00000	0.00000	0.00000
**Solobacterium**	0.00154	0.00047	0.00144
**Streptococcus**	0.04597	0.03277	0.09935
**Streptophyta**	0.00309	0.00095	0.00576
**Succinivibrio**	0.00463	0.00997	21.22040
**Sutterella**	1.30075	0.61830	0.55146
**Turicibacter**	0.12711	0.00000	0.02448
**Veillonella**	0.01759	0.01187	0.02880
**Weissella**	0.02005	0.00047	0.00000

Genus	W_India_T1_20	W_India_T1_21	W_India_T1_22
Acinetobacter	0.00112	0.00239	0.00000
Actinomyces	0.00112	0.00477	0.00000
Akkermansia	0.00000	0.00000	0.00000
Alistipes	0.04191	0.04055	0.20570
Allisonella	0.00187	0.00358	0.00000
Alloprevotella	1.68873	7.71157	0.01286
Anaerostipes	0.00037	0.03339	0.00000
Bacteroides	0.17063	0.33155	2.32953
Barnesiella	0.00037	0.03697	0.00000
Bifidobacterium	0.16165	0.25522	0.20998
Bilophila	0.00449	0.00358	0.00000
Blautia	0.11114	0.23137	0.33769
Butyricicoccus	0.07671	0.10734	0.02828
Butyricimonas	0.00449	0.00119	0.00000
Campylobacter	0.00000	0.00119	0.00171
Catenibacterium	0.11001	0.13358	0.00086
Clostridium_IV	0.02732	0.01789	0.52367
Clostridium_sensu_stricto	0.04341	0.43054	0.03685
Clostridium_XI	0.05014	0.23256	0.01457
Clostridium_XlVa	0.05014	0.15266	0.13113
Clostridium_XlVb	0.03068	0.02027	0.01114
Collinsella	0.02058	0.04413	0.01114
Comamonas	0.00000	0.00000	0.00000
Coprococcus	0.51901	0.11449	0.14742
Dialister	0.49730	0.53072	0.20484
Dorea	0.13508	0.24449	0.19199
Elusimicrobium	0.00000	0.00000	0.00000
Enterobacter	0.00000	0.00716	0.00000
Erysipelotrichaceae_incertae_sedis	0.06025	0.01670	0.22198
Escherichia_Shigella	0.55231	2.42343	0.00343
Faecalibacterium	2.76641	6.61077	9.22212
Flavonifractor	0.00000	0.00000	0.00086
Fusicatenibacter	0.01908	0.01312	0.05142
Fusobacterium	0.00037	0.00119	0.00171
Gemella	0.00037	0.00239	0.00000
Gemmiger	0.05688	0.16816	0.19284
Granulicatella	0.00112	0.00477	0.00086
Haemophilus	0.00674	0.15266	0.00171
Hallella	0.00449	0.19321	0.00000
Hespellia	0.00262	0.00119	0.00171
Howardella	0.00337	0.00358	0.00000
Intestinimonas	0.01871	0.01550	0.02571
Klebsiella	0.01272	0.78595	0.00000
Lachnospiracea_incertae_sedis	0.16502	0.16697	0.38654
Lactobacillus	0.02470	0.20275	0.05485
Leptotrichia	0.00037	0.00119	0.00171
Megamonas	0.00037	0.37330	0.00000
Megasphaera	0.00037	0.05725	0.11228
Methylobacterium	0.00000	0.00000	0.00000
Mitsuokella	0.01048	0.08945	0.45682
Neisseria	0.00374	0.01312	0.00171
Odoribacter	0.12199	0.00596	0.03000
Olsenella	0.00187	0.00000	0.00000
Oribacterium	0.00486	0.00239	0.00429

Genus	W_India_T1_20	W_India_T1_21	W_India_T1_22
**Oscillibacter**	0.01609	0.04890	0.01286
**Parabacteroides**	0.14781	0.09303	0.23398
**Paraprevotella**	0.02208	0.01550	0.00000
**Parasutterella**	0.00000	0.00119	0.01457
**Peptostreptococcus**	0.00000	0.00239	0.00086
**Prevotella**	50.37570	72.95460	81.21640
**Pseudoflavonifractor**	0.00075	0.00000	0.00000
**Pseudomonas**	0.00374	0.00000	0.00514
**Ralstonia**	0.00112	0.01550	0.00000
**Rhizobium**	0.00000	0.00000	0.00000
**Roseburia**	1.19180	2.99948	2.11783
**Rothia**	0.00075	0.01431	0.00086
**Ruminococcus**	0.08943	0.18009	0.29055
**Ruminococcus2**	0.11413	0.02624	0.01543
**Saccharibacteria_genera_incertae_sedis**	0.00112	0.00358	0.00086
**Slackia**	0.00000	0.00000	0.00000
**Solobacterium**	0.00037	0.00358	0.00000
**Streptococcus**	0.00674	0.45439	0.07542
**Streptophyta**	0.00000	0.00835	0.01800
**Succinivibrio**	40.17710	0.25165	0.01114
**Sutterella**	0.14182	0.46871	0.30255
**Turicibacter**	0.08083	0.34944	0.00086
**Veillonella**	0.00374	0.02505	0.00000
**Weissella**	0.00000	0.00000	0.00000

Genus	W_India_T1_23	W_India_T1_24	W_India_T1_25
Acinetobacter	0.00161	0.01971	0.00000
Actinomyces	0.00535	0.00104	0.00376
Akkermansia	0.00000	0.00000	0.00000
Alistipes	0.04123	0.00104	0.00000
Allisonella	0.01606	0.00000	0.00000
Alloprevotella	9.97810	4.78201	3.95389
Anaerostipes	0.07495	0.00207	0.00251
Bacteroides	0.39994	0.03319	0.04014
Barnesiella	0.00696	0.00104	0.00000
Bifidobacterium	1.18536	0.10062	0.18942
Bilophila	0.00268	0.00104	0.00251
Blautia	0.39673	0.10269	0.25841
Butyricicoccus	0.25163	0.01867	0.04390
Butyricimonas	0.00054	0.00104	0.00502
Campylobacter	0.00375	0.00000	0.00627
Catenibacterium	0.12100	0.29356	0.60462
Clostridium_IV	0.00696	0.00830	0.03512
Clostridium_sensu_stricto	0.68155	0.09025	0.46664
Clostridium_XI	1.27423	0.18672	0.61090
Clostridium_XlVa	0.28108	0.09025	0.06147
Clostridium_XlVb	0.03908	0.01763	0.11415
Collinsella	0.48721	0.00104	0.00000
Comamonas	0.00000	0.00000	0.00000
Coprococcus	0.26823	0.10477	0.46915
Dialister	0.99208	0.88068	0.77397
Dorea	0.41172	0.08921	0.23959
Elusimicrobium	0.00000	0.00000	0.00000
Enterobacter	0.00268	0.00104	0.00000
Erysipelotrichaceae_incertae_sedis	0.16008	0.02697	0.24210
Escherichia_Shigella	0.20024	0.00622	0.15429
Faecalibacterium	11.15810	1.54352	12.23170
Flavonifractor	0.00214	0.00000	0.00000
Fusicatenibacter	0.02623	0.00830	0.17060
Fusobacterium	0.00482	0.00104	0.00125
Gemella	0.00803	0.00000	0.00376
Gemmiger	0.29928	0.01867	0.09910
Granulicatella	0.00696	0.00000	0.00376
Haemophilus	2.36911	0.17012	0.36127
Hallella	0.18257	0.00104	0.04516
Hespellia	0.00321	0.00000	0.00125
Howardella	0.02034	0.00622	0.03638
Intestinimonas	0.03748	0.01556	0.04390
Klebsiella	0.09905	0.00104	0.00251
Lachnospiracea_incertae_sedis	0.32713	0.14004	0.45410
Lactobacillus	0.88286	0.01867	0.23834
Leptotrichia	0.00054	0.00000	0.00251
Megamonas	0.29018	0.00000	0.00000
Megasphaera	0.33409	0.00000	0.05645
Methylobacterium	0.00000	0.00000	0.00000
Mitsuokella	0.44009	0.06120	0.12168
Neisseria	0.03212	0.00000	0.02760
Odoribacter	0.01606	0.00622	0.00000
Olsenella	0.00482	0.00000	0.00000
Oribacterium	0.04390	0.00622	0.00502

Genus	W_India_T1_23	W_India_T1_24	W_India_T1_25
Oscillibacter	0.09423	0.01660	0.02760
Parabacteroides	0.11029	0.00000	0.12419
Paraprevotella	0.02677	0.06743	0.01882
Parasutterella	0.00535	0.00000	0.00000
Peptostreptococcus	0.00482	0.00000	0.00000
Prevotella	56.77400	88.62380	58.37000
Pseudoflavonifractor	0.00161	0.00000	0.00502
Pseudomonas	0.00107	0.00000	0.00000
Ralstonia	0.02409	0.00000	0.00000
Rhizobium	0.00000	0.00000	0.00000
Roseburia	6.11739	1.92836	5.50057
Rothia	0.01820	0.00000	0.01129
Ruminococcus	0.24735	0.01037	1.03489
Ruminococcus2	0.09476	0.07469	0.14802
Saccharibacteria_genera_incertae_sedis	0.02463	0.00000	0.00376
Slackia	0.00000	0.00000	0.00000
Solobacterium	0.00161	0.00000	0.00125
Streptococcus	0.58518	0.03527	0.22454
Streptophyta	0.00428	0.00000	0.00376
Succinivibrio	0.24467	0.00207	11.89180
Sutterella	0.33355	0.31119	0.17060
Turicibacter	1.16341	0.00000	0.03763
Veillonella	0.06318	0.00311	0.04014
Weissella	0.00107	0.00000	0.00000

Genus	W_India_T1_26	W_India_T1_27	W_India_T1_28
Acinetobacter	0.00347	0.00132	0.02079
Actinomyces	0.00347	0.00132	0.00347
Akkermansia	0.00000	0.00000	0.00000
Alistipes	0.11987	0.11603	1.28444
Allisonella	0.00000	0.00000	0.00000
Alloprevotella	4.96971	7.94304	0.02195
Anaerostipes	0.00043	0.00000	0.00000
Bacteroides	0.52594	0.49314	3.64308
Barnesiella	0.00000	0.01319	1.03725
Bifidobacterium	0.70444	0.09230	0.82356
Bilophila	0.00347	0.00659	0.02541
Blautia	0.29533	0.17273	0.59833
Butyricicoccus	0.05472	0.02769	0.14438
Butyricimonas	0.20021	0.06593	0.02310
Campylobacter	0.00087	0.00000	0.00578
Catenibacterium	0.00043	0.49314	2.67976
Clostridium_IV	0.04300	0.19383	0.36731
Clostridium_sensu_stricto	0.62583	0.27954	0.25527
Clostridium_XI	1.51571	0.14900	0.20907
Clostridium_XlVa	0.15374	0.03560	0.11320
Clostridium_XlVb	0.02215	0.01582	0.11089
Collinsella	0.01998	0.02373	0.00116
Comamonas	0.00000	0.00000	0.00000
Coprococcus	0.71182	0.05406	0.51516
Dialister	2.10115	0.59467	0.69420
Dorea	0.28143	0.08043	0.40196
Elusimicrobium	0.00000	0.00000	0.00000
Enterobacter	0.00043	0.00264	0.00116
Erysipelotrichaceae_incertae_sedis	0.00000	0.00264	0.70575
Escherichia_Shigella	0.12986	0.22548	0.15478
Faecalibacterium	21.50490	6.99499	9.11580
Flavonifractor	0.00000	0.00000	0.04620
Fusicatenibacter	0.00782	0.02242	0.14207
Fusobacterium	0.00087	0.00000	0.01502
Gemella	0.00217	0.00000	0.00347
Gemmiger	0.30054	0.08307	0.46087
Granulicatella	0.00217	0.00000	0.00462
Haemophilus	0.48425	0.12790	0.07277
Hallella	0.00478	0.02505	0.00231
Hespellia	0.00695	0.00132	0.00809
Howardella	0.02649	0.01582	0.00000
Intestinimonas	0.01998	0.01846	0.03812
Klebsiella	0.00695	0.14504	0.00462
Lachnospiracea_incertae_sedis	0.24234	0.18196	0.70113
Lactobacillus	0.06949	0.05670	0.27837
Leptotrichia	0.00391	0.00000	0.00231
Megamonas	0.00000	0.00000	0.00116
Megasphaera	0.15157	0.10944	0.09356
Methylobacterium	0.00000	0.00000	0.00000
Mitsuokella	0.51769	0.05670	0.00000
Neisseria	0.00217	0.00132	0.03696
Odoribacter	0.00000	0.14900	0.33035
Olsenella	0.01173	0.00132	0.00116
Oribacterium	0.00174	0.17669	0.00809

Genus	W_India_T1_26	W_India_T1_27	W_India_T1_28
**Oscillibacter**	0.03865	0.03428	0.11089
**Parabacteroides**	0.40911	0.17801	2.18654
**Paraprevotella**	0.00000	0.13449	0.13745
**Parasutterella**	0.00955	0.01319	0.00000
**Peptostreptococcus**	0.00217	0.00000	0.00000
**Prevotella**	55.21790	77.92060	28.79580
**Pseudoflavonifractor**	0.00130	0.01055	0.00231
**Pseudomonas**	0.00000	0.00000	0.01040
**Ralstonia**	0.00000	0.00132	0.00809
**Rhizobium**	0.00000	0.00000	0.00000
**Roseburia**	4.86070	1.18935	1.52584
**Rothia**	0.00955	0.00132	0.00578
**Ruminococcus**	1.19042	0.47468	3.31967
**Ruminococcus2**	0.11205	0.02505	0.22639
**Saccharibacteria_genera_incertae_sedis**	0.00869	0.00264	0.01386
**Slackia**	0.00000	0.00000	0.00000
**Solobacterium**	0.00304	0.00000	0.00116
**Streptococcus**	1.25339	0.01187	0.18019
**Streptophyta**	0.01042	0.00000	0.00462
**Succinivibrio**	0.01042	0.00000	37.10080
**Sutterella**	0.46036	0.62236	0.07623
**Turicibacter**	0.27578	0.03296	0.00231
**Veillonella**	0.10597	0.01055	0.04505
**Weissella**	0.00521	0.00000	0.00000

Genus	W_India_T1_29	W_India_T1_3	W_India_T1_30
**Acinetobacter**	0.00029	0.00153	0.00036
**Actinomyces**	0.00174	0.01381	0.00181
**Akkermansia**	0.00000	0.00000	0.00000
**Alistipes**	0.00869	0.00307	0.00000
**Allisonella**	0.00695	0.00153	0.00000
**Alloprevotella**	4.94463	0.10130	0.00651
**Anaerostipes**	0.02287	0.67227	0.00326
**Bacteroides**	0.14389	0.94395	62.13510
**Barnesiella**	0.00000	0.00153	0.00000
**Bifidobacterium**	0.22322	13.38260	0.38242
**Bilophila**	0.00116	0.00000	0.03365
**Blautia**	0.18847	0.69530	0.97143
**Butyricicoccus**	0.03358	1.31385	0.21563
**Butyricimonas**	0.00145	0.00000	0.00000
**Campylobacter**	0.00000	0.00614	0.01628
**Catenibacterium**	0.00000	0.01228	0.00036
**Clostridium_IV**	0.01361	0.00000	0.03220
**Clostridium_sensu_stricto**	0.26491	0.12433	0.09226
**Clostridium_XI**	0.18500	0.08749	0.01737
**Clostridium_XlVa**	0.06775	0.28549	0.16100
**Clostridium_XlVb**	0.00608	0.00000	0.63424
**Collinsella**	0.00897	0.00153	0.00036
**Comamonas**	0.00000	0.00000	0.00000
**Coprococcus**	0.19687	0.01228	0.14834
**Dialister**	0.90792	0.36837	0.71021
**Dorea**	0.14418	0.77204	0.27280
**Elusimicrobium**	0.00000	0.00000	0.00000
**Enterobacter**	0.00000	0.00153	0.00000
**Erysipelotrichaceae_incertae_sedis**	0.00000	0.01074	0.00072
**Escherichia_Shigella**	0.00608	27.66150	0.18741
**Faecalibacterium**	8.80242	19.68780	20.74990
**Flavonifractor**	0.00058	0.00460	0.01773
**Fusicatenibacter**	0.03561	0.58172	0.44429
**Fusobacterium**	0.00000	0.01688	0.01592
**Gemella**	0.00000	0.00460	0.00109
**Gemmiger**	0.18558	0.18265	0.23191
**Granulicatella**	0.00029	0.00460	0.00362
**Haemophilus**	0.00058	1.53487	0.07670
**Hallella**	0.00492	0.00000	0.00036
**Hespellia**	0.00347	0.00000	0.00470
**Howardella**	0.00405	0.00000	0.00000
**Intestinimonas**	0.01940	0.00000	0.00036
**Klebsiella**	0.00000	0.05219	0.00796
**Lachnospiracea_incertae_sedis**	0.14794	0.46814	0.94683
**Lactobacillus**	0.35466	0.82269	0.00362
**Leptotrichia**	0.00000	0.00000	0.00072
**Megamonas**	0.00000	0.00000	0.47830
**Megasphaera**	0.00000	0.02763	0.00434
**Methylobacterium**	0.00000	0.00000	0.00000
**Mitsuokella**	0.30747	0.01995	0.00000
**Neisseria**	0.00000	0.05065	0.00109
**Odoribacter**	0.01940	0.00000	0.00036
**Olsenella**	0.09207	0.00921	0.00000
**Oribacterium**	0.00174	0.00614	0.00579

Genus	W_India_T1_29	W_India_T1_3	W_India_T1_30
**Oscillibacter**	0.02635	0.00000	0.03365
**Parabacteroides**	0.10625	0.02916	1.76233
**Paraprevotella**	0.00000	0.00153	0.00000
**Parasutterella**	0.01100	0.87488	0.00000
**Peptostreptococcus**	0.00000	0.01228	0.00217
**Prevotella**	80.49620	1.78659	0.06802
**Pseudoflavonifractor**	0.00203	0.00000	0.00000
**Pseudomonas**	0.00000	0.23330	0.00036
**Ralstonia**	0.00087	0.39293	0.00398
**Rhizobium**	0.00000	0.00000	0.00000
**Roseburia**	1.04283	4.41276	7.80257
**Rothia**	0.00000	0.00921	0.00253
**Ruminococcus**	0.21656	0.00921	0.75218
**Ruminococcus2**	0.08049	0.58632	0.19863
**Saccharibacteria_genera_incertae_sedis**	0.00058	0.01995	0.02858
**Slackia**	0.00000	0.00000	0.00000
**Solobacterium**	0.00058	0.00614	0.00000
**Streptococcus**	0.00087	16.79150	0.13097
**Streptophyta**	0.00434	0.07060	0.00072
**Succinivibrio**	0.00318	0.08749	0.00724
**Sutterella**	0.63172	0.10744	0.00072
**Turicibacter**	0.00608	0.30544	0.00181
**Veillonella**	0.00000	2.72593	0.09769
**Weissella**	0.00000	0.03837	0.00253

Genus	W_India_T1_31	W_India_T1_32	W_India_T1_33
Acinetobacter	0.00144	0.00198	0.00000
Actinomyces	0.00289	0.00791	0.00872
Akkermansia	0.00000	0.00000	0.00000
Alistipes	0.01878	0.01285	0.91012
Allisonella	0.00144	0.00395	0.00000
Alloprevotella	0.01589	10.48370	1.28847
Anaerostipes	0.00867	0.04941	0.00349
Bacteroides	3.58531	0.42101	2.76523
Barnesiella	0.01589	0.00296	2.38689
Bifidobacterium	0.67893	0.34293	0.33650
Bilophila	0.00433	0.00099	0.03138
Blautia	0.59803	0.35578	0.49168
Butyricicoccus	0.07367	0.14132	0.09066
Butyricimonas	0.03034	0.00198	0.08020
Campylobacter	0.00000	0.00296	0.00000
Catenibacterium	0.00578	0.20952	0.20051
Clostridium_IV	0.00000	0.03657	0.92581
Clostridium_sensu_stricto	0.12278	0.57814	0.59629
Clostridium_XI	0.27013	0.35973	0.46901
Clostridium_XlVa	0.16612	0.19963	0.10984
Clostridium_XlVb	0.06645	0.05534	0.04010
Collinsella	0.13723	0.05040	0.02615
Comamonas	0.00000	0.00000	0.00000
Coprococcus	0.73237	0.19370	0.62593
Dialister	0.40013	0.55838	2.38863
Dorea	0.49981	0.36270	0.27025
Elusimicrobium	0.00000	0.00000	0.00000
Enterobacter	0.00867	0.00791	0.00000
Erysipelotrichaceae_incertae_sedis	0.10545	0.01977	0.07846
Escherichia_Shigella	0.32357	0.16504	0.10287
Faecalibacterium	5.08328	7.66213	21.25360
Flavonifractor	0.00289	0.00593	0.00000
Fusicatenibacter	0.17334	0.01285	0.05928
Fusobacterium	0.00144	0.00198	0.00523
Gemella	0.00433	0.00296	0.00000
Gemmiger	0.18345	0.23521	0.13425
Granulicatella	0.00289	0.00494	0.00000
Haemophilus	0.07656	0.21050	0.24584
Hallella	0.00000	0.24015	0.00000
Hespellia	0.00289	0.00198	0.00872
Howardella	0.00000	0.00395	0.06102
Intestinimonas	0.04334	0.01384	0.33999
Klebsiella	0.00433	0.03953	0.00000
Lachnospiracea_incertae_sedis	0.66881	0.26684	0.27722
Lactobacillus	0.45214	0.30439	0.01395
Leptotrichia	0.00000	0.00000	0.00174
Megamonas	0.00144	0.53466	0.00349
Megasphaera	0.00144	0.04941	0.25281
Methylobacterium	0.00000	0.00000	0.00000
Mitsuokella	0.00578	0.09290	0.84561
Neisseria	0.00433	0.00889	0.00872
Odoribacter	0.00722	0.01680	0.14471
Olsenella	0.00289	0.00000	0.01046
Oribacterium	0.00433	0.00692	0.01395

Genus	W_India_T1_31	W_India_T1_32	W_India_T1_33
**Oscillibacter**	0.02167	0.05633	0.32081
**Parabacteroides**	0.12856	0.10970	1.04089
**Paraprevotella**	0.00144	0.03953	0.31558
**Parasutterella**	0.00144	0.00000	0.02441
**Peptostreptococcus**	0.00289	0.00198	0.00000
**Prevotella**	77.48280	68.12010	55.52260
**Pseudoflavonifractor**	0.00000	0.00692	0.00349
**Pseudomonas**	0.00000	0.00395	0.00000
**Ralstonia**	0.00000	0.02273	0.00000
**Rhizobium**	0.00000	0.00000	0.00000
**Roseburia**	5.33751	4.04601	2.18987
**Rothia**	0.00578	0.01384	0.00872
**Ruminococcus**	0.08089	0.26782	1.13852
**Ruminococcus2**	0.08667	0.02767	0.06102
**Saccharibacteria_genera_incertae_sedis**	0.00578	0.00791	0.00523
**Slackia**	0.00000	0.00000	0.00000
**Solobacterium**	0.00000	0.00099	0.00174
**Streptococcus**	0.05923	0.63843	0.05754
**Streptophyta**	0.00000	0.03064	0.00349
**Succinivibrio**	0.00144	1.08118	0.00349
**Sutterella**	0.62403	0.54553	1.11063
**Turicibacter**	1.50664	0.48129	0.08892
**Veillonella**	0.02745	0.02767	0.00697
**Weissella**	0.00000	0.00000	0.00000

Genus	W_India_T1_34	W_India_T1_35	W_India_T1_36
Acinetobacter	0.00161	0.00246	0.00000
Actinomyces	0.00081	0.00123	0.00191
Akkermansia	0.00000	0.00000	0.00000
Alistipes	0.04279	0.00000	0.08517
Allisonella	0.00807	0.00000	0.00763
Alloprevotella	6.47870	0.00614	5.67911
Anaerostipes	0.00081	0.00368	0.01144
Bacteroides	0.20912	0.01350	0.32988
Barnesiella	0.05813	0.00000	0.00127
Bifidobacterium	0.23900	2.80281	0.41950
Bilophila	0.00081	0.00000	0.02352
Blautia	0.18651	0.56351	0.13348
Butyricicoccus	0.05652	0.07121	0.06483
Butyricimonas	0.00081	0.00000	0.04068
Campylobacter	0.00000	0.00123	0.00064
Catenibacterium	0.18490	3.38719	0.01525
Clostridium_IV	0.02180	0.00000	0.02924
Clostridium_sensu_stricto	0.18974	0.12891	0.48560
Clostridium_XI	0.08074	0.73293	0.13284
Clostridium_XlVa	0.08316	0.19766	0.05466
Clostridium_XlVb	0.02099	0.01842	0.02225
Collinsella	0.01534	0.10804	0.05593
Comamonas	0.00000	0.00000	0.00000
Coprococcus	0.11062	0.09208	0.38327
Dialister	2.27933	0.68996	0.77099
Dorea	0.09931	0.18047	0.10487
Elusimicrobium	0.00000	0.00000	0.00000
Enterobacter	0.00484	0.00246	0.00000
Erysipelotrichaceae_incertae_sedis	0.03795	0.00000	0.00254
Escherichia_Shigella	1.07144	0.86184	0.03305
Faecalibacterium	18.37680	4.69222	9.77048
Flavonifractor	0.00000	0.00000	0.00318
Fusicatenibacter	0.02664	0.18661	0.03114
Fusobacterium	0.00000	0.00123	0.00445
Gemella	0.00081	0.00000	0.00127
Gemmiger	0.08074	0.16574	0.16780
Granulicatella	0.00081	0.00614	0.00064
Haemophilus	0.07428	0.77713	0.12331
Hallella	0.00000	0.00000	0.03941
Hespellia	0.00000	0.00000	0.00000
Howardella	0.00161	0.00000	0.00000
Intestinimonas	0.01776	0.00614	0.01017
Klebsiella	0.05571	0.00000	0.02161
Lachnospiracea_incertae_sedis	0.15422	0.76976	0.17352
Lactobacillus	0.05410	0.14487	0.22628
Leptotrichia	0.00081	0.00123	0.00000
Megamonas	0.00161	3.46576	0.01843
Megasphaera	0.06702	0.00000	0.21229
Methylobacterium	0.00000	0.00000	0.00000
Mitsuokella	0.00000	0.00000	0.02225
Neisseria	0.00081	0.00491	0.00572
Odoribacter	0.01615	0.00000	0.00127
Olsenella	0.00484	0.00123	0.00318
Oribacterium	0.00242	0.00368	0.01081

Genus	W_India_T1_34	W_India_T1_35	W_India_T1_36
Oscillibacter	0.02180	0.00368	0.03750
Parabacteroides	0.15260	0.00000	0.32162
Paraprevotella	0.01938	0.00000	0.08898
Parasutterella	0.00242	0.00000	0.00191
Peptostreptococcus	0.00161	0.00737	0.00064
Prevotella	66.29530	73.20450	72.01700
Pseudoflavonifractor	0.00000	0.00000	0.00127
Pseudomonas	0.00000	0.00000	0.00191
Ralstonia	0.00000	0.00000	0.00064
Rhizobium	0.00000	0.00000	0.00000
Roseburia	2.34958	3.67569	0.86124
Rothia	0.00323	0.00859	0.00890
Ruminococcus	0.13565	0.00000	0.02860
Ruminococcus2	0.01776	0.05525	0.00381
Saccharibacteria_genera_incertae_sedis	0.00161	0.01228	0.00763
Slackia	0.00000	0.00000	0.00000
Solobacterium	0.00000	0.00246	0.00000
Streptococcus	0.00807	1.03985	0.14238
Streptophyta	0.00000	0.00614	0.10487
Succinivibrio	0.00161	0.00368	0.09598
Sutterella	0.45861	1.46954	3.46403
Turicibacter	0.02907	0.16451	0.40424
Veillonella	0.00323	0.21239	0.01271
Weissella	0.00323	0.00246	0.00127

Genus	W_India_T1_37	W_India_T1_38	W_India_T1_39
**Acinetobacter**	0.00106	0.00374	0.00000
**Actinomyces**	0.00422	0.00374	0.00116
**Akkermansia**	0.00000	0.00000	0.00000
**Alistipes**	0.00739	0.43601	0.57388
**Allisonella**	0.01478	0.00374	0.00233
**Alloprevotella**	6.52692	3.04646	1.49000
**Anaerostipes**	0.00000	0.10666	0.06053
**Bacteroides**	0.60372	0.78220	0.61812
**Barnesiella**	0.00000	0.58572	0.40393
**Bifidobacterium**	0.10555	0.20023	0.38181
**Bilophila**	0.00000	0.02246	0.05005
**Blautia**	0.43801	0.31812	0.52034
**Butyricicoccus**	0.08338	0.07111	0.11524
**Butyricimonas**	0.00000	0.20397	0.15249
**Campylobacter**	0.00000	0.00187	0.00466
**Catenibacterium**	0.17309	0.18339	0.15482
**Clostridium_IV**	0.00317	1.58124	1.85784
**Clostridium_sensu_stricto**	0.33247	1.11716	0.71823
**Clostridium_XI**	0.40213	0.46969	0.31779
**Clostridium_XlVa**	0.14038	0.08234	0.11641
**Clostridium_XlVb**	0.01900	0.04865	0.11524
**Collinsella**	0.03694	0.15158	0.21302
**Comamonas**	0.00000	0.00000	0.00000
**Coprococcus**	0.16993	1.25564	1.73213
**Dialister**	0.45701	0.27695	0.22816
**Dorea**	0.40424	0.25450	0.36086
**Elusimicrobium**	0.00000	0.00000	0.00000
**Enterobacter**	0.03694	0.00000	0.00000
**Erysipelotrichaceae_incertae_sedis**	0.00000	0.31999	0.26541
**Escherichia_Shigella**	0.46334	0.03555	0.04540
**Faecalibacterium**	9.58352	25.99410	37.32100
**Flavonifractor**	0.00000	0.00000	0.00116
**Fusicatenibacter**	0.03166	0.01871	0.03259
**Fusobacterium**	0.00000	0.00749	0.03609
**Gemella**	0.00000	0.00187	0.00000
**Gemmiger**	0.08233	0.17403	0.29451
**Granulicatella**	0.00528	0.00187	0.00233
**Haemophilus**	0.13826	0.03930	0.04423
**Hallella**	0.00211	0.04117	0.08032
**Hespellia**	0.00422	0.00561	0.00931
**Howardella**	0.00000	0.03181	0.03841
**Intestinimonas**	0.01055	0.16467	0.22816
**Klebsiella**	0.13826	0.00187	0.00000
**Lachnospiracea_incertae_sedis**	0.20265	0.61191	0.57505
**Lactobacillus**	0.19209	0.40981	0.14085
**Leptotrichia**	0.00000	0.00000	0.00000
**Megamonas**	0.42429	0.00000	0.00000
**Megasphaera**	0.04644	0.22081	0.35038
**Methylobacterium**	0.00000	0.00000	0.00000
**Mitsuokella**	0.03061	0.47905	0.77061
**Neisseria**	0.00000	0.00561	0.00116
**Odoribacter**	0.00950	0.18339	0.09080
**Olsenella**	0.00000	0.00187	0.01630
**Oribacterium**	0.00739	0.00561	0.00466

Genus	W_India_T1_37	W_India_T1_38	W_India_T1_39
**Oscillibacter**	0.02322	0.13286	0.22466
**Parabacteroides**	0.12877	1.58124	1.18036
**Paraprevotella**	0.02744	0.00187	0.00000
**Parasutterella**	0.00000	0.00749	0.00116
**Peptostreptococcus**	0.00211	0.01123	0.00116
**Prevotella**	73.77620	34.61890	25.01690
**Pseudoflavonifractor**	0.00000	0.01123	0.00815
**Pseudomonas**	0.00000	0.00000	0.00000
**Ralstonia**	0.00000	0.00000	0.00116
**Rhizobium**	0.00000	0.00000	0.00000
**Roseburia**	2.64919	5.96755	6.83189
**Rothia**	0.01583	0.04117	0.01048
**Ruminococcus**	0.43907	0.88138	0.87654
**Ruminococcus2**	0.04961	0.02433	0.05122
**Saccharibacteria_genera_incertae_sedis**	0.00422	0.00749	0.00349
**Slackia**	0.00000	0.00000	0.00000
**Solobacterium**	0.00106	0.00000	0.01164
**Streptococcus**	0.35674	0.14222	0.09080
**Streptophyta**	0.00106	0.17216	0.01164
**Succinivibrio**	0.00211	14.40900	13.72310
**Sutterella**	1.01746	0.23017	0.18043
**Turicibacter**	0.08127	0.09731	0.07217
**Veillonella**	0.03061	0.04678	0.02910
**Weissella**	0.00633	0.00000	0.00000

Genus	W_India_T1_4	W_India_T1_40	W_India_T1_41
**Acinetobacter**	0.00291	0.00125	0.00000
**Actinomyces**	0.00291	0.00031	0.00000
**Akkermansia**	0.00000	0.00000	0.00000
**Alistipes**	0.00970	0.01904	0.00670
**Allisonella**	0.00000	0.00562	0.03605
**Alloprevotella**	1.68092	6.52368	0.20773
**Anaerostipes**	0.00776	0.00031	0.00000
**Bacteroides**	0.43163	0.02871	0.01664
**Barnesiella**	0.00097	0.00000	0.00000
**Bifidobacterium**	0.32299	0.11454	0.21028
**Bilophila**	0.01164	0.00156	0.00000
**Blautia**	0.25704	0.10643	0.00393
**Butyricicoccus**	0.02813	0.03714	0.00716
**Butyricimonas**	0.10573	0.00593	0.00000
**Campylobacter**	0.00000	1.13826	0.00069
**Catenibacterium**	0.00097	0.49344	0.00647
**Clostridium_IV**	0.03395	0.00312	0.00578
**Clostridium_sensu_stricto**	0.01746	0.49750	0.00046
**Clostridium_XI**	0.09021	0.46691	0.00000
**Clostridium_XlVa**	0.09021	0.19070	0.00092
**Clostridium_XlVb**	0.00873	0.01717	0.00347
**Collinsella**	0.21824	0.04057	0.00023
**Comamonas**	0.00000	0.00000	0.00000
**Coprococcus**	0.18138	0.32709	0.11230
**Dialister**	0.17362	0.48221	0.02357
**Dorea**	0.21824	0.22878	0.01548
**Elusimicrobium**	0.00000	0.00000	0.00000
**Enterobacter**	0.00000	0.00250	0.01618
**Erysipelotrichaceae_incertae_sedis**	0.00000	0.03870	0.00000
**Escherichia_Shigella**	0.05238	0.26748	0.05915
**Faecalibacterium**	2.01265	4.93661	0.45082
**Flavonifractor**	0.00097	0.00000	0.00023
**Fusicatenibacter**	0.02037	0.01186	0.00300
**Fusobacterium**	0.00582	0.00062	0.00023
**Gemella**	0.00194	0.00125	0.00046
**Gemmiger**	0.28614	0.12703	0.00924
**Granulicatella**	0.00097	0.00125	0.00092
**Haemophilus**	0.00970	0.32334	0.17793
**Hallella**	0.00000	0.01592	0.00000
**Hespellia**	0.00485	0.00562	0.00000
**Howardella**	0.00097	0.00250	0.00000
**Intestinimonas**	0.01358	0.02403	0.00208
**Klebsiella**	0.00000	0.05743	0.37434
**Lachnospiracea_incertae_sedis**	0.08051	0.83551	0.02542
**Lactobacillus**	0.15616	0.14669	0.27359
**Leptotrichia**	0.00194	0.00125	0.00023
**Megamonas**	0.10767	0.00000	2.14596
**Megasphaera**	0.07469	0.03340	0.07672
**Methylobacterium**	0.00000	0.00000	0.00000
**Mitsuokella**	0.08342	0.11454	0.01918
**Neisseria**	0.01358	0.00437	0.00139
**Odoribacter**	0.03104	0.00000	0.00647
**Olsenella**	0.00097	0.00094	0.00000
**Oribacterium**	0.00097	0.05524	0.00046

Genus	W_India_T1_4	W_India_T1_40	W_India_T1_41
**Oscillibacter**	0.00485	0.03277	0.00231
**Parabacteroides**	0.13967	0.03496	0.01618
**Paraprevotella**	0.05626	0.00437	0.00000
**Parasutterella**	0.00776	0.00343	0.00462
**Peptostreptococcus**	0.00000	0.00000	0.00000
**Prevotella**	89.49830	51.25650	91.58250
**Pseudoflavonifractor**	0.00000	0.00406	0.00023
**Pseudomonas**	0.01164	0.00000	0.00000
**Ralstonia**	0.00582	0.00218	0.00046
**Rhizobium**	0.00000	0.00000	0.00000
**Roseburia**	1.92535	3.47813	0.12547
**Rothia**	0.00388	0.00624	0.00739
**Ruminococcus**	0.12803	0.12609	0.00693
**Ruminococcus2**	0.00970	0.02216	0.00462
**Saccharibacteria_genera_incertae_sedis**	0.00388	0.00843	0.00208
**Slackia**	0.00000	0.00000	0.00000
**Solobacterium**	0.00000	0.00000	0.00000
**Streptococcus**	0.02328	0.26248	2.64114
**Streptophyta**	0.00000	0.02247	0.01040
**Succinivibrio**	0.00679	24.84880	0.00139
**Sutterella**	1.22602	0.47628	0.42124
**Turicibacter**	0.00291	0.47066	0.00023
**Veillonella**	0.00970	0.02622	0.04390
**Weissella**	0.00000	0.33396	0.80898

Genus	W_India_T1_42	W_India_T1_43	W_India_T1_44
Acinetobacter	0.00219	0.00380	0.00000
Actinomyces	0.00000	0.00127	0.00111
Akkermansia	0.00000	0.00000	0.00000
Alistipes	0.01021	0.21495	3.46668
Allisonella	0.00073	0.00063	0.00000
Alloprevotella	6.02305	3.88993	0.00666
Anaerostipes	0.00802	0.01078	0.00666
Bacteroides	0.11081	1.90343	61.24840
Barnesiella	0.00146	0.00063	0.00000
Bifidobacterium	0.19537	0.30942	1.00649
Bilophila	0.00000	0.00254	0.00111
Blautia	0.20704	0.22129	0.51157
Butyricicoccus	0.02479	0.03170	0.05105
Butyricimonas	0.00146	0.00063	0.00000
Campylobacter	0.00146	0.00444	0.00222
Catenibacterium	0.01677	0.22065	0.00000
Clostridium_IV	0.05686	0.49647	0.00111
Clostridium_sensu_stricto	0.33461	0.26947	0.03329
Clostridium_XI	0.17861	0.04312	0.09210
Clostridium_XlVa	0.06634	0.10842	0.24746
Clostridium_XlVb	0.01166	0.02917	0.01665
Collinsella	0.02114	0.03297	0.00000
Comamonas	0.00000	0.00000	0.00000
Coprococcus	0.18954	0.16105	0.09987
Dialister	0.12466	0.32273	0.28630
Dorea	0.16476	0.13569	0.40948
Elusimicrobium	0.00000	0.00000	0.00000
Enterobacter	0.02697	0.00190	0.00111
Erysipelotrichaceae_incertae_sedis	0.04228	0.25806	0.00000
Escherichia_Shigella	0.55623	3.06185	0.21417
Faecalibacterium	9.65059	5.89925	18.85260
Flavonifractor	0.00510	0.00317	0.03218
Fusicatenibacter	0.01968	0.04185	0.37508
Fusobacterium	0.00365	0.01395	0.00000
Gemella	0.00219	0.00190	0.00000
Gemmiger	0.06634	0.27011	0.35732
Granulicatella	0.00073	0.00380	0.00111
Haemophilus	0.16330	0.03234	0.06436
Hallella	0.01677	0.07355	0.00000
Hespellia	0.00146	0.00063	0.00000
Howardella	0.00729	0.00444	0.00000
Intestinimonas	0.02114	0.03107	0.03551
Klebsiella	1.07456	0.84139	0.00777
Lachnospiracea_incertae_sedis	0.17059	0.37536	0.94213
Lactobacillus	0.22745	0.07355	0.02996
Leptotrichia	0.00365	0.00000	0.00111
Megamonas	0.07509	0.00761	0.37064
Megasphaera	0.00292	0.19402	0.13094
Methylobacterium	0.00000	0.00000	0.00000
Mitsuokella	0.06269	0.46730	0.04550
Neisseria	0.00292	0.00444	0.00000
Odoribacter	0.00219	0.03487	0.94102
Olsenella	0.00219	0.00634	0.00555
Oribacterium	0.00146	0.00761	0.00555

Genus	W_India_T1_42	W_India_T1_43	W_India_T1_44
**Oscillibacter**	0.02114	0.03678	0.11763
**Parabacteroides**	0.11008	0.23523	0.72019
**Paraprevotella**	0.00729	0.00697	0.66582
**Parasutterella**	0.03062	0.01902	0.78011
**Peptostreptococcus**	0.00073	0.00254	0.00222
**Prevotella**	77.88780	65.73000	0.33291
**Pseudoflavonifractor**	0.00073	0.00127	0.00000
**Pseudomonas**	0.00000	0.00254	0.00000
**Ralstonia**	0.01604	0.00063	0.00000
**Rhizobium**	0.00000	0.00000	0.00000
**Roseburia**	1.28159	2.39863	4.55529
**Rothia**	0.00073	0.00317	0.00111
**Ruminococcus**	0.02552	0.25489	1.58464
**Ruminococcus2**	0.00802	0.03678	0.23747
**Saccharibacteria_genera_incertae_sedis**	0.00219	0.00761	0.01221
**Slackia**	0.00000	0.00000	0.00000
**Solobacterium**	0.00073	0.00000	0.00111
**Streptococcus**	0.08019	0.08370	0.24746
**Streptophyta**	0.01895	0.11920	0.00111
**Succinivibrio**	0.02624	9.50385	0.01554
**Sutterella**	0.02114	0.64674	0.00333
**Turicibacter**	0.36305	0.10716	0.03107
**Veillonella**	0.01531	0.00571	0.05881
**Weissella**	0.00219	0.00190	0.00666

Genus	W_India_T1_45	W_India_T1_46	W_India_T1_47
**Acinetobacter**	0.00475	0.00000	0.00108
**Actinomyces**	0.01045	0.00111	0.00000
**Akkermansia**	0.00000	0.00000	0.00000
**Alistipes**	0.00380	0.05124	0.21472
**Allisonella**	0.00048	0.00668	0.00000
**Alloprevotella**	0.01140	0.01225	3.09671
**Anaerostipes**	0.00285	0.00111	0.00539
**Bacteroides**	3.17217	0.85771	0.54273
**Barnesiella**	0.00095	0.00000	0.05071
**Bifidobacterium**	2.94457	0.32860	0.08956
**Bilophila**	0.00048	0.01894	0.00000
**Blautia**	0.12117	0.17934	0.80493
**Butyricicoccus**	0.19672	0.05235	0.16724
**Butyricimonas**	0.00000	0.00000	0.04640
**Campylobacter**	0.00285	0.00000	0.00324
**Catenibacterium**	0.00000	0.43888	0.30643
**Clostridium_IV**	0.00048	0.00000	0.35931
**Clostridium_sensu_stricto**	0.43287	0.01225	0.64848
**Clostridium_XI**	0.96315	0.00223	0.24925
**Clostridium_XlVa**	0.04752	0.12921	0.23198
**Clostridium_XlVb**	0.00000	0.01894	0.04963
**Collinsella**	0.01853	0.05681	0.00000
**Comamonas**	0.00000	0.00000	0.00000
**Coprococcus**	0.11404	0.04121	0.85456
**Dialister**	0.47184	0.08020	0.32694
**Dorea**	0.23806	0.14592	0.43591
**Elusimicrobium**	0.00000	0.00000	0.00000
**Enterobacter**	0.00048	0.00000	0.00108
**Erysipelotrichaceae_incertae_sedis**	0.01283	0.00111	0.83514
**Escherichia_Shigella**	22.62240	0.25731	0.03129
**Faecalibacterium**	2.05222	6.63221	14.56320
**Flavonifractor**	0.00713	0.00223	0.00108
**Fusicatenibacter**	0.09123	0.00000	0.04208
**Fusobacterium**	0.00238	0.00111	0.00324
**Gemella**	0.01616	0.00000	0.00108
**Gemmiger**	0.03421	0.04456	0.09171
**Granulicatella**	0.00618	0.00000	0.00324
**Haemophilus**	0.82108	0.01448	0.39275
**Hallella**	0.00000	0.00000	0.00324
**Hespellia**	0.00095	0.00334	0.00432
**Howardella**	0.00000	0.00000	0.00000
**Intestinimonas**	0.00000	0.00000	0.08524
**Klebsiella**	0.15918	0.00000	0.00324
**Lachnospiracea_incertae_sedis**	0.87952	0.94125	0.43591
**Lactobacillus**	4.82811	0.08911	0.00647
**Leptotrichia**	0.00665	0.00223	0.00000
**Megamonas**	0.00285	0.50126	0.00000
**Megasphaera**	0.00095	0.08466	0.00108
**Methylobacterium**	0.00000	0.00000	0.00000
**Mitsuokella**	0.00000	0.00111	0.00000
**Neisseria**	0.03184	0.00000	0.02266
**Odoribacter**	0.00000	0.00000	0.04316
**Olsenella**	0.00000	0.00000	0.00000
**Oribacterium**	0.00475	0.00000	0.00432

Genus	W_India_T1_45	W_India_T1_46	W_India_T1_47
**Oscillibacter**	0.00095	0.00780	0.30428
**Parabacteroides**	0.05749	0.28962	1.38759
**Paraprevotella**	0.00000	0.01782	0.11761
**Parasutterella**	0.00048	0.01559	0.00863
**Peptostreptococcus**	0.00285	0.00000	0.00108
**Prevotella**	53.22090	84.13350	64.40730
**Pseudoflavonifractor**	0.00000	0.00780	0.01618
**Pseudomonas**	0.00285	0.00223	0.00216
**Ralstonia**	0.00000	0.00000	0.00108
**Rhizobium**	0.00000	0.00000	0.00000
**Roseburia**	2.42380	2.00503	5.53847
**Rothia**	0.01996	0.00000	0.00216
**Ruminococcus**	0.00523	0.00557	1.86882
**Ruminococcus2**	0.00000	0.01894	0.07013
**Saccharibacteria_genera_incertae_sedis**	0.00950	0.00000	0.01726
**Slackia**	0.00000	0.00000	0.00000
**Solobacterium**	0.00618	0.00000	0.00432
**Streptococcus**	0.40389	0.03008	0.20177
**Streptophyta**	0.40199	0.00111	0.00432
**Succinivibrio**	0.01045	0.00446	0.01403
**Sutterella**	2.07313	2.31025	0.41002
**Turicibacter**	0.80492	0.00000	0.11653
**Veillonella**	0.13067	0.00334	0.02697
**Weissella**	0.00143	0.00000	0.00000

Genus	W_India_T1_48	W_India_T1_49	W_India_T1_5
**Acinetobacter**	0.00000	0.00104	0.01611
**Actinomyces**	0.00083	0.00209	0.00146
**Akkermansia**	0.00000	0.00000	0.00000
**Alistipes**	0.00000	0.00627	0.02344
**Allisonella**	0.00166	0.01202	0.00732
**Alloprevotella**	5.15178	3.25943	6.13181
**Anaerostipes**	0.00083	0.00470	0.00293
**Bacteroides**	0.14523	0.20218	0.17725
**Barnesiella**	0.00000	0.00575	0.00000
**Bifidobacterium**	0.03485	0.14419	0.08350
**Bilophila**	0.00083	0.00470	0.00000
**Blautia**	0.06390	0.24815	0.22119
**Butyricicoccus**	0.01079	0.06165	0.03223
**Butyricimonas**	0.00000	0.00104	0.00000
**Campylobacter**	0.00166	0.00157	0.00000
**Catenibacterium**	0.14108	0.34585	0.32080
**Clostridium_IV**	0.01660	0.01358	0.00732
**Clostridium_sensu_stricto**	0.05892	0.19434	0.02490
**Clostridium_XI**	0.38589	0.12800	0.17139
**Clostridium_XlVa**	0.04647	0.10710	0.19629
**Clostridium_XlVb**	0.00581	0.02508	0.01025
**Collinsella**	0.00000	0.03657	0.03809
**Comamonas**	0.00000	0.00000	0.00000
**Coprococcus**	0.03900	0.18128	0.17725
**Dialister**	0.25311	0.60967	0.55810
**Dorea**	0.04647	0.21315	0.16113
**Elusimicrobium**	0.00000	0.00000	0.00000
**Enterobacter**	0.00000	0.00522	0.00293
**Erysipelotrichaceae_incertae_sedis**	0.02905	0.09560	0.16553
**Escherichia_Shigella**	0.08299	0.07105	0.05566
**Faecalibacterium**	8.62724	8.46647	3.91551
**Flavonifractor**	0.00000	0.00157	0.00146
**Fusicatenibacter**	0.01162	0.02508	0.01611
**Fusobacterium**	0.00415	0.00104	0.00732
**Gemella**	0.00000	0.00209	0.00000
**Gemmiger**	0.03651	0.15830	0.03809
**Granulicatella**	0.00000	0.00157	0.00293
**Haemophilus**	0.83401	1.11747	1.51611
**Hallella**	0.00664	0.02560	0.00146
**Hespellia**	0.00083	0.00157	0.00146
**Howardella**	0.00000	0.00313	0.00000
**Intestinimonas**	0.00498	0.03553	0.01318
**Klebsiella**	0.02158	0.05851	0.00439
**Lachnospiracea_incertae_sedis**	0.09543	0.52556	0.17139
**Lactobacillus**	0.03402	0.24972	0.03955
**Leptotrichia**	0.00083	0.00157	0.01172
**Megamonas**	0.00083	0.00052	0.20947
**Megasphaera**	0.21162	0.28002	0.11865
**Methylobacterium**	0.00000	0.00000	0.00000
**Mitsuokella**	0.14689	0.38190	0.02930
**Neisseria**	0.00830	0.01724	0.05859
**Odoribacter**	0.00000	0.00000	0.04541
**Olsenella**	0.00000	0.04545	0.00000
**Oribacterium**	0.00996	0.01776	0.00732

Genus	W_India_T1_48	W_India_T1_49	W_India_T1_5
**Oscillibacter**	0.01494	0.05068	0.01318
**Parabacteroides**	0.00249	0.01515	0.14648
**Paraprevotella**	0.00000	0.00418	0.04102
**Parasutterella**	0.00000	0.02037	0.00000
**Peptostreptococcus**	0.00332	0.00000	0.00146
**Prevotella**	59.85790	63.49800	80.40630
**Pseudoflavonifractor**	0.00000	0.00784	0.00439
**Pseudomonas**	0.00166	0.00522	0.00586
**Ralstonia**	0.00000	0.01254	0.00439
**Rhizobium**	0.00000	0.00000	0.00000
**Roseburia**	1.28297	2.95119	3.13036
**Rothia**	0.00083	0.00993	0.00146
**Ruminococcus**	0.02656	0.11807	0.02783
**Ruminococcus2**	0.00664	0.06008	0.04248
**Saccharibacteria_genera_incertae_sedis**	0.00083	0.00731	0.00146
**Slackia**	0.00000	0.00000	0.00000
**Solobacterium**	0.00000	0.00000	0.00000
**Streptococcus**	0.34024	0.19905	0.65625
**Streptophyta**	0.00498	0.01202	0.00000
**Succinivibrio**	21.25440	14.46500	0.00000
**Sutterella**	0.47966	0.68177	0.28125
**Turicibacter**	0.03817	0.10658	0.26514
**Veillonella**	0.02407	0.06217	0.02637
**Weissella**	0.00000	0.00104	0.00586

Genus	W_India_T1_50	W_India_T1_51	W_India_T1_52
**Acinetobacter**	0.00080	0.00482	0.00033
**Actinomyces**	0.00239	0.00193	0.00033
**Akkermansia**	0.00000	0.00000	0.00000
**Alistipes**	0.01116	0.00193	0.89188
**Allisonella**	0.01355	0.01253	0.00428
**Alloprevotella**	3.42295	9.04866	0.00296
**Anaerostipes**	0.00159	0.00000	0.00000
**Bacteroides**	0.52863	0.02121	9.77148
**Barnesiella**	0.00718	0.00096	0.00066
**Bifidobacterium**	0.48159	0.05978	0.06552
**Bilophila**	0.00877	0.00096	0.04379
**Blautia**	0.42338	0.05785	0.31013
**Butyricicoccus**	0.09090	0.00482	0.16033
**Butyricimonas**	0.00877	0.00000	0.14782
**Campylobacter**	0.00319	0.00193	0.00033
**Catenibacterium**	0.65381	0.25936	0.09383
**Clostridium_IV**	0.01196	0.00386	0.06980
**Clostridium_sensu_stricto**	0.21289	0.54186	0.17515
**Clostridium_XI**	0.09488	0.76747	0.22980
**Clostridium_XlVa**	0.14272	0.14462	0.32067
**Clostridium_XlVb**	0.01754	0.00771	0.01910
**Collinsella**	0.11003	0.03085	0.00132
**Comamonas**	0.00000	0.00000	0.00000
**Coprococcus**	0.32133	0.01157	0.28050
**Dialister**	1.00225	0.16198	0.41746
**Dorea**	0.31415	0.09931	0.44380
**Elusimicrobium**	0.00000	0.00000	0.00000
**Enterobacter**	0.01116	0.02025	0.00198
**Erysipelotrichaceae_incertae_sedis**	0.15070	0.07520	0.29203
**Escherichia_Shigella**	0.11721	0.20055	0.03095
**Faecalibacterium**	7.03009	1.55616	13.49010
**Flavonifractor**	0.00319	0.00193	0.00757
**Fusicatenibacter**	0.06060	0.01350	0.03984
**Fusobacterium**	0.00239	0.01157	0.00263
**Gemella**	0.00558	0.00675	0.00165
**Gemmiger**	0.26073	0.03471	0.61994
**Granulicatella**	0.00399	0.00289	0.00263
**Haemophilus**	0.15229	0.16294	0.07309
**Hallella**	0.01595	0.00193	0.00000
**Hespellia**	0.00080	0.00000	0.00560
**Howardella**	0.01276	0.00193	0.00000
**Intestinimonas**	0.04943	0.00386	0.02700
**Klebsiella**	0.07017	0.04532	0.00494
**Lachnospiracea_incertae_sedis**	0.79893	0.56018	0.32758
**Lactobacillus**	0.62830	0.00000	0.00856
**Leptotrichia**	0.00478	0.00578	0.00099
**Megamonas**	0.00638	0.00000	0.00033
**Megasphaera**	0.44013	0.04821	0.01218
**Methylobacterium**	0.00000	0.00000	0.00000
**Mitsuokella**	0.40744	0.11763	0.00395
**Neisseria**	0.01754	0.03375	0.00395
**Odoribacter**	0.00877	0.00096	0.13959
**Olsenella**	0.10126	0.00000	0.00099
**Oribacterium**	0.00159	0.10702	0.00066

Genus	W_India_T1_50	W_India_T1_51	W_India_T1_52
**Oscillibacter**	0.05023	0.00868	0.01975
**Parabacteroides**	0.04066	0.00386	0.75953
**Paraprevotella**	0.00319	0.00000	0.54454
**Parasutterella**	0.01914	0.00000	0.01942
**Peptostreptococcus**	0.00080	0.00096	0.00000
**Prevotella**	55.75600	83.22550	59.20470
**Pseudoflavonifractor**	0.00558	0.00000	0.00033
**Pseudomonas**	0.00080	0.00482	0.00033
**Ralstonia**	0.02153	0.00193	0.02173
**Rhizobium**	0.00000	0.00000	0.00000
**Roseburia**	4.89882	1.90326	8.33407
**Rothia**	0.00478	0.00482	0.00198
**Ruminococcus**	0.05821	0.00193	0.39442
**Ruminococcus2**	0.11641	0.00675	0.12478
**Saccharibacteria_genera_incertae_sedis**	0.00319	0.00289	0.00395
**Slackia**	0.00000	0.00000	0.00000
**Solobacterium**	0.00080	0.00193	0.00000
**Streptococcus**	0.29741	0.03567	0.54356
**Streptophyta**	0.02950	0.00096	0.00263
**Succinivibrio**	18.95100	0.00096	0.00033
**Sutterella**	1.21195	0.32396	1.28399
**Turicibacter**	0.02870	0.05206	0.05860
**Veillonella**	0.07335	0.02314	0.00296
**Weissella**	0.00239	0.00096	0.00000

Genus	W_India_T1_53	W_India_T1_54	W_India_T1_55
**Acinetobacter**	0.00652	0.00000	0.00121
**Actinomyces**	0.00000	0.00852	0.00243
**Akkermansia**	0.00000	0.00000	0.00000
**Alistipes**	0.21197	0.00061	0.00728
**Allisonella**	0.00326	0.02374	0.00000
**Alloprevotella**	2.43278	4.73736	3.75253
**Anaerostipes**	0.06033	0.00000	0.00000
**Bacteroides**	5.11667	0.12355	2.24133
**Barnesiella**	0.13044	0.00061	0.00000
**Bifidobacterium**	1.36966	0.11259	0.09824
**Bilophila**	0.01141	0.00183	0.01334
**Blautia**	0.95224	0.21545	0.42450
**Butyricicoccus**	0.27230	0.04260	0.11522
**Butyricimonas**	0.03261	0.00000	0.00000
**Campylobacter**	0.00652	0.01278	0.00243
**Catenibacterium**	0.56906	2.11976	0.41964
**Clostridium_IV**	0.16306	0.01339	0.00121
**Clostridium_sensu_stricto**	0.19404	0.79423	0.26683
**Clostridium_XI**	0.22339	1.58297	0.11158
**Clostridium_XlVa**	0.18588	0.18867	0.26683
**Clostridium_XlVb**	0.06848	0.05051	0.21952
**Collinsella**	0.02283	0.23370	0.00000
**Comamonas**	0.00000	0.00000	0.00000
**Coprococcus**	0.55602	0.09920	0.44269
**Dialister**	1.00931	1.51116	4.00723
**Dorea**	0.26252	0.26657	0.36870
**Elusimicrobium**	0.00000	0.00000	0.00000
**Enterobacter**	0.01141	0.10103	0.00485
**Erysipelotrichaceae_incertae_sedis**	0.09783	0.21119	0.68768
**Escherichia_Shigella**	0.94083	0.27570	0.13099
**Faecalibacterium**	23.31520	16.40610	11.66630
**Flavonifractor**	0.00978	0.00061	0.02911
**Fusicatenibacter**	0.15001	0.02191	0.02668
**Fusobacterium**	0.00489	0.00061	0.56155
**Gemella**	0.00489	0.00304	0.00364
**Gemmiger**	0.49569	0.09738	0.22559
**Granulicatella**	0.00163	0.01156	0.00364
**Haemophilus**	0.60657	1.55011	0.09096
**Hallella**	0.16632	0.00304	0.00485
**Hespellia**	0.00163	0.00304	0.00364
**Howardella**	0.02283	0.00061	0.00728
**Intestinimonas**	0.05055	0.02374	0.09824
**Klebsiella**	0.42884	0.51731	0.00000
**Lachnospiracea_incertae_sedis**	0.19893	0.35177	1.13522
**Lactobacillus**	0.81038	0.14850	0.00243
**Leptotrichia**	0.00000	0.00365	0.00000
**Megamonas**	0.00000	0.27083	1.05760
**Megasphaera**	0.86582	0.77475	0.00000
**Methylobacterium**	0.00000	0.00000	0.00000
**Mitsuokella**	0.82343	0.19475	0.00000
**Neisseria**	0.00489	0.01522	0.00364
**Odoribacter**	0.09783	0.00000	0.05215
**Olsenella**	0.04402	0.00000	0.00000
**Oribacterium**	0.00163	0.00365	0.01092

Genus	W_India_T1_53	W_India_T1_54	W_India_T1_55
**Oscillibacter**	0.22991	0.02252	0.06549
**Parabacteroides**	0.47286	0.08399	0.22801
**Paraprevotella**	0.01141	0.00000	0.00000
**Parasutterella**	0.00000	0.01035	0.00000
**Peptostreptococcus**	0.00326	0.00183	0.02304
**Prevotella**	45.23800	60.57900	65.28000
**Pseudoflavonifractor**	0.00326	0.00061	0.00485
**Pseudomonas**	0.00000	0.00000	0.00000
**Ralstonia**	0.02609	0.00000	0.00000
**Rhizobium**	0.00000	0.00000	0.00000
**Roseburia**	5.01068	4.09650	1.92478
**Rothia**	0.00326	0.02617	0.00243
**Ruminococcus**	0.65548	0.00061	0.03760
**Ruminococcus2**	0.22991	0.04504	0.59308
**Saccharibacteria_genera_incertae_sedis**	0.01467	0.01400	0.00728
**Slackia**	0.00000	0.00000	0.00000
**Solobacterium**	0.00000	0.00061	0.00243
**Streptococcus**	0.97507	0.41263	1.88597
**Streptophyta**	0.04402	0.00791	0.00243
**Succinivibrio**	1.24737	0.00122	0.00243
**Sutterella**	2.02025	1.15817	1.14492
**Turicibacter**	0.25274	0.15459	0.00970
**Veillonella**	0.11088	0.17650	0.02790
**Weissella**	0.05707	0.03895	0.00000

Genus	W_India_T1_56	W_India_T1_57	W_India_T1_58
**Acinetobacter**	0.00000	0.00000	0.00097
**Actinomyces**	0.00000	0.00114	0.00146
**Akkermansia**	0.00000	0.00000	0.00000
**Alistipes**	0.14642	0.00000	0.00000
**Allisonella**	0.00339	0.00114	0.03992
**Alloprevotella**	2.39605	4.00169	5.25004
**Anaerostipes**	0.00085	0.06835	0.00000
**Bacteroides**	2.57590	0.76548	0.00390
**Barnesiella**	0.29496	0.00911	0.00049
**Bifidobacterium**	0.13753	0.05354	0.08131
**Bilophila**	0.00465	0.01253	0.00000
**Blautia**	0.31188	1.06848	0.05307
**Butyricicoccus**	0.02370	0.06721	0.02921
**Butyricimonas**	0.05967	0.00000	0.00146
**Campylobacter**	0.00085	0.01709	0.01315
**Catenibacterium**	0.62165	0.17314	0.55651
**Clostridium_IV**	0.00042	0.54791	0.00049
**Clostridium_sensu_stricto**	0.02793	0.27566	0.52632
**Clostridium_XI**	0.00085	0.12530	0.65875
**Clostridium_XlVa**	0.07871	0.18454	0.05210
**Clostridium_XlVb**	0.02412	0.28364	0.02970
**Collinsella**	0.00000	0.10708	0.00000
**Comamonas**	0.00000	0.00000	0.00000
**Coprococcus**	0.10410	0.31439	0.10760
**Dialister**	0.36436	0.78257	1.64858
**Dorea**	0.14388	0.35198	0.08423
**Elusimicrobium**	0.00000	0.00000	0.00000
**Enterobacter**	0.00042	0.03076	0.00536
**Erysipelotrichaceae_incertae_sedis**	0.02454	0.10594	0.08423
**Escherichia_Shigella**	0.08760	0.20846	0.01412
**Faecalibacterium**	3.44976	13.80260	6.62353
**Flavonifractor**	0.00085	0.32465	0.00000
**Fusicatenibacter**	0.02666	0.08088	0.01217
**Fusobacterium**	0.00000	0.00228	0.00292
**Gemella**	0.00042	0.00000	0.00292
**Gemmiger**	0.02370	0.12189	0.09397
**Granulicatella**	0.00000	0.00114	0.00097
**Haemophilus**	0.00296	0.50235	1.75131
**Hallella**	0.00000	0.00228	0.01169
**Hespellia**	0.00000	0.00683	0.00243
**Howardella**	0.00000	0.00797	0.00049
**Intestinimonas**	0.01608	0.04101	0.01169
**Klebsiella**	0.00635	0.19707	0.00876
**Lachnospiracea_incertae_sedis**	0.15319	0.85319	0.75223
**Lactobacillus**	0.15404	0.05468	0.10419
**Leptotrichia**	0.00000	0.00000	0.00000
**Megamonas**	0.00254	0.00000	0.00000
**Megasphaera**	0.16039	0.00000	0.22348
**Methylobacterium**	0.00000	0.00000	0.00000
**Mitsuokella**	0.09691	0.20390	0.19573
**Neisseria**	0.00000	0.01139	0.01509
**Odoribacter**	0.01820	0.07404	0.00049
**Olsenella**	0.00085	0.00683	0.00633
**Oribacterium**	0.00423	0.00342	0.24782

Genus	W_India_T1_56	W_India_T1_57	W_India_T1_58
**Oscillibacter**	0.01185	0.11277	0.00438
**Parabacteroides**	0.59161	0.70739	0.00000
**Paraprevotella**	0.22005	0.02392	0.00097
**Parasutterella**	0.00042	0.01367	0.00000
**Peptostreptococcus**	0.00042	0.00000	0.00487
**Prevotella**	47.35680	69.13130	73.39730
**Pseudoflavonifractor**	0.00042	0.00570	0.00146
**Pseudomonas**	0.00042	0.00000	0.00146
**Ralstonia**	0.00000	0.00000	0.00000
**Rhizobium**	0.00000	0.00000	0.00000
**Roseburia**	0.87895	1.70069	2.53422
**Rothia**	0.00000	0.00228	0.01363
**Ruminococcus**	0.15108	1.58906	0.05989
**Ruminococcus2**	0.02243	0.17770	0.04918
**Saccharibacteria_genera_incertae_sedis**	0.00169	0.00456	0.00682
**Slackia**	0.00000	0.00000	0.00000
**Solobacterium**	0.00000	0.00000	0.00000
**Streptococcus**	0.01016	0.16745	0.17138
**Streptophyta**	0.00169	0.02278	0.15824
**Succinivibrio**	38.77640	0.00114	3.55228
**Sutterella**	0.36351	0.20276	0.14120
**Turicibacter**	0.00042	0.12758	0.09543
**Veillonella**	0.00042	0.02506	0.06329
**Weissella**	0.00254	0.04443	0.00000

Genus	W_India_T1_59	W_India_T1_6	W_India_T1_60
Acinetobacter	0.00257	0.00042	0.08299
Actinomyces	0.02145	0.00000	0.04453
Akkermansia	0.00000	0.00000	0.00000
Alistipes	0.06777	0.00000	0.03239
Allisonella	0.01201	0.00000	0.00607
Alloprevotella	4.16145	0.00296	6.23419
Anaerostipes	0.04461	0.00042	0.00405
Bacteroides	1.75860	6.76213	2.74871
Barnesiella	0.00000	0.00000	0.03846
Bifidobacterium	0.93249	0.05868	0.13157
Bilophila	0.01287	0.00000	0.03643
Blautia	0.38432	0.44200	0.66795
Butyricicoccus	0.05233	0.06248	0.11740
Butyricimonas	0.00172	0.00000	0.02227
Campylobacter	0.00343	0.00042	0.01417
Catenibacterium	1.18641	0.10934	0.15383
Clostridium_IV	0.02402	0.00000	0.06477
Clostridium_sensu_stricto	0.07978	0.01604	0.03036
Clostridium_XI	0.01716	0.04011	0.11740
Clostridium_XlVa	0.13039	0.51208	0.04251
Clostridium_XlVb	0.10380	0.00675	0.03239
Collinsella	0.17586	0.00084	0.03239
Comamonas	0.00000	0.00000	0.00000
Coprococcus	0.35515	0.02449	1.24077
Dialister	0.56790	0.47577	0.13764
Dorea	0.49927	0.04517	0.25301
Elusimicrobium	0.00000	0.00000	0.00000
Enterobacter	0.00086	0.00000	0.02429
Erysipelotrichaceae_incertae_sedis	0.18787	0.01435	0.07084
Escherichia_Shigella	0.02145	3.51616	0.02631
Faecalibacterium	17.42730	3.07964	14.61190
Flavonifractor	0.00257	0.00591	0.00202
Fusicatenibacter	0.04375	0.03293	0.05870
Fusobacterium	0.01373	0.00042	0.08501
Gemella	0.00429	0.00000	0.03441
Gemmiger	0.25993	0.00464	0.26516
Granulicatella	0.01115	0.00042	0.03036
Haemophilus	0.10037	0.01984	0.17002
Hallella	0.11838	0.00000	0.08299
Hespellia	0.00086	0.00000	0.00607
Howardella	0.00000	0.00000	0.00202
Intestinimonas	0.06691	0.00000	0.05263
Klebsiella	0.02659	0.00000	0.15383
Lachnospiracea_incertae_sedis	0.33714	0.12580	0.14573
Lactobacillus	0.13640	0.00802	0.06679
Leptotrichia	0.01458	0.00000	0.06882
Megamonas	0.20417	0.00000	0.03441
Megasphaera	0.17758	0.01309	0.21455
Methylobacterium	0.00000	0.00000	0.00000
Mitsuokella	0.10123	0.00000	0.35017
Neisseria	0.01716	0.00000	0.33802
Odoribacter	0.01973	0.09372	0.02227
Olsenella	0.01544	0.00000	0.06072
Oribacterium	0.00858	0.00338	0.00810

Genus	W_India_T1_59	W_India_T1_6	W_India_T1_60
**Oscillibacter**	0.37059	0.00422	0.06477
**Parabacteroides**	0.31826	0.00464	0.74082
**Paraprevotella**	0.06348	0.00000	0.47566
**Parasutterella**	0.00600	0.00000	0.00202
**Peptostreptococcus**	0.00257	0.00042	0.01214
**Prevotella**	46.08560	82.96210	59.54460
**Pseudoflavonifractor**	0.02745	0.00000	0.00202
**Pseudomonas**	0.00086	0.00042	0.02631
**Ralstonia**	0.01115	0.00042	0.07084
**Rhizobium**	0.00000	0.00000	0.00000
**Roseburia**	2.45861	1.23819	0.91894
**Rothia**	0.01458	0.00000	0.10728
**Ruminococcus**	0.18272	0.03166	1.41484
**Ruminococcus2**	0.07120	0.03208	0.02024
**Saccharibacteria_genera_incertae_sedis**	0.02145	0.00549	0.04453
**Slackia**	0.00000	0.00000	0.00000
**Solobacterium**	0.00515	0.00084	0.01417
**Streptococcus**	0.25135	0.00253	1.16183
**Streptophyta**	0.01287	0.00042	0.02834
**Succinivibrio**	18.25170	0.00042	1.61320
**Sutterella**	1.54757	0.00127	0.85214
**Turicibacter**	0.02831	0.00000	0.05060
**Veillonella**	0.08493	0.00338	0.17205
**Weissella**	0.00000	0.00000	0.00000

Genus	W_India_T1_61	W_India_T1_62	W_India_T1_63
Acinetobacter	0.00000	0.00570	0.00382
Actinomyces	0.00049	0.00228	0.00000
Akkermansia	0.00000	0.00000	0.00000
Alistipes	0.09139	0.01825	3.25694
Allisonella	0.02015	0.00570	0.00000
Alloprevotella	2.62379	6.53567	0.01146
Anaerostipes	0.00000	0.00228	0.00000
Bacteroides	0.23093	2.46100	66.70100
Barnesiella	0.00000	0.00000	0.00000
Bifidobacterium	0.42796	0.44134	0.51311
Bilophila	0.00000	0.00000	0.00000
Blautia	0.07223	0.24405	0.34632
Butyricicoccus	0.05159	0.07527	0.03565
Butyricimonas	0.00098	0.00000	0.00000
Campylobacter	0.00000	0.00114	0.00255
Catenibacterium	0.02702	0.05588	0.00000
Clostridium_IV	0.10613	0.00114	0.00000
Clostridium_sensu_stricto	0.00049	1.77105	0.02165
Clostridium_XI	0.00000	0.20185	0.07257
Clostridium_XlVa	0.02604	0.12887	0.25974
Clostridium_XlVb	0.05208	0.04448	0.00891
Collinsella	0.00147	0.12430	0.07003
Comamonas	0.00000	0.00000	0.00000
Coprococcus	1.90102	0.15396	0.07512
Dialister	0.37244	0.29423	0.14260
Dorea	0.21374	0.14027	0.36542
Elusimicrobium	0.00000	0.00000	0.00000
Enterobacter	0.00000	0.00114	0.00000
Erysipelotrichaceae_incertae_sedis	0.00049	0.01254	0.00000
Escherichia_Shigella	0.54785	0.47897	0.18335
Faecalibacterium	8.91255	10.04470	14.28060
Flavonifractor	0.00295	0.00000	0.01910
Fusicatenibacter	0.03734	0.01825	0.30048
Fusobacterium	0.00098	0.00114	0.00255
Gemella	0.00000	0.00456	0.00509
Gemmiger	0.08943	0.20071	0.26611
Granulicatella	0.00000	0.00114	0.00382
Haemophilus	0.00934	0.14597	0.08021
Hallella	0.00197	0.00114	0.00000
Hespellia	0.00098	0.00456	0.00000
Howardella	0.00000	0.01711	0.00000
Intestinimonas	0.03783	0.01026	0.00891
Klebsiella	0.00344	0.00000	0.00127
Lachnospiracea_incertae_sedis	0.49037	0.88952	1.26305
Lactobacillus	0.16067	0.15282	0.01401
Leptotrichia	0.00098	0.00228	0.00637
Megamonas	0.02408	0.08781	0.26865
Megasphaera	0.10810	0.00000	0.10568
Methylobacterium	0.00000	0.00000	0.00000
Mitsuokella	0.17787	0.45274	0.02928
Neisseria	0.00098	0.02167	0.02165
Odoribacter	0.12382	0.02281	1.02878
Olsenella	0.00098	0.00114	0.00255
Oribacterium	0.00147	0.00456	0.00509

Genus	W_India_T1_61	W_India_T1_62	W_India_T1_63
**Oscillibacter**	0.01867	0.00342	0.08149
**Parabacteroides**	0.29088	0.30791	0.90400
**Paraprevotella**	0.00000	0.09237	0.57932
**Parasutterella**	0.03439	0.00684	0.66972
**Peptostreptococcus**	0.00000	0.00114	0.00000
**Prevotella**	80.48610	68.57380	0.66208
**Pseudoflavonifractor**	0.00393	0.00000	0.00000
**Pseudomonas**	0.00049	0.02167	0.00637
**Ralstonia**	0.00147	0.00000	0.00382
**Rhizobium**	0.00000	0.00000	0.00000
**Roseburia**	1.79194	1.64105	4.69697
**Rothia**	0.00049	0.00456	0.01401
**Ruminococcus**	0.16558	0.83478	1.40438
**Ruminococcus2**	0.06191	0.00114	0.21263
**Saccharibacteria_genera_incertae_sedis**	0.00049	0.00114	0.01146
**Slackia**	0.00000	0.00000	0.00000
**Solobacterium**	0.00000	0.00000	0.00382
**Streptococcus**	0.01278	0.58389	0.19353
**Streptophyta**	0.00786	0.01254	0.00255
**Succinivibrio**	0.00295	0.02281	0.01019
**Sutterella**	0.15723	2.30704	0.01019
**Turicibacter**	0.00000	0.01254	0.02165
**Veillonella**	0.00049	0.01711	0.06239
**Weissella**	0.00049	0.00684	0.00764

Genus	W_India_T1_64	W_India_T1_65	W_India_T1_66
Acinetobacter	0.00520	0.01637	0.00077
Actinomyces	0.00624	0.00546	0.00386
Akkermansia	0.00000	0.00000	0.00000
Alistipes	2.69982	6.43665	0.16686
Allisonella	0.00000	0.00327	0.00000
Alloprevotella	0.01351	0.05892	6.33893
Anaerostipes	0.00658	0.02728	0.00077
Bacteroides	54.26490	15.26400	0.70759
Barnesiella	0.00069	0.00436	0.00463
Bifidobacterium	1.29360	0.52375	0.71145
Bilophila	0.00000	0.05456	0.03862
Blautia	1.80681	0.22150	0.56391
Butyricicoccus	0.15733	1.24499	0.18848
Butyricimonas	0.00000	0.39390	0.01777
Campylobacter	0.00139	0.00546	0.00309
Catenibacterium	0.00069	0.00436	0.02240
Clostridium_IV	0.00035	3.40328	0.73076
Clostridium_sensu_stricto	0.02079	0.06874	0.35070
Clostridium_XI	0.15317	0.43100	0.39628
Clostridium_XlVa	0.36316	0.14294	0.14368
Clostridium_XlVb	0.02668	0.25205	0.03244
Collinsella	0.19960	0.01637	0.09115
Comamonas	0.00000	0.00000	0.00000
Coprococcus	0.20030	0.73761	1.62374
Dialister	0.60539	0.14403	3.67157
Dorea	1.25583	1.05841	0.37002
Elusimicrobium	0.00000	0.00000	0.00000
Enterobacter	0.00139	0.00327	0.00463
Erysipelotrichaceae_incertae_sedis	0.00000	0.00109	0.00077
Escherichia_Shigella	0.15109	0.17567	0.03553
Faecalibacterium	17.01190	8.74769	3.48077
Flavonifractor	0.07208	0.00436	0.00154
Fusicatenibacter	1.23919	0.03601	0.09424
Fusobacterium	0.00347	0.00982	0.00077
Gemella	0.00139	0.00327	0.01468
Gemmiger	0.90722	0.49101	0.17690
Granulicatella	0.00416	0.00546	0.00386
Haemophilus	0.06653	0.03164	1.46848
Hallella	0.00035	0.01528	0.00077
Hespellia	0.00000	0.00327	0.00309
Howardella	0.00000	0.00327	0.00000
Intestinimonas	0.01213	0.41900	0.03167
Klebsiella	0.00208	0.03601	0.02626
Lachnospiracea_incertae_sedis	1.23711	0.23241	0.72535
Lactobacillus	0.02911	0.03928	0.21398
Leptotrichia	0.00693	0.00327	0.00000
Megamonas	0.80950	0.01528	0.00541
Megasphaera	0.59915	0.00655	1.74811
Methylobacterium	0.00000	0.00000	0.00000
Mitsuokella	0.24223	0.01637	0.00077
Neisseria	0.01040	0.02182	0.00463
Odoribacter	0.63380	0.36662	0.02704
Olsenella	0.01663	0.00109	0.02935
Oribacterium	0.00243	0.00655	0.00309

Genus	W_India_T1_64	W_India_T1_65	W_India_T1_66
Oscillibacter	0.07520	0.22587	0.06721
Parabacteroides	0.80430	10.44220	1.44067
Paraprevotella	0.71870	0.61541	0.03785
Parasutterella	0.44044	0.02728	0.00541
Peptostreptococcus	0.00069	0.00000	0.00386
Prevotella	0.40336	35.65420	55.42280
Pseudoflavonifractor	0.00000	0.00218	0.00077
Pseudomonas	0.00347	0.00873	0.00077
Ralstonia	0.00797	0.02837	0.00386
Rhizobium	0.00000	0.00000	0.00000
Roseburia	6.72927	0.29679	2.39390
Rothia	0.00693	0.00655	0.01390
Ruminococcus	3.31422	3.30726	0.22556
Ruminococcus2	0.44841	0.23132	0.11973
Saccharibacteria_genera_incertae_sedis	0.01005	0.00436	0.00463
Slackia	0.00000	0.00000	0.00000
Solobacterium	0.00069	0.00327	0.00154
Streptococcus	0.23356	0.05019	3.14166
Streptophyta	0.00728	0.03819	0.08420
Succinivibrio	0.00243	0.02837	0.03785
Sutterella	0.00450	0.16367	1.03512
Turicibacter	0.02287	0.16149	0.00850
Veillonella	0.05856	0.03492	0.03862
Weissella	0.01074	0.00000	0.00077

Genus	W_India_T1_67	W_India_T1_68	W_India_T1_69
Acinetobacter	0.00061	0.00537	0.00000
Actinomyces	0.00000	0.00376	0.00069
Akkermansia	0.00000	0.00000	0.00000
Alistipes	0.00368	0.11060	0.01306
Allisonella	0.00430	0.00322	0.00179
Alloprevotella	5.05350	10.21370	2.82710
Anaerostipes	0.00000	0.00161	0.00014
Bacteroides	0.02026	6.11672	0.08898
Barnesiella	0.00000	0.34253	0.01939
Bifidobacterium	0.06691	2.96897	0.17232
Bilophila	0.00552	0.00000	0.00701
Blautia	0.06876	0.65661	0.09847
Butyricicoccus	0.01044	0.20455	0.02599
Butyricimonas	0.00123	0.00000	0.01582
Campylobacter	0.00000	0.01664	0.00028
Catenibacterium	0.33702	0.78922	0.32868
Clostridium_IV	0.00307	0.01557	0.03672
Clostridium_sensu_stricto	0.02087	1.65253	0.03576
Clostridium_XI	0.02271	0.45689	0.02379
Clostridium_XlVa	0.17312	0.15033	0.11016
Clostridium_XlVb	0.03438	0.06496	0.00784
Collinsella	0.00000	0.84183	0.00000
Comamonas	0.00000	0.00000	0.00000
Coprococcus	0.01289	0.90089	0.09379
Dialister	2.16333	1.50650	0.67841
Dorea	0.06630	0.37045	0.10796
Elusimicrobium	0.00000	0.00000	0.00000
Enterobacter	0.02517	0.00161	0.00000
Erysipelotrichaceae_incertae_sedis	0.04972	0.29153	0.06849
Escherichia_Shigella	0.89136	0.15301	1.05055
Faecalibacterium	8.16897	25.69420	12.51560
Flavonifractor	0.00000	0.00000	0.00000
Fusicatenibacter	0.00061	0.32159	0.01375
Fusobacterium	0.00246	0.09181	0.00041
Gemella	0.00123	0.01772	0.00083
Gemmiger	0.03622	1.28423	0.06161
Granulicatella	0.00061	0.01825	0.00014
Haemophilus	0.25353	4.00032	0.00729
Hallella	0.00675	0.00376	0.00014
Hespellia	0.00246	0.00107	0.00316
Howardella	0.00982	0.00000	0.00880
Intestinimonas	0.01842	0.04402	0.02145
Klebsiella	0.35544	1.67132	0.00000
Lachnospiracea_incertae_sedis	0.01351	0.52293	0.09503
Lactobacillus	0.12523	1.60958	0.11085
Leptotrichia	0.00000	0.00322	0.00028
Megamonas	0.01780	0.00000	0.00000
Megasphaera	0.37079	0.16160	0.08980
Methylobacterium	0.00000	0.00000	0.00000
Mitsuokella	1.92269	0.16858	0.88071
Neisseria	0.00000	0.01127	0.00096
Odoribacter	0.00000	0.02684	0.00358
Olsenella	0.00061	0.23623	0.00371
Oribacterium	0.00552	0.00913	0.00426

Genus	W_India_T1_67	W_India_T1_68	W_India_T1_69
**Oscillibacter**	0.00798	0.03651	0.00605
**Parabacteroides**	0.01289	0.85257	0.02407
**Paraprevotella**	0.00000	0.06443	0.03617
**Parasutterella**	0.00000	0.37904	0.00468
**Peptostreptococcus**	0.00184	0.05154	0.00165
**Prevotella**	77.80810	19.76590	77.47850
**Pseudoflavonifractor**	0.00000	0.00107	0.00110
**Pseudomonas**	0.00000	0.00215	0.00000
**Ralstonia**	0.00061	0.00591	0.00014
**Rhizobium**	0.00000	0.00000	0.00000
**Roseburia**	1.08351	6.64125	1.11643
**Rothia**	0.00246	0.05852	0.00124
**Ruminococcus**	0.09577	1.25738	0.09297
**Ruminococcus2**	0.00982	0.06174	0.00399
**Saccharibacteria_genera_incertae_sedis**	0.00123	0.02470	0.00220
**Slackia**	0.00000	0.00000	0.00000
**Solobacterium**	0.00000	0.00107	0.00028
**Streptococcus**	0.16022	2.95125	0.91647
**Streptophyta**	0.00491	0.09664	0.00193
**Succinivibrio**	0.00737	0.00483	0.00193
**Sutterella**	0.31492	1.68635	0.64499
**Turicibacter**	0.06937	0.29958	0.00055
**Veillonella**	0.00859	1.66971	0.02173
**Weissella**	0.00184	0.02523	0.00000

Genus	W_India_T1_7	W_India_T1_70	W_India_T1_71
Acinetobacter	0.00000	0.00075	0.00183
Actinomyces	0.00068	0.00075	0.00092
Akkermansia	0.00000	0.00000	0.00000
Alistipes	0.85638	0.36993	0.00000
Allisonella	0.00000	0.00598	0.00275
Alloprevotella	0.00068	3.42805	5.91957
Anaerostipes	0.00068	0.05530	0.00000
Bacteroides	55.87230	1.49617	0.42178
Barnesiella	2.40099	0.01270	0.00092
Bifidobacterium	0.11811	0.55752	0.12287
Bilophila	0.03268	0.02093	0.00092
Blautia	0.66815	0.29669	0.15496
Butyricicoccus	0.11369	0.07698	0.03393
Butyricimonas	0.77367	0.08221	0.00092
Campylobacter	0.00000	0.00000	0.00183
Catenibacterium	0.74644	0.15395	0.47496
Clostridium_IV	0.37271	0.44317	0.00092
Clostridium_sensu_stricto	0.37850	0.02541	0.29066
Clostridium_XI	0.46461	0.03288	0.35851
Clostridium_XlVa	0.44827	0.10762	0.03943
Clostridium_XlVb	0.01668	0.10388	0.02109
Collinsella	0.02212	0.04559	0.02292
Comamonas	0.00000	0.00000	0.00000
Coprococcus	0.01464	0.09566	0.17055
Dialister	0.24575	1.30784	0.49880
Dorea	0.33186	0.29819	0.13112
Elusimicrobium	0.00000	0.00000	0.00000
Enterobacter	0.00000	0.01345	0.04768
Erysipelotrichaceae_incertae_sedis	0.14738	0.19879	0.09628
Escherichia_Shigella	0.24064	0.43944	0.02842
Faecalibacterium	6.44020	25.45660	5.72793
Flavonifractor	0.03097	0.00000	0.00000
Fusicatenibacter	0.08816	0.04932	0.01284
Fusobacterium	0.00068	0.00149	0.00000
Gemella	0.00034	0.00299	0.00000
Gemmiger	0.86931	0.24363	0.09811
Granulicatella	0.00034	0.00075	0.00092
Haemophilus	0.00749	0.04335	0.62350
Hallella	0.00000	0.16740	0.01375
Hespellia	0.00613	0.00075	0.00092
Howardella	0.00000	0.00149	0.00458
Intestinimonas	0.06297	0.06053	0.02201
Klebsiella	0.00034	0.20253	0.04860
Lachnospiracea_incertae_sedis	0.05344	0.10762	0.35576
Lactobacillus	0.05072	0.06950	0.08436
Leptotrichia	0.00000	0.00149	0.00000
Megamonas	0.52179	0.00598	0.00000
Megasphaera	0.01770	0.25484	0.29891
Methylobacterium	0.00000	0.00000	0.00000
Mitsuokella	0.03744	0.94613	0.10453
Neisseria	0.00000	0.00149	0.01100
Odoribacter	0.18040	0.20178	0.00092
Olsenella	0.00034	0.01794	0.04034
Oribacterium	0.00647	0.00598	0.24940

Genus	W_India_T1_7	W_India_T1_70	W_India_T1_71
**Oscillibacter**	0.05310	0.35424	0.00734
**Parabacteroides**	1.44625	0.37965	0.00092
**Paraprevotella**	0.47142	0.01196	0.00000
**Parasutterella**	0.00715	0.00149	0.00000
**Peptostreptococcus**	0.00068	0.00000	0.00367
**Prevotella**	19.92850	55.96230	74.73550
**Pseudoflavonifractor**	0.00102	0.00374	0.00092
**Pseudomonas**	0.00068	0.00149	0.00458
**Ralstonia**	0.00647	0.04185	0.00367
**Rhizobium**	0.00000	0.00000	0.00000
**Roseburia**	2.83463	2.96395	3.16150
**Rothia**	0.00102	0.00075	0.00275
**Ruminococcus**	0.78524	1.36987	0.12562
**Ruminococcus2**	0.11607	0.07025	0.06877
**Saccharibacteria_genera_incertae_sedis**	0.00374	0.01270	0.02109
**Slackia**	0.00000	0.00000	0.00000
**Solobacterium**	0.00170	0.00000	0.00000
**Streptococcus**	0.17257	0.07847	0.21364
**Streptophyta**	0.01702	0.00448	0.00183
**Succinivibrio**	0.00000	0.18609	2.94420
**Sutterella**	1.18858	0.56648	0.08619
**Turicibacter**	0.00885	0.03214	0.24665
**Veillonella**	0.00034	0.00598	0.04126
**Weissella**	0.00000	0.00000	0.00000

Genus	W_India_T1_72	W_India_T1_73	W_India_T1_74
Acinetobacter	0.00000	0.00288	0.00000
Actinomyces	0.00519	0.00460	0.00109
Akkermansia	0.00000	0.00000	0.00000
Alistipes	0.04797	0.01669	0.15748
Allisonella	0.00259	0.00000	0.00000
Alloprevotella	5.99546	0.00518	0.27450
Anaerostipes	0.00259	0.00173	0.00109
Bacteroides	0.12318	1.30639	0.52385
Barnesiella	0.00389	0.00000	0.00109
Bifidobacterium	0.18412	0.42587	0.92631
Bilophila	0.01037	0.00058	0.04921
Blautia	0.28007	0.18474	0.22857
Butyricicoccus	0.08687	0.01554	0.01969
Butyricimonas	0.22172	0.00115	0.00000
Campylobacter	0.00389	0.00000	0.00000
Catenibacterium	0.13744	0.00345	0.24935
Clostridium_IV	1.03468	0.00230	0.04375
Clostridium_sensu_stricto	0.14781	0.00576	0.05906
Clostridium_XI	0.09724	0.00921	0.01969
Clostridium_XlVa	0.14781	0.07712	0.12577
Clostridium_XlVb	0.06613	0.01266	0.02187
Collinsella	0.00000	0.01784	0.01312
Comamonas	0.00000	0.00000	0.00000
Coprococcus	0.30729	0.03971	1.77935
Dialister	0.17893	0.24401	0.78414
Dorea	0.33323	0.11683	0.39590
Elusimicrobium	0.00000	0.00000	0.00000
Enterobacter	0.00000	0.00288	0.00000
Erysipelotrichaceae_incertae_sedis	0.24506	0.00000	0.17061
Escherichia_Shigella	0.00389	0.46155	0.00984
Faecalibacterium	8.94003	19.82260	14.40320
Flavonifractor	0.00259	0.00230	0.00000
Fusicatenibacter	0.01297	0.02129	0.04593
Fusobacterium	0.00000	0.00230	0.00219
Gemella	0.00130	0.00115	0.00000
Gemmiger	0.00000	0.13006	0.42105
Granulicatella	0.00130	0.00230	0.00000
Haemophilus	0.01426	0.22732	0.05031
Hallella	0.00648	0.00000	0.00219
Hespellia	0.00389	0.00173	0.00000
Howardella	0.00908	0.00000	0.04921
Intestinimonas	0.02853	0.01093	0.10171
Klebsiella	0.00000	0.02014	0.00875
Lachnospiracea_incertae_sedis	0.22950	0.04949	0.24607
Lactobacillus	0.42658	0.01266	0.02953
Leptotrichia	0.00000	0.00115	0.00000
Megamonas	0.02982	1.49746	0.00000
Megasphaera	0.12577	0.05352	0.87710
Methylobacterium	0.00000	0.00000	0.00000
Mitsuokella	0.00000	0.00000	0.34668
Neisseria	0.00000	0.00518	0.00000
Odoribacter	0.00778	0.00230	0.07984
Olsenella	0.00130	0.00000	0.01312
Oribacterium	0.00130	0.00345	0.00437

Genus	W_India_T1_72	W_India_T1_73	W_India_T1_74
**Oscillibacter**	0.02334	0.01439	0.02515
**Parabacteroides**	0.07391	0.11337	0.40246
**Paraprevotella**	0.18023	0.00115	0.12468
**Parasutterella**	0.00259	0.00000	0.00000
**Peptostreptococcus**	0.00000	0.00000	0.00109
**Prevotella**	54.55820	72.54270	72.99590
**Pseudoflavonifractor**	0.00259	0.00000	0.00219
**Pseudomonas**	0.00000	0.00115	0.00109
**Ralstonia**	0.00000	0.00288	0.00000
**Rhizobium**	0.00000	0.00000	0.00000
**Roseburia**	1.37569	0.32228	3.35419
**Rothia**	0.00000	0.00345	0.00109
**Ruminococcus**	0.01426	0.07309	0.25701
**Ruminococcus2**	0.07002	0.11280	0.01640
**Saccharibacteria_genera_incertae_sedis**	0.00519	0.00115	0.00219
**Slackia**	0.00000	0.00000	0.00000
**Solobacterium**	0.00000	0.00000	0.00000
**Streptococcus**	0.03241	1.03763	0.08530
**Streptophyta**	0.00130	0.00058	0.00109
**Succinivibrio**	23.91700	0.00173	0.04046
**Sutterella**	0.18541	0.88052	0.11155
**Turicibacter**	0.00519	0.00115	0.00109
**Veillonella**	0.00259	0.03280	0.00219
**Weissella**	0.00130	0.00230	0.00000

Genus	W_India_T1_75	W_India_T1_76	W_India_T1_77
**Acinetobacter**	0.00000	0.00645	0.00000
**Actinomyces**	0.00145	0.00645	0.01400
**Akkermansia**	0.00000	0.00000	0.00000
**Alistipes**	0.15032	0.07463	0.01540
**Allisonella**	0.00073	0.01566	0.00420
**Alloprevotella**	0.09731	3.56205	0.16238
**Anaerostipes**	0.00000	0.00645	0.00700
**Bacteroides**	1.53152	1.06696	0.95607
**Barnesiella**	0.00073	0.00092	0.00000
**Bifidobacterium**	0.38488	0.63391	0.19597
**Bilophila**	0.00000	0.00553	0.00000
**Blautia**	0.10602	0.16769	0.07559
**Butyricicoccus**	0.04575	0.12899	0.03080
**Butyricimonas**	0.00000	0.01751	0.00000
**Campylobacter**	0.00073	0.00737	0.00700
**Catenibacterium**	0.03195	1.22083	0.13438
**Clostridium_IV**	0.00290	0.02303	0.01120
**Clostridium_sensu_stricto**	0.07770	0.34275	0.08259
**Clostridium_XI**	0.09586	0.37684	0.19457
**Clostridium_XlVa**	0.02832	0.19810	0.06579
**Clostridium_XlVb**	0.01089	0.03962	0.00840
**Collinsella**	0.06971	0.16124	0.00140
**Comamonas**	0.00000	0.00000	0.00000
**Coprococcus**	0.05156	1.15080	0.13298
**Dialister**	0.81260	1.66862	0.43254
**Dorea**	0.06318	0.35381	0.08959
**Elusimicrobium**	0.00000	0.00000	0.00000
**Enterobacter**	0.02760	0.05805	0.00420
**Erysipelotrichaceae_incertae_sedis**	0.00436	0.93612	0.05739
**Escherichia_Shigella**	0.24836	0.55006	0.31356
**Faecalibacterium**	9.19858	15.07100	7.37143
**Flavonifractor**	0.00073	0.00829	0.00140
**Fusicatenibacter**	0.01743	0.05344	0.02380
**Fusobacterium**	0.00145	0.02303	0.01680
**Gemella**	0.00073	0.00184	0.00420
**Gemmiger**	0.21713	0.44272	0.20437
**Granulicatella**	0.00073	0.00369	0.00280
**Haemophilus**	0.12127	0.81634	0.07979
**Hallella**	0.01598	0.01658	0.07279
**Hespellia**	0.00073	0.00000	0.00140
**Howardella**	0.00000	0.01751	0.00000
**Intestinimonas**	0.01525	0.05528	0.01120
**Klebsiella**	0.11256	0.85320	0.38215
**Lachnospiracea_incertae_sedis**	0.05809	0.19902	0.14278
**Lactobacillus**	0.52285	0.59153	0.05039
**Leptotrichia**	0.00363	0.01382	0.01820
**Megamonas**	0.08932	0.14558	0.07839
**Megasphaera**	0.10457	0.29116	0.01260
**Methylobacterium**	0.00000	0.00000	0.00000
**Mitsuokella**	0.00073	0.26259	0.00840
**Neisseria**	0.00290	0.02211	0.02380
**Odoribacter**	0.00000	0.02396	0.01680
**Olsenella**	0.00581	0.40172	0.00700
**Oribacterium**	0.00073	0.16769	0.01260

Genus	W_India_T1_75	W_India_T1_76	W_India_T1_77
**Oscillibacter**	0.01380	0.14051	0.04339
**Parabacteroides**	0.04938	0.21837	0.10499
**Paraprevotella**	0.00436	0.00369	0.00140
**Parasutterella**	0.00218	0.00737	0.01400
**Peptostreptococcus**	0.00871	0.00092	0.00000
**Prevotella**	82.36170	53.01610	84.96180
**Pseudoflavonifractor**	0.00000	0.00000	0.00280
**Pseudomonas**	0.00145	0.00276	0.00000
**Ralstonia**	0.00581	0.09122	0.01540
**Rhizobium**	0.00000	0.00000	0.00000
**Roseburia**	1.64844	7.14529	1.56639
**Rothia**	0.00944	0.04146	0.01680
**Ruminococcus**	0.08279	0.35289	0.09799
**Ruminococcus2**	0.03268	0.09582	0.01260
**Saccharibacteria_genera_incertae_sedis**	0.00726	0.01290	0.01260
**Slackia**	0.00000	0.00000	0.00000
**Solobacterium**	0.00000	0.00276	0.00000
**Streptococcus**	0.15686	1.54331	0.22537
**Streptophyta**	0.01598	0.02211	0.00140
**Succinivibrio**	0.01380	0.72973	0.04479
**Sutterella**	1.17715	2.87562	1.10305
**Turicibacter**	0.04430	0.52519	0.06719
**Veillonella**	0.00508	0.15019	0.04479
**Weissella**	0.00000	0.04238	0.00560

Genus	W_India_T1_78	W_India_T1_79	W_India_T1_8
Acinetobacter	0.00082	0.00157	0.00000
Actinomyces	0.00245	0.00626	0.00194
Akkermansia	0.00000	0.00000	0.00000
Alistipes	0.07430	0.24119	0.00194
Allisonella	0.00000	0.00157	0.00778
Alloprevotella	4.42724	3.35792	4.40649
Anaerostipes	0.00817	0.00000	0.00000
Bacteroides	0.07920	3.10733	0.04180
Barnesiella	0.00082	0.00000	0.00097
Bifidobacterium	0.46624	0.41348	0.80392
Bilophila	0.00000	0.04855	0.00778
Blautia	0.17311	0.34926	2.04139
Butyricicoccus	0.08655	0.11747	0.08166
Butyricimonas	0.00000	0.00000	0.00000
Campylobacter	0.00082	0.00783	0.00000
Catenibacterium	0.00980	0.00783	0.29454
Clostridium_IV	0.31845	0.27095	0.04374
Clostridium_sensu_stricto	1.95723	1.16838	0.45202
Clostridium_XI	0.61730	0.47926	0.28093
Clostridium_XlVa	0.08492	0.14409	0.11860
Clostridium_XlVb	0.01878	0.04699	0.02333
Collinsella	0.01306	0.17698	0.01750
Comamonas	0.00000	0.00000	0.00000
Coprococcus	0.10043	0.04542	0.55993
Dialister	0.00572	0.26939	1.79837
Dorea	0.06696	0.03602	0.43647
Elusimicrobium	0.00000	0.00000	0.00000
Enterobacter	0.76019	0.00626	0.00000
Erysipelotrichaceae_incertae_sedis	0.09880	0.06421	0.08263
Escherichia_Shigella	3.00811	2.33833	0.02527
Faecalibacterium	3.11589	12.54840	8.07322
Flavonifractor	0.02123	0.04229	0.00000
Fusicatenibacter	0.02695	0.01253	0.01555
Fusobacterium	0.00245	0.00313	0.00194
Gemella	0.00408	0.00940	0.00097
Gemmiger	0.15433	0.45263	0.07971
Granulicatella	0.00735	0.00940	0.00097
Haemophilus	2.70272	0.82225	0.08943
Hallella	0.02041	0.02506	0.01458
Hespellia	0.00000	0.00000	0.01264
Howardella	0.02123	0.00626	0.00000
Intestinimonas	0.01878	0.02663	0.03111
Klebsiella	8.01019	5.52397	0.00778
Lachnospiracea_incertae_sedis	0.15269	0.12373	0.55409
Lactobacillus	0.07104	0.05638	0.21580
Leptotrichia	0.00408	0.00626	0.00389
Megamonas	0.61403	0.48709	0.00000
Megasphaera	0.00082	0.14566	0.61047
Methylobacterium	0.00000	0.00000	0.00000
Mitsuokella	0.00163	0.15975	2.59354
Neisseria	0.00490	0.00470	0.01458
Odoribacter	0.00000	0.08301	0.00583
Olsenella	0.00163	0.00313	0.01069
Oribacterium	0.00163	0.00000	0.00486

Genus	W_India_T1_78	W_India_T1_79	W_India_T1_8
**Oscillibacter**	0.07839	0.19734	0.03013
**Parabacteroides**	0.12411	0.42287	0.01653
**Paraprevotella**	0.00163	0.00000	0.00292
**Parasutterella**	0.00082	0.00313	0.00000
**Peptostreptococcus**	0.00245	0.00157	0.00194
**Prevotella**	67.17130	41.08440	65.42270
**Pseudoflavonifractor**	0.00163	0.00000	0.00292
**Pseudomonas**	0.00000	0.00000	0.00486
**Ralstonia**	0.04001	0.06578	0.00389
**Rhizobium**	0.00000	0.00000	0.00000
**Roseburia**	1.31054	0.94285	3.75130
**Rothia**	0.01960	0.02193	0.00292
**Ruminococcus**	0.03756	2.35712	0.84572
**Ruminococcus2**	0.00653	0.12060	0.05638
**Saccharibacteria_genera_incertae_sedis**	0.00245	0.00470	0.01458
**Slackia**	0.00000	0.00000	0.00000
**Solobacterium**	0.00000	0.00157	0.00000
**Streptococcus**	0.57239	0.36179	0.19053
**Streptophyta**	0.06532	0.04542	0.01361
**Succinivibrio**	0.01551	18.34330	0.00194
**Sutterella**	0.01225	0.03915	0.31107
**Turicibacter**	2.54595	1.87474	0.02041
**Veillonella**	0.03103	0.02976	0.02722
**Weissella**	0.00490	0.01253	0.00097

Genus	W_India_T1_80	W_India_T1_9	W_India_T60_1
**Acinetobacter**	0.00353	0.00000	0.00000
**Actinomyces**	0.00235	0.00768	0.00271
**Akkermansia**	0.00000	0.00000	0.00000
**Alistipes**	0.01176	0.00000	0.01718
**Allisonella**	0.00353	0.00000	0.00271
**Alloprevotella**	0.01411	0.01151	1.86195
**Anaerostipes**	0.00118	0.00256	0.00723
**Bacteroides**	0.75595	3.25977	0.12841
**Barnesiella**	0.00000	0.00000	0.00090
**Bifidobacterium**	0.25629	12.78960	0.04974
**Bilophila**	0.00000	0.00000	0.00452
**Blautia**	0.08465	0.72411	0.14017
**Butyricicoccus**	0.02939	0.05629	0.04612
**Butyricimonas**	0.00000	0.00000	0.01447
**Campylobacter**	0.00118	0.00128	0.00723
**Catenibacterium**	0.19633	0.00000	0.13655
**Clostridium_IV**	0.01176	0.00000	0.01989
**Clostridium_sensu_stricto**	0.08347	0.29297	0.22155
**Clostridium_XI**	0.49142	0.38252	0.12118
**Clostridium_XlVa**	0.08465	0.16887	0.10580
**Clostridium_XlVb**	0.01528	0.00384	0.02532
**Collinsella**	0.00470	2.52031	0.03798
**Comamonas**	0.00000	0.00000	0.03255
**Coprococcus**	0.09053	0.05373	1.10777
**Dialister**	0.42794	0.00000	0.14831
**Dorea**	0.09288	1.01196	0.13203
**Elusimicrobium**	0.00000	0.00000	0.00000
**Enterobacter**	0.00000	0.00000	0.00723
**Erysipelotrichaceae_incertae_sedis**	0.02234	0.00000	0.15102
**Escherichia_Shigella**	0.23983	2.92330	0.03617
**Faecalibacterium**	14.44170	45.52290	7.36551
**Flavonifractor**	0.00000	0.01151	0.00000
**Fusicatenibacter**	0.03292	0.88403	0.00271
**Fusobacterium**	0.00353	0.00000	0.00362
**Gemella**	0.00118	0.00000	0.00181
**Gemmiger**	0.33506	2.08021	0.15825
**Granulicatella**	0.00235	0.03326	0.00271
**Haemophilus**	0.10463	1.22945	0.27943
**Hallella**	0.01058	0.00000	0.00543
**Hespellia**	0.00000	0.01279	0.00181
**Howardella**	0.00000	0.00000	0.00543
**Intestinimonas**	0.02469	0.00000	0.03075
**Klebsiella**	0.52905	0.00384	0.02351
**Lachnospiracea_incertae_sedis**	0.01528	1.60174	0.18538
**Lactobacillus**	0.03762	3.01797	1.04266
**Leptotrichia**	0.00588	0.00000	0.00271
**Megamonas**	0.05173	0.00000	0.00000
**Megasphaera**	0.00235	0.00256	0.17001
**Methylobacterium**	0.00000	0.00000	0.00271
**Mitsuokella**	0.00235	0.00640	0.18176
**Neisseria**	0.00705	0.00128	0.00814
**Odoribacter**	0.00823	0.00000	0.01718
**Olsenella**	0.00000	0.00000	0.00633
**Oribacterium**	0.00000	0.00256	0.00090

Genus	W_India_T1_80	W_India_T1_9	W_India_T60_1
**Oscillibacter**	0.04115	0.00000	0.05426
**Parabacteroides**	0.09993	0.00000	0.05064
**Paraprevotella**	0.00118	0.00000	0.01176
**Parasutterella**	0.00705	0.00000	0.00362
**Peptostreptococcus**	0.00000	0.01663	0.00090
**Prevotella**	78.36210	0.27122	65.17190
**Pseudoflavonifractor**	0.00000	0.00000	0.00543
**Pseudomonas**	0.00000	0.00000	0.00000
**Ralstonia**	0.00470	0.01279	0.00000
**Rhizobium**	0.00000	0.00000	0.00181
**Roseburia**	1.33437	6.24576	2.44341
**Rothia**	0.00470	0.01791	0.00633
**Ruminococcus**	0.13520	0.00384	0.40151
**Ruminococcus2**	0.01528	0.00896	0.07506
**Saccharibacteria_genera_incertae_sedis**	0.00235	0.02687	0.00090
**Slackia**	0.00000	0.00000	0.00181
**Solobacterium**	0.00000	0.00128	0.00000
**Streptococcus**	0.09053	1.53010	0.20075
**Streptophyta**	0.00588	0.00640	0.00452
**Succinivibrio**	0.01411	0.00384	16.47090
**Sutterella**	1.23679	8.92727	0.73339
**Turicibacter**	0.06701	0.12154	0.09586
**Veillonella**	0.00118	2.11092	0.12389
**Weissella**	0.00118	0.00000	0.00362

Genus	W_India_T60_10	W_India_T60_11	W_India_T60_12
Acinetobacter	0.00546	0.00000	0.00246
Actinomyces	0.00000	0.00287	0.00197
Akkermansia	0.00000	0.00000	0.00443
Alistipes	0.22322	0.07316	0.08023
Allisonella	0.00055	0.00000	0.00049
Alloprevotella	0.01092	7.14127	5.80430
Anaerostipes	0.00055	0.00143	0.00000
Bacteroides	20.69510	0.31270	5.12460
Barnesiella	0.00000	0.00143	0.00000
Bifidobacterium	0.98239	0.07244	0.52910
Bilophila	0.01637	0.02582	0.00394
Blautia	0.29253	0.16281	0.11468
Butyricicoccus	0.05621	0.05236	0.05463
Butyricimonas	0.16155	0.00072	0.04085
Campylobacter	0.00109	0.00072	0.00295
Catenibacterium	0.00000	0.30482	0.15947
Clostridium_IV	0.01692	0.01506	0.00689
Clostridium_sensu_stricto	0.02565	0.22664	0.22099
Clostridium_XI	0.01255	0.06527	0.11566
Clostridium_XlVa	0.19975	0.06240	0.18457
Clostridium_XlVb	0.03384	0.03658	0.02559
Collinsella	0.00055	0.02295	0.02116
Comamonas	0.00164	0.00215	0.00000
Coprococcus	0.27179	0.58668	0.18949
Dialister	0.12171	0.15994	0.25151
Dorea	0.03766	0.11547	0.04725
Elusimicrobium	0.00000	0.00000	0.00049
Enterobacter	0.00327	0.00000	0.00049
Erysipelotrichaceae_incertae_sedis	0.00055	0.03873	0.01427
Escherichia_Shigella	0.00055	0.00574	0.13486
Faecalibacterium	12.20180	11.24800	10.22510
Flavonifractor	0.00491	0.00000	0.00295
Fusicatenibacter	0.08514	0.01291	0.03150
Fusobacterium	0.00000	0.00000	0.00098
Gemella	0.00000	0.00072	0.00246
Gemmiger	0.63419	0.14990	0.23182
Granulicatella	0.00000	0.00000	0.00049
Haemophilus	0.29690	0.26537	0.23674
Hallella	0.00055	0.00143	0.00049
Hespellia	0.00873	0.00215	0.00098
Howardella	0.00000	0.00645	0.00935
Intestinimonas	0.04748	0.02869	0.03544
Klebsiella	0.07586	0.00000	0.04134
Lachnospiracea_incertae_sedis	0.35421	0.33494	0.23083
Lactobacillus	0.02347	0.04734	0.04233
Leptotrichia	0.00382	0.00072	0.00148
Megamonas	0.00055	0.00430	0.06546
Megasphaera	0.16046	0.07602	0.07530
Methylobacterium	0.01474	0.00072	0.00541
Mitsuokella	0.13753	0.06598	0.31500
Neisseria	0.01092	0.00359	0.00345
Odoribacter	0.18338	0.00072	0.06546
Olsenella	0.00055	0.00861	0.00295
Oribacterium	0.00000	0.00072	0.00197

Genus	W_India_T60_10	W_India_T60_11	W_India_T60_12
**Oscillibacter**	0.04202	0.05092	0.02461
**Parabacteroides**	0.63200	0.11691	0.20918
**Paraprevotella**	0.54686	0.00000	0.14273
**Parasutterella**	0.03384	0.00000	0.00935
**Peptostreptococcus**	0.00055	0.00000	0.00148
**Prevotella**	51.10980	65.49640	68.23750
**Pseudoflavonifractor**	0.00000	0.00359	0.00098
**Pseudomonas**	0.00000	0.00000	0.00000
**Ralstonia**	0.00000	0.00000	0.00000
**Rhizobium**	0.00055	0.00000	0.00049
**Roseburia**	7.10048	1.52622	4.28493
**Rothia**	0.00164	0.01004	0.00148
**Ruminococcus**	0.33892	0.09467	0.18851
**Ruminococcus2**	0.23086	0.04016	0.07481
**Saccharibacteria_genera_incertae_sedis**	0.00000	0.00000	0.00295
**Slackia**	0.00000	0.00000	0.00000
**Solobacterium**	0.00000	0.00000	0.00000
**Streptococcus**	1.07517	0.32418	0.35585
**Streptophyta**	0.01965	0.00789	0.01132
**Succinivibrio**	0.01146	10.00080	0.00738
**Sutterella**	1.09755	0.42746	0.71170
**Turicibacter**	0.22104	0.00072	0.71268
**Veillonella**	0.06276	0.11619	0.19589
**Weissella**	0.00000	0.00000	0.00000

Genus	W_India_T60_13	W_India_T60_14	W_India_T60_15
Acinetobacter	0.00000	0.00000	0.00000
Actinomyces	0.00072	0.00917	0.00915
Akkermansia	0.01515	0.00000	0.00458
Alistipes	0.12266	0.10874	0.19527
Allisonella	0.00000	0.00262	0.01373
Alloprevotella	0.03247	1.17777	3.72986
Anaerostipes	0.00505	0.01179	0.00076
Bacteroides	1.04119	1.18170	9.05541
Barnesiella	0.53899	0.05764	0.61249
Bifidobacterium	0.25615	2.28086	3.67800
Bilophila	0.00072	0.04585	0.00000
Blautia	0.13854	0.42185	0.29824
Butyricicoccus	0.16090	0.10088	0.20137
Butyricimonas	0.00000	0.22402	0.00153
Campylobacter	0.00000	0.00524	0.03356
Catenibacterium	0.91059	0.55810	0.30815
Clostridium_IV	0.06855	2.27955	0.00153
Clostridium_sensu_stricto	0.06277	0.37206	2.06248
Clostridium_XI	0.18832	0.04585	0.66054
Clostridium_XlVa	0.35716	0.57644	0.13119
Clostridium_XlVb	0.02020	0.12184	0.04119
Collinsella	0.05267	0.14411	1.56593
Comamonas	0.00000	0.05109	0.00458
Coprococcus	0.19626	1.20397	0.40960
Dialister	0.10967	1.43978	0.58961
Dorea	0.06927	0.40613	0.23569
Elusimicrobium	0.00000	0.00000	0.00000
Enterobacter	0.00866	0.00655	0.00305
Erysipelotrichaceae_incertae_sedis	0.00216	0.29215	0.00534
Escherichia_Shigella	0.06710	0.03144	0.14950
Faecalibacterium	10.76830	15.16290	12.17280
Flavonifractor	0.00072	0.23713	0.00229
Fusicatenibacter	0.02165	0.08516	0.24561
Fusobacterium	0.00000	0.01703	0.22806
Gemella	0.00216	0.00393	0.03814
Gemmiger	0.14575	0.53320	1.23871
Granulicatella	0.00216	0.01441	0.04119
Haemophilus	0.19915	0.06026	6.45594
Hallella	0.00000	0.01441	0.00000
Hespellia	0.00000	0.00786	0.00153
Howardella	0.00000	0.02358	0.00153
Intestinimonas	0.04762	0.33276	0.01831
Klebsiella	0.13060	0.00524	2.88778
Lachnospiracea_incertae_sedis	0.15946	0.94195	0.24332
Lactobacillus	0.34490	0.12970	3.04339
Leptotrichia	0.00000	0.00000	0.00305
Megamonas	0.04113	0.00262	0.14035
Megasphaera	0.00216	0.66683	0.34019
Methylobacterium	0.00144	0.00131	0.07322
Mitsuokella	0.15513	0.46639	0.28146
Neisseria	0.00000	0.02358	0.02365
Odoribacter	0.00000	0.08254	0.02898
Olsenella	0.00072	0.03799	0.36002
Oribacterium	0.00216	0.00262	0.02441

Genus	W_India_T60_13	W_India_T60_14	W_India_T60_15
**Oscillibacter**	0.11400	0.60002	0.01220
**Parabacteroides**	0.39973	3.24508	1.41948
**Paraprevotella**	0.00000	0.12839	0.03890
**Parasutterella**	0.00000	0.00393	0.00763
**Peptostreptococcus**	0.00072	0.00393	0.07780
**Prevotella**	78.99370	46.81060	26.87330
**Pseudoflavonifractor**	0.00000	0.03144	0.00000
**Pseudomonas**	0.00000	0.00000	0.00000
**Ralstonia**	0.00000	0.00000	0.00000
**Rhizobium**	0.00000	0.00000	0.00458
**Roseburia**	2.23822	0.69041	6.50247
**Rothia**	0.00649	0.04454	0.07551
**Ruminococcus**	0.03896	1.91534	0.39282
**Ruminococcus2**	0.05051	0.13363	0.10755
**Saccharibacteria_genera_incertae_sedis**	0.00649	0.00262	0.06560
**Slackia**	0.00144	0.01048	0.00305
**Solobacterium**	0.00000	0.00000	0.00000
**Streptococcus**	0.44447	0.17031	4.71458
**Streptophyta**	0.01082	0.00000	0.20747
**Succinivibrio**	0.00649	13.94720	0.03509
**Sutterella**	1.12488	0.01441	3.57655
**Turicibacter**	0.05484	0.03275	0.50494
**Veillonella**	0.02309	0.02227	3.06398
**Weissella**	0.00072	0.00000	0.02898

Genus	W_India_T60_16	W_India_T60_17	W_India_T60_18
**Acinetobacter**	0.00282	0.00000	0.00498
**Actinomyces**	0.00635	0.00120	0.00711
**Akkermansia**	0.00071	0.00000	0.00356
**Alistipes**	0.07061	0.30448	0.00213
**Allisonella**	0.01130	0.00602	0.04267
**Alloprevotella**	3.49018	0.01023	0.02133
**Anaerostipes**	0.00000	0.00060	0.02418
**Bacteroides**	0.06425	12.88380	0.47856
**Barnesiella**	0.00706	0.00060	0.00000
**Bifidobacterium**	3.04817	0.16127	1.41152
**Bilophila**	0.01200	0.11914	0.00071
**Blautia**	0.27608	0.07883	0.69687
**Butyricicoccus**	0.06496	0.16548	0.17635
**Butyricimonas**	0.00071	0.08424	0.00000
**Campylobacter**	0.00282	0.00060	0.00284
**Catenibacterium**	2.38869	0.15525	1.99603
**Clostridium_IV**	0.06920	0.00542	0.00071
**Clostridium_sensu_stricto**	0.01130	0.00120	0.00356
**Clostridium_XI**	0.00353	0.00120	0.00711
**Clostridium_XlVa**	0.16522	1.95022	0.26950
**Clostridium_XlVb**	0.03813	0.03370	0.02489
**Collinsella**	0.32268	0.07161	0.67696
**Comamonas**	0.00071	0.00000	0.00000
**Coprococcus**	0.80847	0.34359	0.19626
**Dialister**	0.89532	0.50245	0.22968
**Dorea**	0.46955	0.06499	0.61225
**Elusimicrobium**	0.00000	0.00120	0.00071
**Enterobacter**	0.01271	0.00000	0.00142
**Erysipelotrichaceae_incertae_sedis**	0.07626	0.18112	0.00142
**Escherichia_Shigella**	0.19912	1.69930	0.20337
**Faecalibacterium**	17.28000	5.14363	13.29880
**Flavonifractor**	0.00000	0.00542	0.00071
**Fusicatenibacter**	0.03742	0.02467	0.02773
**Fusobacterium**	0.01553	0.00301	0.00427
**Gemella**	0.00777	0.00722	0.00142
**Gemmiger**	0.32198	0.14562	0.41030
**Granulicatella**	0.03036	0.00662	0.00284
**Haemophilus**	0.26337	0.06679	2.69290
**Hallella**	0.04307	0.00000	0.00569
**Hespellia**	0.00706	0.00000	0.00000
**Howardella**	0.00000	0.00241	0.00000
**Intestinimonas**	0.06849	0.03370	0.02347
**Klebsiella**	0.10238	0.00481	0.01067
**Lachnospiracea_incertae_sedis**	0.29585	0.26296	1.46058
**Lactobacillus**	0.82824	0.02467	2.04154
**Leptotrichia**	0.00424	0.00000	0.06684
**Megamonas**	0.00353	0.00120	0.90664
**Megasphaera**	0.98005	0.36225	0.19982
**Methylobacterium**	0.00212	0.00000	0.10098
**Mitsuokella**	0.69902	0.00241	0.00213
**Neisseria**	0.02330	0.01745	0.27875
**Odoribacter**	0.00141	0.12396	0.00071
**Olsenella**	0.03742	0.00421	0.00142
**Oribacterium**	0.00353	0.00060	0.01564

Genus	W_India_T60_16	W_India_T60_17	W_India_T60_18
**Oscillibacter**	0.12357	0.08665	0.02133
**Parabacteroides**	0.01977	0.61919	0.02631
**Paraprevotella**	0.00424	0.09808	0.00071
**Parasutterella**	0.00000	1.52660	0.00284
**Peptostreptococcus**	0.00141	0.00060	0.00000
**Prevotella**	46.25490	67.05680	54.88910
**Pseudoflavonifractor**	0.06496	0.00000	0.00000
**Pseudomonas**	0.00000	0.00000	0.00000
**Ralstonia**	0.00000	0.00000	0.00000
**Rhizobium**	0.00071	0.00060	0.00640
**Roseburia**	6.39078	3.69466	7.18557
**Rothia**	0.02048	0.01143	0.06400
**Ruminococcus**	0.10450	0.13900	0.02347
**Ruminococcus2**	0.05084	0.01264	0.17066
**Saccharibacteria_genera_incertae_sedis**	0.00706	0.00301	0.02347
**Slackia**	0.00635	0.00000	0.01991
**Solobacterium**	0.00000	0.00000	0.00000
**Streptococcus**	0.59594	0.02708	2.59050
**Streptophyta**	0.00353	0.00060	0.04338
**Succinivibrio**	0.04166	0.02467	0.01351
**Sutterella**	1.65295	0.00662	5.40074
**Turicibacter**	0.03107	0.10952	0.00996
**Veillonella**	0.13839	0.01926	0.19911
**Weissella**	0.00000	0.00060	0.00000

Genus	W_India_T60_19	W_India_T60_2	W_India_T60_20
Acinetobacter	0.00000	0.00922	0.00021
Actinomyces	0.00041	0.00461	0.00021
Akkermansia	0.00000	0.00154	0.00000
Alistipes	0.00000	0.04765	0.01131
Allisonella	0.00000	0.00922	0.00165
Alloprevotella	6.83932	7.15834	6.92893
Anaerostipes	0.00447	0.00461	0.00082
Bacteroides	0.91310	0.58872	0.07898
Barnesiella	0.00000	0.00154	0.00041
Bifidobacterium	0.39522	1.00527	0.53043
Bilophila	0.00162	0.00000	0.00247
Blautia	0.27052	0.32587	0.14089
Butyricicoccus	0.01584	0.14756	0.03599
Butyricimonas	0.38019	0.00615	0.00000
Campylobacter	0.00000	0.00154	0.00000
Catenibacterium	0.78597	0.40734	0.47243
Clostridium_IV	0.14663	0.00922	0.00185
Clostridium_sensu_stricto	0.50448	0.08300	0.02797
Clostridium_XI	0.12957	0.04765	0.01501
Clostridium_XlVa	0.29408	0.16908	0.19539
Clostridium_XlVb	0.01259	0.03843	0.01501
Collinsella	0.01219	0.03689	0.02571
Comamonas	0.00000	0.01383	0.00555
Coprococcus	0.00122	0.07532	0.23920
Dialister	0.52926	1.46487	0.18881
Dorea	0.00000	0.14910	0.08844
Elusimicrobium	0.00000	0.02613	0.00000
Enterobacter	0.00203	0.00000	0.00062
Erysipelotrichaceae_incertae_sedis	0.30586	0.04304	0.03558
Escherichia_Shigella	0.96834	0.31050	0.00864
Faecalibacterium	8.39379	13.58350	5.13299
Flavonifractor	0.05159	0.00000	0.00041
Fusicatenibacter	0.08286	0.06148	0.06767
Fusobacterium	0.00081	0.00154	0.00021
Gemella	0.00122	0.00154	0.00000
Gemmiger	0.27458	0.30896	0.11929
Granulicatella	0.00325	0.00769	0.00062
Haemophilus	0.34201	0.65020	0.00123
Hallella	0.00081	0.00000	0.00000
Hespellia	0.00041	0.00000	0.00226
Howardella	0.00000	0.02152	0.00350
Intestinimonas	0.04387	0.00615	0.02036
Klebsiella	0.00691	0.16140	0.00494
Lachnospiracea_incertae_sedis	0.29489	0.39658	0.25236
Lactobacillus	0.60684	0.53338	0.21966
Leptotrichia	0.00041	0.00461	0.00000
Megamonas	0.68158	0.02459	0.00000
Megasphaera	1.79574	0.01691	0.01460
Methylobacterium	0.00081	0.00154	0.00514
Mitsuokella	0.00081	0.00922	0.07672
Neisseria	0.00041	0.00461	0.00041
Odoribacter	0.00000	0.00769	0.00699
Olsenella	0.01015	0.00461	0.00309
Oribacterium	0.00041	0.00461	0.00350

Genus	W_India_T60_19	W_India_T60_2	W_India_T60_20
**Oscillibacter**	0.15313	0.06456	0.01378
**Parabacteroides**	0.47442	0.14141	0.06067
**Paraprevotella**	0.42527	0.05995	0.01028
**Parasutterella**	0.00731	0.00615	0.00000
**Peptostreptococcus**	0.00162	0.00000	0.00123
**Prevotella**	70.48550	66.44330	61.48540
**Pseudoflavonifractor**	0.00284	0.00000	0.00103
**Pseudomonas**	0.00000	0.00000	0.00000
**Ralstonia**	0.00000	0.00000	0.00000
**Rhizobium**	0.00041	0.00000	0.00000
**Roseburia**	0.53454	1.34036	3.36872
**Rothia**	0.00284	0.01537	0.00000
**Ruminococcus**	0.51585	0.04304	0.01152
**Ruminococcus2**	0.00650	0.02306	0.04052
**Saccharibacteria_genera_incertae_sedis**	0.00528	0.00307	0.00041
**Slackia**	0.00000	0.00154	0.00185
**Solobacterium**	0.00000	0.00000	0.00000
**Streptococcus**	0.03859	1.31270	0.00309
**Streptophyta**	0.00081	0.06302	0.00062
**Succinivibrio**	0.00122	0.03228	19.61490
**Sutterella**	2.16130	2.03668	0.37679
**Turicibacter**	0.01990	0.00461	0.00926
**Veillonella**	0.01787	0.12604	0.00041
**Weissella**	0.00041	0.00000	0.00021

Genus	W_India_T60_21	W_India_T60_22	W_India_T60_23
Acinetobacter	0.00018	0.00055	0.00055
Actinomyces	0.00165	0.00248	0.00109
Akkermansia	0.00018	0.01021	0.00055
Alistipes	0.07095	0.64856	0.25929
Allisonella	0.00000	0.00000	0.00055
Alloprevotella	0.01415	0.00966	13.73680
Anaerostipes	0.00018	0.04995	0.00109
Bacteroides	0.36356	2.81033	1.23135
Barnesiella	0.09705	0.97339	0.00274
Bifidobacterium	0.63007	0.28537	0.33806
Bilophila	0.00496	0.07148	0.03118
Blautia	0.09411	0.22796	0.14606
Butyricicoccus	0.02077	0.11702	0.07549
Butyricimonas	0.00000	0.00000	0.00000
Campylobacter	0.00000	0.00000	0.00000
Catenibacterium	0.14098	0.04140	0.00274
Clostridium_IV	0.04062	0.50063	0.00164
Clostridium_sensu_stricto	0.02481	0.03643	0.01532
Clostridium_XI	0.02518	0.00469	0.02352
Clostridium_XlVa	0.11267	0.12585	0.26367
Clostridium_XlVb	0.00643	0.02180	0.01860
Collinsella	0.00460	0.06982	0.01586
Comamonas	0.00000	0.00000	0.01422
Coprococcus	0.12995	0.89363	0.92884
Dialister	0.58467	1.86012	0.31891
Dorea	0.06488	0.49677	0.13949
Elusimicrobium	0.00037	0.00000	0.00000
Enterobacter	0.00680	0.00000	0.00438
Erysipelotrichaceae_incertae_sedis	0.00037	0.42419	0.03446
Escherichia_Shigella	0.04374	0.17939	2.07157
Faecalibacterium	10.06560	20.59630	8.47994
Flavonifractor	0.00092	0.01656	0.00000
Fusicatenibacter	0.01176	0.05106	0.01094
Fusobacterium	0.00037	0.00083	0.00000
Gemella	0.00074	0.00110	0.00055
Gemmiger	0.08583	0.45758	0.24890
Granulicatella	0.00055	0.00248	0.00000
Haemophilus	0.06396	0.02373	0.07494
Hallella	0.00000	0.00221	0.01149
Hespellia	0.00184	0.00580	0.00328
Howardella	0.00000	0.00000	0.01039
Intestinimonas	0.00919	0.05354	0.04103
Klebsiella	21.08090	0.00442	5.00470
Lachnospiracea_incertae_sedis	0.06359	0.76889	0.27461
Lactobacillus	0.09190	0.25308	0.16958
Leptotrichia	0.00000	0.00055	0.00000
Megamonas	0.00000	0.00083	0.00109
Megasphaera	0.49497	0.81304	0.15317
Methylobacterium	0.00239	0.00276	0.00109
Mitsuokella	4.18053	0.47911	0.16739
Neisseria	0.00000	0.00331	0.00821
Odoribacter	0.01434	0.06348	0.17450
Olsenella	0.01434	0.03146	0.00383
Oribacterium	0.00368	0.00552	0.00602

Genus	W_India_T60_21	W_India_T60_22	W_India_T60_23
Oscillibacter	0.02849	0.07176	0.04814
Parabacteroides	0.12112	0.19540	0.21826
Paraprevotella	0.00790	0.00000	0.09737
Parasutterella	0.00607	0.03201	0.01969
Peptostreptococcus	0.00000	0.00000	0.00000
Prevotella	58.54690	59.76230	60.51920
Pseudoflavonifractor	0.00000	0.00083	0.01149
Pseudomonas	0.00000	0.00000	0.00000
Ralstonia	0.00000	0.00000	0.00000
Rhizobium	0.00018	0.00028	0.00055
Roseburia	1.37611	3.70644	3.86307
Rothia	0.00239	0.00635	0.00055
Ruminococcus	0.01305	1.71192	0.24671
Ruminococcus2	0.01195	0.10957	0.01203
Saccharibacteria_genera_incertae_sedis	0.00092	0.00414	0.00219
Slackia	0.00110	0.02263	0.00109
Solobacterium	0.00000	0.00000	0.00000
Streptococcus	0.63264	0.03063	0.14606
Streptophyta	0.00110	0.00110	0.00109
Succinivibrio	0.01985	0.01380	0.01860
Sutterella	0.43285	0.78213	0.00383
Turicibacter	0.09778	0.00110	0.09682
Veillonella	0.00386	0.00607	0.00164
Weissella	0.00000	0.00000	0.00164

Genus	W_India_T60_24	W_India_T60_25	W_India_T60_26
**Acinetobacter**	0.00089	0.00000	0.00051
**Actinomyces**	0.00089	0.00346	0.01067
**Akkermansia**	0.00000	0.00077	0.00000
**Alistipes**	0.02590	0.00038	0.00711
**Allisonella**	0.00000	0.00000	0.00000
**Alloprevotella**	10.33410	1.34406	0.00356
**Anaerostipes**	0.00179	0.16671	0.00051
**Bacteroides**	0.06521	0.00423	0.23977
**Barnesiella**	0.00089	0.00000	0.00000
**Bifidobacterium**	0.32872	1.38170	0.60349
**Bilophila**	0.00893	0.04533	0.00305
**Blautia**	0.20277	0.21626	0.26517
**Butyricicoccus**	0.04734	0.06108	0.67004
**Butyricimonas**	0.03752	0.00000	0.00356
**Campylobacter**	0.00179	0.00269	0.00559
**Catenibacterium**	0.96740	3.35726	0.00457
**Clostridium_IV**	0.04288	0.31037	0.00406
**Clostridium_sensu_stricto**	0.11434	0.04187	1.14958
**Clostridium_XI**	0.13488	0.02190	0.55777
**Clostridium_XlVa**	0.19384	0.08143	0.03556
**Clostridium_XlVb**	0.02233	0.05647	0.00254
**Collinsella**	0.01608	0.03303	0.00203
**Comamonas**	0.00000	0.00230	0.00000
**Coprococcus**	0.34122	1.28452	0.00051
**Dialister**	0.32604	0.60462	0.45973
**Dorea**	0.14203	0.08720	0.15087
**Elusimicrobium**	0.00089	3.60272	0.00102
**Enterobacter**	0.00536	0.00077	0.01981
**Erysipelotrichaceae_incertae_sedis**	0.08665	0.06223	0.00152
**Escherichia_Shigella**	0.05628	0.00461	0.05791
**Faecalibacterium**	11.46490	11.61940	5.86932
**Flavonifractor**	0.00089	0.15442	0.01422
**Fusicatenibacter**	0.01429	0.01191	0.04928
**Fusobacterium**	0.00983	0.00154	0.00203
**Gemella**	0.00089	0.00154	0.00051
**Gemmiger**	0.19920	0.14866	0.12497
**Granulicatella**	0.00268	0.00154	0.00508
**Haemophilus**	0.68691	0.11716	3.88664
**Hallella**	0.00000	0.02535	0.00711
**Hespellia**	0.00089	0.00115	0.00102
**Howardella**	0.00804	0.08143	0.02489
**Intestinimonas**	0.05092	0.04610	0.01473
**Klebsiella**	0.41090	0.00999	1.72768
**Lachnospiracea_incertae_sedis**	0.26351	0.13982	0.33223
**Lactobacillus**	0.55203	0.05147	4.38701
**Leptotrichia**	0.00000	0.00077	0.00254
**Megamonas**	0.05628	0.00000	0.00000
**Megasphaera**	0.00089	0.30000	2.28037
**Methylobacterium**	0.00625	0.00115	0.00051
**Mitsuokella**	0.64672	0.35148	0.00203
**Neisseria**	0.00804	0.02766	0.04420
**Odoribacter**	0.07235	0.00000	0.00000
**Olsenella**	0.01340	0.05685	0.03404
**Oribacterium**	0.00000	0.00269	0.00660

Genus	W_India_T60_24	W_India_T60_25	W_India_T60_26
**Oscillibacter**	0.07861	0.10064	0.00102
**Parabacteroides**	0.00983	0.01229	0.02896
**Paraprevotella**	0.02680	0.01882	0.00051
**Parasutterella**	0.00000	0.00000	0.00051
**Peptostreptococcus**	0.00000	0.00077	0.00965
**Prevotella**	68.91920	45.12100	66.82870
**Pseudoflavonifractor**	0.00447	0.00691	0.00000
**Pseudomonas**	0.00000	0.00000	0.00000
**Ralstonia**	0.00000	0.00000	0.00000
**Rhizobium**	0.00089	0.00038	0.00051
**Roseburia**	1.45422	2.54522	0.68680
**Rothia**	0.00447	0.04763	0.02083
**Ruminococcus**	0.03037	0.51627	0.00203
**Ruminococcus2**	0.08843	0.01344	0.01067
**Saccharibacteria_genera_incertae_sedis**	0.00089	0.00576	0.01676
**Slackia**	0.00536	0.00461	0.00000
**Solobacterium**	0.00000	0.00000	0.00000
**Streptococcus**	0.13667	0.09257	5.53100
**Streptophyta**	0.00536	0.00423	0.00813
**Succinivibrio**	0.01429	24.56600	0.01829
**Sutterella**	0.62975	0.56620	2.07159
**Turicibacter**	0.05717	0.00269	0.77977
**Veillonella**	0.01608	0.03957	0.64058
**Weissella**	0.00000	0.00000	0.18847

Genus	W_India_T60_27	W_India_T60_28	W_India_T60_29
**Acinetobacter**	0.00000	0.00120	0.05549
**Actinomyces**	0.00192	0.00440	0.00145
**Akkermansia**	0.00823	0.69239	0.00087
**Alistipes**	0.04935	3.16016	0.00636
**Allisonella**	0.00082	0.01401	0.00087
**Alloprevotella**	4.43780	0.02121	9.41284
**Anaerostipes**	0.00027	0.00480	0.00607
**Bacteroides**	0.63499	37.97670	0.09191
**Barnesiella**	0.00137	0.01241	0.00000
**Bifidobacterium**	0.47213	2.77274	0.17515
**Bilophila**	0.00576	0.02041	0.00318
**Blautia**	0.22729	0.28136	0.10087
**Butyricicoccus**	0.07458	0.04723	0.06503
**Butyricimonas**	0.02001	0.02842	0.00000
**Campylobacter**	0.00000	0.00000	0.00000
**Catenibacterium**	0.20289	0.22453	0.00116
**Clostridium_IV**	0.09815	0.01961	0.00434
**Clostridium_sensu_stricto**	0.19439	0.06123	0.75639
**Clostridium_XI**	0.02193	0.12447	0.39828
**Clostridium_XlVa**	0.19521	0.27816	0.10174
**Clostridium_XlVb**	0.02851	0.03282	0.00405
**Collinsella**	0.00356	0.34739	0.02370
**Comamonas**	0.02111	0.00120	0.00723
**Coprococcus**	0.24456	0.22533	0.10896
**Dialister**	0.45403	1.17986	0.92201
**Dorea**	0.16615	0.47827	0.07919
**Elusimicrobium**	0.00110	0.00000	0.00347
**Enterobacter**	0.00055	0.00400	0.00087
**Erysipelotrichaceae_incertae_sedis**	0.03318	0.12607	0.00087
**Escherichia_Shigella**	0.05264	0.16329	0.30926
**Faecalibacterium**	14.74270	12.25200	9.15993
**Flavonifractor**	0.00082	0.09645	0.00000
**Fusicatenibacter**	0.04277	0.25774	0.00896
**Fusobacterium**	0.00000	0.00240	0.00145
**Gemella**	0.00000	0.00080	0.00000
**Gemmiger**	0.17575	0.62095	0.14596
**Granulicatella**	0.00165	0.00320	0.00058
**Haemophilus**	0.74192	0.19611	0.02226
**Hallella**	0.00850	0.00400	0.00087
**Hespellia**	0.00329	0.00000	0.00087
**Howardella**	0.01179	0.00000	0.00549
**Intestinimonas**	0.03180	0.00760	0.01792
**Klebsiella**	0.51243	0.06564	0.00838
**Lachnospiracea_incertae_sedis**	0.33312	0.33018	0.07862
**Lactobacillus**	0.73698	2.35451	0.17111
**Leptotrichia**	0.00027	0.00040	0.00000
**Megamonas**	0.00027	1.35755	0.00000
**Megasphaera**	0.48255	0.40022	0.00202
**Methylobacterium**	0.00521	0.06043	0.00231
**Mitsuokella**	0.18754	0.47346	0.15174
**Neisseria**	0.00603	0.00240	0.00087
**Odoribacter**	0.03482	1.23549	0.01041
**Olsenella**	0.00384	0.07204	0.06041
**Oribacterium**	0.15820	0.00600	0.00173

Genus	W_India_T60_27	W_India_T60_28	W_India_T60_29
**Oscillibacter**	0.04250	0.34099	0.02081
**Parabacteroides**	0.07924	0.80845	0.04624
**Paraprevotella**	0.11269	0.48587	0.00029
**Parasutterella**	0.00466	0.41423	0.00520
**Peptostreptococcus**	0.00110	0.00080	0.00000
**Prevotella**	66.48400	11.94100	64.68200
**Pseudoflavonifractor**	0.00411	0.00040	0.00173
**Pseudomonas**	0.00000	0.00000	0.00000
**Ralstonia**	0.00000	0.00000	0.00000
**Rhizobium**	0.00027	0.00360	0.03497
**Roseburia**	5.54383	6.92345	1.65296
**Rothia**	0.00274	0.00961	0.00087
**Ruminococcus**	0.29748	1.63811	0.08873
**Ruminococcus2**	0.04825	0.07684	0.04653
**Saccharibacteria_genera_incertae_sedis**	0.00302	0.01241	0.02861
**Slackia**	0.00000	0.00040	0.00289
**Solobacterium**	0.00000	0.00000	0.00000
**Streptococcus**	0.20974	0.52029	0.03526
**Streptophyta**	0.00192	0.00280	0.00289
**Succinivibrio**	0.00658	7.86357	0.00838
**Sutterella**	1.05558	0.26695	0.43672
**Turicibacter**	0.03400	0.01401	0.17486
**Veillonella**	0.07512	0.38181	0.00260
**Weissella**	0.00055	0.05043	0.00029

Genus	W_India_T60_3	W_India_T60_30	W_India_T60_31
**Acinetobacter**	0.02580	0.00098	0.00169
**Actinomyces**	0.04401	0.00098	0.00338
**Akkermansia**	0.00000	0.00000	0.01126
**Alistipes**	0.03187	0.10256	0.53762
**Allisonella**	0.00000	0.00537	0.00169
**Alloprevotella**	0.18816	7.72458	0.01238
**Anaerostipes**	0.01062	0.00098	0.00113
**Bacteroides**	1.37782	0.23442	0.42953
**Barnesiella**	0.00000	0.00000	0.09063
**Bifidobacterium**	23.64460	0.46298	0.40645
**Bilophila**	0.00000	0.05812	0.00788
**Blautia**	0.73140	0.30377	0.07375
**Butyricicoccus**	0.31411	0.04347	0.01295
**Butyricimonas**	0.00759	0.01026	0.01070
**Campylobacter**	0.00000	0.00147	0.00000
**Catenibacterium**	0.01366	0.32379	0.00056
**Clostridium_IV**	0.00303	0.07326	0.00507
**Clostridium_sensu_stricto**	11.67810	0.01319	0.04785
**Clostridium_XI**	2.43547	0.00293	0.01070
**Clostridium_XlVa**	0.02276	0.09865	0.07431
**Clostridium_XlVb**	0.00000	0.02198	0.02927
**Collinsella**	0.00152	0.06642	0.06136
**Comamonas**	0.00303	0.03077	0.07318
**Coprococcus**	0.03338	0.90984	0.07938
**Dialister**	0.28376	0.20512	0.21617
**Dorea**	0.32776	0.20170	0.08275
**Elusimicrobium**	0.00000	0.00000	0.00113
**Enterobacter**	0.01517	0.00098	0.00000
**Erysipelotrichaceae_incertae_sedis**	0.00455	0.10354	0.19309
**Escherichia_Shigella**	4.18810	0.15091	0.40026
**Faecalibacterium**	2.80572	9.08372	9.99122
**Flavonifractor**	0.02124	0.00000	0.00056
**Fusicatenibacter**	0.40819	0.02833	0.02477
**Fusobacterium**	0.01517	0.00244	0.00000
**Gemella**	0.03490	0.00000	0.00056
**Gemmiger**	0.19727	0.24516	0.50722
**Granulicatella**	0.00759	0.00049	0.00281
**Haemophilus**	0.59787	0.05470	0.07712
**Hallella**	0.00000	0.00635	0.00000
**Hespellia**	0.00000	0.00293	0.00056
**Howardella**	0.00000	0.00000	0.00000
**Intestinimonas**	0.00000	0.05958	0.01351
**Klebsiella**	0.26707	0.01856	0.95532
**Lachnospiracea_incertae_sedis**	0.32776	0.30670	0.22405
**Lactobacillus**	19.39420	0.09523	0.19197
**Leptotrichia**	0.01062	0.00049	0.00000
**Megamonas**	0.00455	0.00000	0.00113
**Megasphaera**	0.01669	0.13137	0.16044
**Methylobacterium**	0.00759	0.00244	0.00000
**Mitsuokella**	0.01366	0.13528	0.19928
**Neisseria**	0.04401	0.01026	0.00225
**Odoribacter**	0.00000	0.09767	0.00732
**Olsenella**	0.03035	0.00977	0.03040
**Oribacterium**	0.00152	0.00586	0.00563

Genus	W_India_T60_3	W_India_T60_30	W_India_T60_31
**Oscillibacter**	0.00152	0.04444	0.03265
**Parabacteroides**	0.00759	0.22661	0.10809
**Paraprevotella**	0.00000	0.01123	0.27472
**Parasutterella**	0.32928	0.00977	0.00000
**Peptostreptococcus**	0.02428	0.00049	0.00225
**Prevotella**	5.83147	55.27490	67.77290
**Pseudoflavonifractor**	0.00000	0.00000	0.00056
**Pseudomonas**	0.00000	0.00000	0.00000
**Ralstonia**	0.00000	0.00000	0.00000
**Rhizobium**	0.00607	0.00000	0.00000
**Roseburia**	0.82396	2.80765	0.68342
**Rothia**	0.05311	0.00586	0.00619
**Ruminococcus**	0.01821	0.31451	0.04278
**Ruminococcus2**	0.24734	0.03028	0.04053
**Saccharibacteria_genera_incertae_sedis**	0.05463	0.00098	0.00225
**Slackia**	0.00000	0.00586	0.00056
**Solobacterium**	0.00000	0.00000	0.00000
**Streptococcus**	19.54900	0.36188	0.46781
**Streptophyta**	0.05311	0.02100	0.15312
**Succinivibrio**	0.55234	18.56590	14.40140
**Sutterella**	0.06373	0.72279	0.66484
**Turicibacter**	0.01669	0.00879	0.00169
**Veillonella**	2.29587	0.01074	0.01576
**Weissella**	0.30045	0.00098	0.00676

Genus	W_India_T60_32	W_India_T60_33	W_India_T60_34
**Acinetobacter**	0.00000	0.00000	0.00000
**Actinomyces**	0.00087	0.00288	0.00000
**Akkermansia**	0.00000	0.00000	0.00811
**Alistipes**	0.04312	0.08552	0.01622
**Allisonella**	0.00291	0.00000	0.00000
**Alloprevotella**	1.51390	8.64361	8.73918
**Anaerostipes**	0.00291	0.00000	0.00000
**Bacteroides**	0.56407	0.22166	0.14252
**Barnesiella**	0.00758	0.20052	0.02897
**Bifidobacterium**	0.09761	0.24312	0.55503
**Bilophila**	0.00146	0.01121	0.00232
**Blautia**	0.15908	0.06310	0.07416
**Butyricicoccus**	0.02855	0.06566	0.04171
**Butyricimonas**	0.00350	0.01409	0.00000
**Campylobacter**	0.14218	0.00032	0.00000
**Catenibacterium**	0.43325	0.23671	0.16570
**Clostridium_IV**	0.00903	0.09001	0.01043
**Clostridium_sensu_stricto**	0.05769	0.10026	0.30938
**Clostridium_XI**	0.04312	0.23703	0.22711
**Clostridium_XlVa**	0.09964	0.12845	0.12746
**Clostridium_XlVb**	0.00670	0.00416	0.00463
**Collinsella**	0.02972	0.03139	0.02665
**Comamonas**	0.00612	0.01153	0.00927
**Coprococcus**	0.15325	0.22966	0.04519
**Dialister**	0.19201	0.50065	0.17729
**Dorea**	0.10693	0.03427	0.03129
**Elusimicrobium**	0.09003	0.00096	0.00695
**Enterobacter**	0.00146	0.00096	0.00000
**Erysipelotrichaceae_incertae_sedis**	0.03992	0.01890	0.01854
**Escherichia_Shigella**	0.05244	0.39462	0.71378
**Faecalibacterium**	8.65713	6.62533	12.70090
**Flavonifractor**	0.00641	0.00032	0.00000
**Fusicatenibacter**	0.04021	0.02242	0.00463
**Fusobacterium**	0.00000	0.00032	0.00116
**Gemella**	0.00000	0.00160	0.00000
**Gemmiger**	0.14306	0.15471	0.18192
**Granulicatella**	0.00058	0.00128	0.00000
**Haemophilus**	0.10227	0.20532	0.02433
**Hallella**	0.00320	0.00032	0.00000
**Hespellia**	0.00087	0.00224	0.00000
**Howardella**	0.00029	0.02691	0.00348
**Intestinimonas**	0.03555	0.00961	0.01622
**Klebsiella**	0.00874	1.36132	0.40556
**Lachnospiracea_incertae_sedis**	0.38255	0.27867	0.24218
**Lactobacillus**	0.66284	0.13037	0.79605
**Leptotrichia**	0.00000	0.00032	0.00000
**Megamonas**	0.00029	0.00128	0.00232
**Megasphaera**	0.09032	0.24760	0.09154
**Methylobacterium**	0.00146	0.00160	0.00232
**Mitsuokella**	0.14131	0.53204	0.01275
**Neisseria**	0.00146	0.00480	0.00000
**Odoribacter**	0.01253	0.04036	0.00463
**Olsenella**	0.00524	0.01634	0.02202
**Oribacterium**	0.00932	0.00192	0.00000

Genus	W_India_T60_32	W_India_T60_33	W_India_T60_34
**Oscillibacter**	0.03263	0.02498	0.09038
**Parabacteroides**	0.11567	0.07175	0.03476
**Paraprevotella**	0.02535	0.01570	0.01738
**Parasutterella**	0.00903	0.00192	0.00000
**Peptostreptococcus**	0.00000	0.00320	0.00116
**Prevotella**	80.15530	76.33190	70.51600
**Pseudoflavonifractor**	0.00058	0.00224	0.00116
**Pseudomonas**	0.00000	0.00000	0.00000
**Ralstonia**	0.00000	0.00000	0.00000
**Rhizobium**	0.00000	0.00000	0.00000
**Roseburia**	1.51448	0.64030	2.36034
**Rothia**	0.00233	0.00224	0.00232
**Ruminococcus**	0.30330	0.05830	0.01390
**Ruminococcus2**	0.03321	0.03620	0.00463
**Saccharibacteria_genera_incertae_sedis**	0.00175	0.00256	0.00116
**Slackia**	0.00262	0.00032	0.00000
**Solobacterium**	0.00000	0.00000	0.00000
**Streptococcus**	0.09149	0.17489	0.01043
**Streptophyta**	0.03933	0.03748	0.00000
**Succinivibrio**	3.22446	0.02210	0.00811
**Sutterella**	0.11975	0.94140	0.59211
**Turicibacter**	0.05565	0.05573	0.28737
**Veillonella**	0.01952	0.04036	0.00579
**Weissella**	0.03176	0.00000	0.00463

Genus	W_India_T60_35	W_India_T60_36	W_India_T60_37
**Acinetobacter**	0.00000	0.00354	0.00000
**Actinomyces**	0.00253	0.01417	0.00191
**Akkermansia**	0.00000	0.00236	0.00095
**Alistipes**	0.00316	0.00591	0.00000
**Allisonella**	0.00000	0.00118	0.00095
**Alloprevotella**	0.02213	0.23150	10.73480
**Anaerostipes**	0.00000	0.00000	0.00000
**Bacteroides**	0.89791	0.15001	0.33376
**Barnesiella**	0.00316	0.00709	0.00095
**Bifidobacterium**	0.53748	24.93740	0.24126
**Bilophila**	0.00063	0.00236	0.00000
**Blautia**	0.04742	0.02835	0.13541
**Butyricicoccus**	0.12836	0.00945	0.03338
**Butyricimonas**	0.00000	0.00118	0.00000
**Campylobacter**	0.00190	0.01654	0.00191
**Catenibacterium**	0.78915	0.11575	0.15067
**Clostridium_IV**	0.00126	0.00354	0.01526
**Clostridium_sensu_stricto**	0.07398	0.00591	0.68374
**Clostridium_XI**	0.09548	0.01299	0.31088
**Clostridium_XlVa**	0.18085	0.03189	0.17546
**Clostridium_XlVb**	0.03288	0.00118	0.02861
**Collinsella**	0.02845	0.00354	0.02956
**Comamonas**	0.00000	0.00000	0.06771
**Coprococcus**	0.03604	0.02717	0.38240
**Dialister**	0.58048	0.04252	0.51876
**Dorea**	0.04742	0.03189	0.18119
**Elusimicrobium**	0.00253	0.00709	0.00095
**Enterobacter**	0.01707	0.00000	0.00668
**Erysipelotrichaceae_incertae_sedis**	0.00190	0.00472	0.00000
**Escherichia_Shigella**	0.77587	4.42337	0.08010
**Faecalibacterium**	11.95740	1.60635	9.30911
**Flavonifractor**	0.00822	0.00000	0.00000
**Fusicatenibacter**	0.01201	0.01181	0.00668
**Fusobacterium**	0.00063	0.04134	0.00000
**Gemella**	0.00190	0.02008	0.00286
**Gemmiger**	0.18211	0.03425	0.08869
**Granulicatella**	0.00190	0.02717	0.00000
**Haemophilus**	0.28961	0.49372	0.17451
**Hallella**	0.00000	0.00000	0.00095
**Hespellia**	0.00063	0.00000	0.00000
**Howardella**	0.00000	0.00000	0.00000
**Intestinimonas**	0.08157	0.00827	0.00954
**Klebsiella**	0.35916	27.52290	0.06771
**Lachnospiracea_incertae_sedis**	0.17769	0.02362	0.18977
**Lactobacillus**	0.09106	14.22560	0.27082
**Leptotrichia**	0.00000	0.07796	0.00000
**Megamonas**	0.48753	0.00945	0.12969
**Megasphaera**	0.13342	0.04606	0.00191
**Methylobacterium**	0.00000	0.00709	0.00477
**Mitsuokella**	0.00190	0.04961	0.20789
**Neisseria**	0.00696	0.08740	0.00000
**Odoribacter**	0.00000	0.00118	0.00000
**Olsenella**	0.00190	0.00118	0.00954
**Oribacterium**	0.00443	0.00000	0.00191

Genus	W_India_T60_35	W_India_T60_36	W_India_T60_37
Oscillibacter	0.05817	0.01063	0.04577
Parabacteroides	0.00379	0.03189	0.11539
Paraprevotella	0.00190	0.01063	0.00000
Parasutterella	0.00000	0.00118	0.00000
Peptostreptococcus	0.00190	0.00591	0.00000
Prevotella	76.83830	12.36180	71.79140
Pseudoflavonifractor	0.00000	0.00000	0.00000
Pseudomonas	0.00000	0.00000	0.00000
Ralstonia	0.00000	0.00000	0.00000
Rhizobium	0.00063	0.00000	0.00286
Roseburia	3.57204	0.28466	1.54770
Rothia	0.00885	0.12520	0.00572
Ruminococcus	0.01328	0.05906	0.14781
Ruminococcus2	0.02276	0.01299	0.01907
Saccharibacteria_genera_incertae_sedis	0.00253	0.04488	0.00286
Slackia	0.00000	0.00118	0.00000
Solobacterium	0.00000	0.00000	0.00000
Streptococcus	0.07651	6.15492	0.59982
Streptophyta	0.01012	0.01063	0.06008
Succinivibrio	0.09169	0.40041	0.00191
Sutterella	1.03133	5.10370	0.80103
Turicibacter	0.05312	0.09331	0.07247
Veillonella	0.05754	0.12874	0.04005
Weissella	0.00126	0.00354	0.00095

Genus	W_India_T60_38	W_India_T60_39	W_India_T60_4
Acinetobacter	0.00069	0.00000	0.00290
Actinomyces	0.00555	0.00000	0.00000
Akkermansia	0.00139	0.00150	0.81720
Alistipes	0.01733	0.26335	1.29825
Allisonella	0.00901	0.00150	0.00869
Alloprevotella	2.59656	6.75836	6.15509
Anaerostipes	0.01248	0.00602	0.00000
Bacteroides	2.05104	0.53122	18.81010
Barnesiella	0.00000	0.04063	0.00580
Bifidobacterium	1.13885	0.29646	1.87493
Bilophila	0.00069	0.02408	0.01739
Blautia	0.40688	0.37923	0.26371
Butyricicoccus	0.12685	0.15049	0.02898
Butyricimonas	0.00000	0.00000	0.03767
Campylobacter	0.00555	0.00752	0.00000
Catenibacterium	1.40849	0.36117	0.15938
Clostridium_IV	0.33202	0.07223	0.01739
Clostridium_sensu_stricto	0.21834	0.09782	0.13330
Clostridium_XI	0.05961	0.00451	0.61725
Clostridium_XlVa	0.07209	0.14447	0.28979
Clostridium_XlVb	0.02149	0.08427	0.00290
Collinsella	0.14903	0.13092	0.21734
Comamonas	0.20032	0.00000	0.01449
Coprococcus	0.44431	0.79457	0.09563
Dialister	3.01245	0.93452	0.46366
Dorea	0.48174	0.58088	0.33905
Elusimicrobium	1.07162	0.00000	0.00000
Enterobacter	0.38401	0.00000	0.00000
Erysipelotrichaceae_incertae_sedis	0.44362	1.20088	0.03477
Escherichia_Shigella	0.72573	0.18510	0.05796
Faecalibacterium	8.36707	9.51378	14.90380
Flavonifractor	0.00069	0.00451	0.03477
Fusicatenibacter	0.14626	0.03612	0.18836
Fusobacterium	0.00208	0.00150	0.00000
Gemella	0.00069	0.00752	0.00000
Gemmiger	0.65087	0.58238	0.42599
Granulicatella	0.00069	0.00301	0.00290
Haemophilus	0.07139	0.00752	0.30428
Hallella	0.02357	0.02558	0.00000
Hespellia	0.00693	0.00000	0.00000
Howardella	0.01317	0.03160	0.00000
Intestinimonas	0.05129	0.15952	0.01159
Klebsiella	0.88031	0.00301	0.02029
Lachnospiracea_incertae_sedis	1.14994	0.35515	0.28399
Lactobacillus	0.13239	0.05568	0.96789
Leptotrichia	0.00069	0.00000	0.00000
Megamonas	0.00000	0.00000	0.68970
Megasphaera	0.89833	0.16253	0.35064
Methylobacterium	0.00277	0.00150	0.00869
Mitsuokella	0.87130	0.42287	0.41730
Neisseria	0.00347	0.00301	0.02898
Odoribacter	0.03327	0.07976	0.54770
Olsenella	0.78673	0.01655	0.01739
Oribacterium	0.38678	0.00752	0.00000

Genus	W_India_T60_38	W_India_T60_39	W_India_T60_4
**Oscillibacter**	0.05337	0.22573	0.02029
**Parabacteroides**	0.07902	1.30924	0.43468
**Paraprevotella**	0.00000	0.00000	0.31877
**Parasutterella**	0.00277	0.00000	0.32167
**Peptostreptococcus**	0.00208	0.00451	0.00000
**Prevotella**	61.69490	62.07130	35.37730
**Pseudoflavonifractor**	0.00901	0.01204	0.00000
**Pseudomonas**	0.00000	0.00000	0.00000
**Ralstonia**	0.00000	0.00000	0.00000
**Rhizobium**	0.00069	0.00000	0.00290
**Roseburia**	5.68387	4.64101	4.78440
**Rothia**	0.00416	0.01806	0.00290
**Ruminococcus**	1.02725	0.57937	0.57378
**Ruminococcus2**	0.23359	0.05418	0.05506
**Saccharibacteria_genera_incertae_sedis**	0.00347	0.00000	0.00580
**Slackia**	0.00624	0.00150	0.00290
**Solobacterium**	0.00000	0.00000	0.00000
**Streptococcus**	0.68622	0.09330	0.33615
**Streptophyta**	0.00347	0.00000	0.00000
**Succinivibrio**	0.04506	5.11505	5.63348
**Sutterella**	0.03882	0.14748	0.90993
**Turicibacter**	0.01040	0.00301	0.02608
**Veillonella**	0.02218	0.03311	0.13910
**Weissella**	0.00832	0.00301	0.02608

Genus	W_India_T60_40	W_India_T60_41	W_India_T60_42
**Acinetobacter**	0.00127	0.00129	0.00000
**Actinomyces**	0.00127	0.00032	0.00074
**Akkermansia**	0.00382	0.00000	0.00037
**Alistipes**	0.01910	0.00000	0.07960
**Allisonella**	0.00127	0.02968	0.00296
**Alloprevotella**	6.75383	0.00871	2.11513
**Anaerostipes**	0.00255	0.00032	0.00222
**Bacteroides**	0.15277	5.64907	0.95335
**Barnesiella**	0.00000	0.00000	0.01333
**Bifidobacterium**	0.23171	0.20421	0.62717
**Bilophila**	0.00000	0.00000	0.00333
**Blautia**	0.08021	0.01613	0.21066
**Butyricicoccus**	0.09803	0.00452	0.05220
**Butyricimonas**	0.00000	0.00000	0.00222
**Campylobacter**	0.12349	0.00032	0.00037
**Catenibacterium**	0.70021	0.26324	0.87375
**Clostridium_IV**	0.00764	0.00000	0.02147
**Clostridium_sensu_stricto**	0.03055	0.00000	0.00518
**Clostridium_XI**	0.51943	0.00000	0.00777
**Clostridium_XlVa**	0.11585	0.58455	0.11218
**Clostridium_XlVb**	0.00764	0.01097	0.01222
**Collinsella**	0.01655	0.05678	0.04036
**Comamonas**	0.00000	0.00000	0.01000
**Coprococcus**	0.31828	0.03968	0.22954
**Dialister**	0.48505	0.82392	0.36246
**Dorea**	0.10821	0.17808	0.13514
**Elusimicrobium**	0.13877	0.00032	0.00111
**Enterobacter**	0.00000	0.00194	0.00148
**Erysipelotrichaceae_incertae_sedis**	0.01146	0.00032	0.10441
**Escherichia_Shigella**	1.21072	0.37938	0.10663
**Faecalibacterium**	1.72888	0.24324	10.18660
**Flavonifractor**	0.00000	0.00581	0.01518
**Fusicatenibacter**	0.01018	0.00807	0.07590
**Fusobacterium**	0.00127	0.07226	0.00037
**Gemella**	0.00255	0.00129	0.00037
**Gemmiger**	0.10185	0.23066	0.20437
**Granulicatella**	0.00127	0.00323	0.00111
**Haemophilus**	0.72185	0.09614	0.14032
**Hallella**	0.00637	0.00000	0.00703
**Hespellia**	0.00000	0.00000	0.00037
**Howardella**	0.00127	0.00000	0.00000
**Intestinimonas**	0.00891	0.00000	0.03628
**Klebsiella**	0.27117	0.17130	0.16179
**Lachnospiracea_incertae_sedis**	0.14259	0.27937	0.20363
**Lactobacillus**	0.34119	0.00968	1.39059
**Leptotrichia**	0.00127	0.00000	0.00000
**Megamonas**	0.00000	1.25266	0.00148
**Megasphaera**	0.06238	5.12904	0.23214
**Methylobacterium**	0.00127	0.00290	0.00370
**Mitsuokella**	0.28645	0.35454	0.22917
**Neisseria**	0.01910	0.00355	0.00333
**Odoribacter**	0.00127	0.00000	0.02073
**Olsenella**	0.00255	0.00129	0.01074
**Oribacterium**	0.02292	0.00548	0.00222

Genus	W_India_T60_40	W_India_T60_41	W_India_T60_42
**Oscillibacter**	0.03947	0.00000	0.03554
**Parabacteroides**	0.01273	0.05452	0.20437
**Paraprevotella**	0.00382	0.00000	0.05739
**Parasutterella**	0.00127	0.00000	0.08997
**Peptostreptococcus**	0.00000	0.00000	0.00037
**Prevotella**	56.65710	66.89170	76.42960
**Pseudoflavonifractor**	0.00382	0.00000	0.00370
**Pseudomonas**	0.00000	0.00000	0.00000
**Ralstonia**	0.00000	0.00000	0.00000
**Rhizobium**	0.00000	0.00032	0.00000
**Roseburia**	1.84473	0.52455	1.92706
**Rothia**	0.00127	0.00323	0.00740
**Ruminococcus**	0.05092	0.00065	0.55794
**Ruminococcus2**	0.02164	0.11549	0.04813
**Saccharibacteria_genera_incertae_sedis**	0.00382	0.00194	0.00037
**Slackia**	0.00000	0.00000	0.00481
**Solobacterium**	0.00000	0.00000	0.00000
**Streptococcus**	0.28772	5.63843	0.39282
**Streptophyta**	0.00127	0.01968	0.06997
**Succinivibrio**	25.26740	0.00194	0.82525
**Sutterella**	0.43158	0.24937	0.20733
**Turicibacter**	0.17187	0.00032	0.01111
**Veillonella**	0.15787	0.09872	0.04369
**Weissella**	0.00000	0.70134	0.00000

Genus	W_India_T60_43	W_India_T60_44	W_India_T60_45
**Acinetobacter**	0.00000	0.00150	0.00502
**Actinomyces**	0.00125	0.00150	0.00251
**Akkermansia**	0.00000	0.11465	0.00000
**Alistipes**	0.79125	1.31364	0.00000
**Allisonella**	0.00000	0.00075	0.02929
**Alloprevotella**	1.64509	0.06295	0.00670
**Anaerostipes**	0.00000	0.00150	0.00167
**Bacteroides**	2.30613	42.67040	29.45780
**Barnesiella**	0.00626	0.00075	0.00000
**Bifidobacterium**	0.07011	1.12930	1.73912
**Bilophila**	0.05258	0.00000	0.00000
**Blautia**	0.17027	0.38218	0.24438
**Butyricicoccus**	0.07011	0.03372	0.12386
**Butyricimonas**	0.15525	0.00000	0.00000
**Campylobacter**	0.00000	0.00450	0.00084
**Catenibacterium**	0.17903	0.72539	0.00167
**Clostridium_IV**	0.43944	0.00749	0.00000
**Clostridium_sensu_stricto**	0.04132	0.00824	0.02092
**Clostridium_XI**	0.14273	0.01574	0.07365
**Clostridium_XlVa**	0.20407	0.14313	0.23434
**Clostridium_XlVb**	0.00000	0.00749	0.00000
**Collinsella**	0.02379	0.03522	0.16404
**Comamonas**	0.01002	0.00075	0.00084
**Coprococcus**	0.17653	0.28851	0.08453
**Dialister**	0.35932	0.22031	0.88128
**Dorea**	0.26667	0.35220	0.94070
**Elusimicrobium**	0.00000	0.00300	0.00000
**Enterobacter**	0.00000	0.00450	0.00251
**Erysipelotrichaceae_incertae_sedis**	0.09265	0.00674	0.10294
**Escherichia_Shigella**	0.35556	0.39866	1.31061
**Faecalibacterium**	12.99300	19.02340	2.02702
**Flavonifractor**	0.03756	0.00150	0.04603
**Fusicatenibacter**	0.04257	0.21207	0.17492
**Fusobacterium**	0.00000	0.00150	0.00251
**Gemella**	0.00000	0.00150	0.00335
**Gemmiger**	0.62348	0.27127	0.08537
**Granulicatella**	0.00000	0.00150	0.00084
**Haemophilus**	0.01753	0.73438	0.01004
**Hallella**	0.00501	0.01499	0.00000
**Hespellia**	0.00000	0.00075	0.00000
**Howardella**	0.00626	0.00000	0.00000
**Intestinimonas**	0.11518	0.01724	0.00000
**Klebsiella**	0.01377	0.05395	0.11298
**Lachnospiracea_incertae_sedis**	0.54461	0.17910	0.53730
**Lactobacillus**	0.00376	0.41365	1.83955
**Leptotrichia**	0.00250	0.00150	0.00084
**Megamonas**	0.00000	0.41815	0.00000
**Megasphaera**	0.37810	0.15962	0.61514
**Methylobacterium**	0.00125	0.00150	0.00753
**Mitsuokella**	0.09139	0.02473	0.00000
**Neisseria**	0.00000	0.01199	0.01004
**Odoribacter**	0.05759	0.28551	0.00084
**Olsenella**	0.04382	0.01424	0.00167
**Oribacterium**	0.00751	0.00300	0.00251

Genus	W_India_T60_43	W_India_T60_44	W_India_T60_45
**Oscillibacter**	0.14773	0.20758	0.00418
**Parabacteroides**	0.68733	0.28326	0.87040
**Paraprevotella**	0.03631	0.50283	0.00000
**Parasutterella**	0.00125	0.28251	0.00000
**Peptostreptococcus**	0.00000	0.00225	0.00000
**Prevotella**	58.20790	19.10210	46.47240
**Pseudoflavonifractor**	0.02504	0.00075	0.00000
**Pseudomonas**	0.00000	0.00000	0.00000
**Ralstonia**	0.00000	0.00000	0.00000
**Rhizobium**	0.00000	0.00000	0.00000
**Roseburia**	2.97343	6.68510	4.37122
**Rothia**	0.00000	0.01049	0.00418
**Ruminococcus**	0.71237	0.74787	0.00000
**Ruminococcus2**	0.14899	0.04047	0.03766
**Saccharibacteria_genera_incertae_sedis**	0.00250	0.00974	0.00084
**Slackia**	0.00501	0.00225	0.00000
**Solobacterium**	0.00000	0.00000	0.00000
**Streptococcus**	0.34554	0.99666	4.41976
**Streptophyta**	0.00000	0.00824	0.01172
**Succinivibrio**	13.53130	0.11016	0.00335
**Sutterella**	0.53334	0.73213	2.82209
**Turicibacter**	0.02128	0.02548	0.00167
**Veillonella**	0.01002	0.41440	0.00753
**Weissella**	0.00000	0.00599	0.00000

Genus	W_India_T60_46	W_India_T60_47	W_India_T60_48
**Acinetobacter**	0.00547	0.00000	0.00208
**Actinomyces**	0.00000	0.00582	0.00625
**Akkermansia**	0.00328	0.00000	0.00000
**Alistipes**	0.03171	0.25601	0.00104
**Allisonella**	0.01093	0.00000	0.00208
**Alloprevotella**	0.08310	8.36097	12.88920
**Anaerostipes**	0.00219	0.00000	0.00000
**Bacteroides**	1.38752	0.24437	0.13444
**Barnesiella**	0.00109	0.01746	0.00104
**Bifidobacterium**	1.06825	3.14191	0.57422
**Bilophila**	0.00109	0.00000	0.00000
**Blautia**	0.56638	0.45383	0.31264
**Butyricicoccus**	0.03062	0.07564	0.06044
**Butyricimonas**	0.00109	0.01746	0.00000
**Campylobacter**	0.00547	0.00000	0.00104
**Catenibacterium**	1.11417	0.06982	0.75451
**Clostridium_IV**	0.00656	0.00582	0.00313
**Clostridium_sensu_stricto**	0.00984	0.20364	0.00417
**Clostridium_XI**	0.00875	0.26764	0.01042
**Clostridium_XlVa**	0.45157	1.07058	0.15320
**Clostridium_XlVb**	0.00875	0.08728	0.01563
**Collinsella**	0.24711	0.26183	0.12818
**Comamonas**	0.00000	0.08146	0.00938
**Coprococcus**	0.14652	0.06982	0.17091
**Dialister**	0.36082	0.06400	0.51482
**Dorea**	0.34661	0.04655	0.51899
**Elusimicrobium**	0.00219	0.08728	0.00000
**Enterobacter**	0.00000	0.00000	0.00625
**Erysipelotrichaceae_incertae_sedis**	0.00328	0.17455	0.10943
**Escherichia_Shigella**	0.13886	0.15128	0.09171
**Faecalibacterium**	7.85497	10.84540	8.85718
**Flavonifractor**	0.00000	0.00000	0.00730
**Fusicatenibacter**	0.01968	0.00000	0.03752
**Fusobacterium**	0.00984	0.01164	0.00625
**Gemella**	0.00219	0.00000	0.00208
**Gemmiger**	0.16948	1.35568	0.17195
**Granulicatella**	0.00000	0.00000	0.00625
**Haemophilus**	0.96438	0.86112	0.03439
**Hallella**	0.00547	0.00000	0.00417
**Hespellia**	0.00000	0.00000	0.00313
**Howardella**	0.00000	0.00000	0.00000
**Intestinimonas**	0.05358	0.01746	0.04481
**Klebsiella**	0.12902	0.01746	0.00730
**Lachnospiracea_incertae_sedis**	0.40893	0.34328	0.18446
**Lactobacillus**	0.56857	0.00582	0.11151
**Leptotrichia**	0.02843	0.00000	0.00938
**Megamonas**	1.01905	0.02909	0.00000
**Megasphaera**	0.24164	0.00582	0.36162
**Methylobacterium**	0.02296	0.00000	0.00834
**Mitsuokella**	0.00437	0.00582	0.44500
**Neisseria**	0.14214	0.00582	0.01042
**Odoribacter**	0.00547	0.01746	0.00104
**Olsenella**	0.00109	0.00000	0.03126
**Oribacterium**	0.00109	0.00000	0.00625

Genus	W_India_T60_46	W_India_T60_47	W_India_T60_48
**Oscillibacter**	0.01968	0.11055	0.10943
**Parabacteroides**	0.06342	0.54693	0.00000
**Paraprevotella**	0.01749	0.04655	0.00104
**Parasutterella**	0.01203	0.00582	0.00104
**Peptostreptococcus**	0.00219	0.00000	0.00104
**Prevotella**	69.34770	67.08560	60.02330
**Pseudoflavonifractor**	0.00000	0.01746	0.00000
**Pseudomonas**	0.00000	0.00000	0.00000
**Ralstonia**	0.00000	0.00000	0.00000
**Rhizobium**	0.00219	0.00000	0.00000
**Roseburia**	5.79720	1.15203	2.95656
**Rothia**	0.04155	0.00000	0.01251
**Ruminococcus**	0.02515	0.26183	0.08858
**Ruminococcus2**	0.08966	0.02327	0.06566
**Saccharibacteria_genera_incertae_sedis**	0.01968	0.00582	0.00313
**Slackia**	0.01531	0.00582	0.00313
**Solobacterium**	0.00000	0.00000	0.00000
**Streptococcus**	2.15509	0.40729	0.08546
**Streptophyta**	0.03499	0.04655	0.00417
**Succinivibrio**	0.13668	0.04073	8.96661
**Sutterella**	4.25551	0.41310	0.72846
**Turicibacter**	0.00547	0.40729	0.00834
**Veillonella**	0.11699	0.01746	0.02710
**Weissella**	0.04920	0.00000	0.00000

Genus	W_India_T60_49	W_India_T60_5	W_India_T60_50
**Acinetobacter**	0.00111	0.00055	0.00231
**Actinomyces**	0.00334	0.00109	0.00404
**Akkermansia**	0.00000	0.00000	0.00115
**Alistipes**	0.02230	0.01912	0.17023
**Allisonella**	0.03679	0.00710	0.01039
**Alloprevotella**	2.44843	7.82358	0.58918
**Anaerostipes**	0.01784	0.00328	0.01270
**Bacteroides**	0.74367	0.10270	7.21091
**Barnesiella**	0.00111	0.00000	0.00404
**Bifidobacterium**	0.44487	0.77460	1.88698
**Bilophila**	0.00446	0.00328	0.04501
**Blautia**	0.35456	0.12509	0.56205
**Butyricicoccus**	0.09589	0.12346	0.10156
**Butyricimonas**	0.00334	0.00000	0.07271
**Campylobacter**	0.00111	0.00601	0.00115
**Catenibacterium**	0.03679	0.49765	0.59841
**Clostridium_IV**	0.00780	0.00601	0.01385
**Clostridium_sensu_stricto**	2.37039	0.07211	0.09175
**Clostridium_XI**	0.48277	0.07593	0.31969
**Clostridium_XlVa**	0.30327	0.57303	0.33296
**Clostridium_XlVb**	0.05909	0.01093	0.04386
**Collinsella**	0.02230	0.03496	1.00870
**Comamonas**	0.00000	0.03878	0.00231
**Coprococcus**	0.23080	0.10761	0.64284
**Dialister**	0.01784	1.21871	1.40225
**Dorea**	0.42368	0.12509	0.33758
**Elusimicrobium**	0.00000	0.00000	0.00115
**Enterobacter**	0.00111	0.00492	0.00346
**Erysipelotrichaceae_incertae_sedis**	0.67009	0.21031	0.37220
**Escherichia_Shigella**	13.38050	0.02513	0.17600
**Faecalibacterium**	5.83566	11.98390	13.80440
**Flavonifractor**	0.00669	0.00546	0.00115
**Fusicatenibacter**	0.05017	0.02895	0.23717
**Fusobacterium**	0.01449	0.00055	0.00346
**Gemella**	0.00223	0.00000	0.00346
**Gemmiger**	0.31219	0.12509	0.76460
**Granulicatella**	0.00446	0.00000	0.00404
**Haemophilus**	1.76608	1.73329	0.43683
**Hallella**	0.00446	0.00055	0.00808
**Hespellia**	0.00557	0.00055	0.00000
**Howardella**	0.00000	0.00000	0.00404
**Intestinimonas**	0.01449	0.03114	0.01731
**Klebsiella**	0.10481	0.65497	0.04213
**Lachnospiracea_incertae_sedis**	1.10603	0.17590	0.59033
**Lactobacillus**	0.02341	0.36490	1.12065
**Leptotrichia**	0.00111	0.00000	0.01270
**Megamonas**	0.28208	0.57795	3.43580
**Megasphaera**	0.02007	1.66610	0.60937
**Methylobacterium**	0.00000	0.00164	0.02828
**Mitsuokella**	0.06913	0.25511	0.56494
**Neisseria**	0.07136	0.06391	0.03982
**Odoribacter**	0.11707	0.02731	0.15811
**Olsenella**	0.00892	0.00164	0.12638
**Oribacterium**	0.01115	0.00273	0.01039

Genus	W_India_T60_49	W_India_T60_5	W_India_T60_50
**Oscillibacter**	0.02118	0.01803	0.05251
**Parabacteroides**	0.06913	0.06227	0.14657
**Paraprevotella**	0.02564	0.01147	0.24121
**Parasutterella**	0.00892	0.00000	0.08367
**Peptostreptococcus**	0.00000	0.00164	0.00000
**Prevotella**	56.41210	65.02990	39.62080
**Pseudoflavonifractor**	0.00111	0.00055	0.00000
**Pseudomonas**	0.00000	0.00000	0.00000
**Ralstonia**	0.00000	0.00000	0.00000
**Rhizobium**	0.00000	0.00000	0.00115
**Roseburia**	0.57532	2.26208	5.25930
**Rothia**	0.01226	0.01967	0.01904
**Ruminococcus**	0.01003	0.00492	0.46280
**Ruminococcus2**	0.33226	0.05790	0.22852
**Saccharibacteria_genera_incertae_sedis**	0.00780	0.00382	0.01673
**Slackia**	0.00000	0.00109	0.00923
**Solobacterium**	0.00000	0.00000	0.00000
**Streptococcus**	1.01907	1.84309	1.05890
**Streptophyta**	0.00223	0.00164	0.00692
**Succinivibrio**	2.16970	0.01256	12.88630
**Sutterella**	0.06244	0.57522	1.51766
**Turicibacter**	5.40417	0.29225	0.03289
**Veillonella**	0.00780	0.07156	0.11195
**Weissella**	0.00223	0.00109	0.00462

Genus	W_India_T60_51	W_India_T60_52	W_India_T60_53
**Acinetobacter**	0.00000	0.00187	0.00386
**Actinomyces**	0.00139	0.00094	0.00258
**Akkermansia**	0.00139	0.00000	0.00386
**Alistipes**	0.04659	0.24057	0.18616
**Allisonella**	0.00070	0.00000	0.00322
**Alloprevotella**	3.69389	0.00562	0.12819
**Anaerostipes**	0.00139	0.00000	0.00322
**Bacteroides**	0.09318	1.66902	14.47750
**Barnesiella**	0.00000	0.00000	0.52048
**Bifidobacterium**	0.19540	1.58290	1.93634
**Bilophila**	0.01391	0.01030	0.00064
**Blautia**	0.13143	0.20126	0.48763
**Butyricicoccus**	0.11891	0.03370	0.04316
**Butyricimonas**	0.04172	0.07114	0.00000
**Campylobacter**	0.00209	0.00094	0.00451
**Catenibacterium**	0.64393	0.86681	1.47448
**Clostridium_IV**	0.10153	0.02808	0.00902
**Clostridium_sensu_stricto**	0.11891	0.03651	0.05411
**Clostridium_XI**	0.11961	0.15539	0.45220
**Clostridium_XlVa**	0.07371	0.29112	0.45478
**Clostridium_XlVb**	0.00834	0.01217	0.02448
**Collinsella**	0.02921	0.12637	0.22739
**Comamonas**	0.00070	0.00000	0.00709
**Coprococcus**	0.17732	0.88459	0.48441
**Dialister**	0.99023	1.74765	0.29438
**Dorea**	0.27885	0.25087	0.34849
**Elusimicrobium**	0.00070	0.00187	0.00386
**Enterobacter**	0.00070	0.00094	0.00000
**Erysipelotrichaceae_incertae_sedis**	0.52988	0.29018	0.00644
**Escherichia_Shigella**	0.54657	0.06553	0.47153
**Faecalibacterium**	8.63322	15.83930	14.47810
**Flavonifractor**	0.00278	0.00281	0.00902
**Fusicatenibacter**	0.06954	0.03932	0.05733
**Fusobacterium**	0.00000	0.00000	0.00515
**Gemella**	0.00000	0.00000	0.01159
**Gemmiger**	0.28441	0.33980	1.85582
**Granulicatella**	0.00000	0.00000	0.00515
**Haemophilus**	0.14325	0.01311	0.67894
**Hallella**	0.00695	0.00000	0.00644
**Hespellia**	0.00695	0.00000	0.00000
**Howardella**	0.01460	0.00000	0.00064
**Intestinimonas**	0.01182	0.01779	0.03285
**Klebsiella**	0.38177	0.02902	0.22868
**Lachnospiracea_incertae_sedis**	0.30319	0.25929	0.29245
**Lactobacillus**	0.05215	0.91548	1.03323
**Leptotrichia**	0.00000	0.00000	0.00064
**Megamonas**	0.00000	0.28925	0.42901
**Megasphaera**	0.44574	0.24619	0.63192
**Methylobacterium**	0.00070	0.00281	0.00322
**Mitsuokella**	0.20097	0.12637	0.22095
**Neisseria**	0.00000	0.00000	0.01224
**Odoribacter**	0.02295	0.02340	0.08696
**Olsenella**	0.00139	0.01217	0.20355
**Oribacterium**	0.35256	0.02527	0.00000

Genus	W_India_T60_51	W_India_T60_52	W_India_T60_53
**Oscillibacter**	0.05285	0.02247	0.08052
**Parabacteroides**	0.03755	0.34728	1.57497
**Paraprevotella**	0.00000	0.31359	0.01932
**Parasutterella**	0.00000	0.01030	0.01353
**Peptostreptococcus**	0.00000	0.00000	0.01353
**Prevotella**	63.47070	63.53420	40.00040
**Pseudoflavonifractor**	0.00000	0.00000	0.00129
**Pseudomonas**	0.00000	0.00000	0.00000
**Ralstonia**	0.00000	0.00000	0.00000
**Rhizobium**	0.00070	0.00000	0.00064
**Roseburia**	5.64445	6.61805	10.10430
**Rothia**	0.00348	0.00187	0.02770
**Ruminococcus**	0.39359	0.39970	0.39036
**Ruminococcus2**	0.01808	0.07395	0.09276
**Saccharibacteria_genera_incertae_sedis**	0.00209	0.00094	0.01031
**Slackia**	0.00278	0.00468	0.00193
**Solobacterium**	0.00000	0.00000	0.00000
**Streptococcus**	0.03199	0.21062	1.13694
**Streptophyta**	0.00000	0.00094	0.01417
**Succinivibrio**	11.12060	0.03276	0.14107
**Sutterella**	0.23156	1.43032	3.58861
**Turicibacter**	0.07023	0.16194	0.10822
**Veillonella**	0.00139	0.00000	0.11208
**Weissella**	0.00000	0.00187	0.02126

Genus	W_India_T60_54	W_India_T60_55	W_India_T60_56
Acinetobacter	0.00000	0.00333	0.00000
Actinomyces	0.00207	0.03807	0.00278
Akkermansia	0.00000	0.00000	0.00000
Alistipes	0.00000	0.01047	0.26649
Allisonella	0.00103	0.00143	0.01183
Alloprevotella	12.36140	3.35606	7.26129
Anaerostipes	0.00723	0.00286	0.00070
Bacteroides	0.47186	5.63103	6.44234
Barnesiella	0.01652	0.00000	0.63595
Bifidobacterium	0.02272	0.47964	0.33537
Bilophila	0.03614	0.03188	0.07167
Blautia	0.24677	0.15750	0.33050
Butyricicoccus	0.05989	0.16178	0.07445
Butyricimonas	0.00000	0.00000	0.13498
Campylobacter	0.00826	0.01142	0.00000
Catenibacterium	0.20651	0.63714	0.54968
Clostridium_IV	0.43676	0.00048	0.00278
Clostridium_sensu_stricto	0.28704	1.27904	0.11063
Clostridium_XI	0.12081	0.60574	0.01183
Clostridium_XlVa	0.12287	0.33356	0.17534
Clostridium_XlVb	0.11668	0.06138	0.01879
Collinsella	0.02065	0.04045	0.08489
Comamonas	0.24264	0.05662	0.00000
Coprococcus	0.62571	0.13514	0.40356
Dialister	0.18585	0.66522	0.65196
Dorea	0.26536	0.12181	0.22683
Elusimicrobium	0.00000	0.00000	0.00000
Enterobacter	0.00310	0.00000	0.00000
Erysipelotrichaceae_incertae_sedis	0.19412	0.14846	0.01809
Escherichia_Shigella	0.35209	1.49745	0.48775
Faecalibacterium	4.61332	3.17858	6.54323
Flavonifractor	0.12907	0.02332	0.00835
Fusicatenibacter	0.05782	0.02427	0.06471
Fusobacterium	0.00413	3.49501	0.00765
Gemella	0.00207	0.01190	0.00070
Gemmiger	0.07744	0.35783	0.23866
Granulicatella	0.00310	0.00286	0.00070
Haemophilus	0.15694	0.18367	0.00905
Hallella	0.00413	0.00999	0.00000
Hespellia	0.00310	0.01999	0.00000
Howardella	0.00620	0.01285	0.00000
Intestinimonas	0.03924	0.03949	0.07584
Klebsiella	0.03614	0.00809	0.00000
Lachnospiracea_incertae_sedis	0.77646	0.12372	0.22961
Lactobacillus	0.04543	0.00333	0.17395
Leptotrichia	0.00310	0.00048	0.00070
Megamonas	0.00310	0.17511	0.04244
Megasphaera	0.00103	0.00476	0.18786
Methylobacterium	0.00000	0.00286	0.00070
Mitsuokella	0.07847	0.00143	0.07445
Neisseria	0.02478	0.00238	0.00417
Odoribacter	0.07641	0.03188	0.03618
Olsenella	0.00000	0.00000	0.02227
Oribacterium	0.01136	0.00619	0.00278

Genus	W_India_T60_54	W_India_T60_55	W_India_T60_56
**Oscillibacter**	0.06195	0.01903	0.04105
**Parabacteroides**	0.08880	0.66474	0.66518
**Paraprevotella**	0.00413	0.00000	0.40286
**Parasutterella**	0.01446	0.00000	0.00209
**Peptostreptococcus**	0.00000	0.04140	0.00487
**Prevotella**	57.07070	67.90020	46.03090
**Pseudoflavonifractor**	0.00413	0.00095	0.00000
**Pseudomonas**	0.00000	0.00000	0.00000
**Ralstonia**	0.00000	0.00000	0.00000
**Rhizobium**	0.00000	0.00048	0.00348
**Roseburia**	4.04543	0.65237	1.73461
**Rothia**	0.01136	0.03807	0.00417
**Ruminococcus**	1.62726	0.00381	0.46966
**Ruminococcus2**	0.21890	0.18415	0.02853
**Saccharibacteria_genera_incertae_sedis**	0.00413	0.02427	0.00278
**Slackia**	0.00000	0.00238	0.00487
**Solobacterium**	0.00000	0.00000	0.00000
**Streptococcus**	0.08983	0.77656	0.11411
**Streptophyta**	0.04440	0.05091	0.00209
**Succinivibrio**	13.84410	0.00666	23.79120
**Sutterella**	0.16624	5.59724	0.62343
**Turicibacter**	0.04440	0.18843	0.00139
**Veillonella**	0.02168	0.43777	0.01183
**Weissella**	0.00207	0.00000	0.00000

Genus	W_India_T60_57	W_India_T60_58	W_India_T60_59
Acinetobacter	0.00071	0.00000	0.00000
Actinomyces	0.02350	0.00333	0.00330
Akkermansia	0.00000	0.00111	0.00494
Alistipes	0.00142	0.00000	0.13951
Allisonella	0.02279	0.01332	0.00055
Alloprevotella	12.87860	9.90155	4.82447
Anaerostipes	0.00142	0.00000	0.00714
Bacteroides	0.11182	0.01998	0.37732
Barnesiella	0.00000	0.00000	0.00000
Bifidobacterium	0.46862	0.61933	3.66558
Bilophila	0.00000	0.00000	0.05822
Blautia	0.15740	0.04329	0.15598
Butyricicoccus	0.00214	0.05217	0.07689
Butyricimonas	0.00142	0.00000	0.00000
Campylobacter	0.00000	0.00444	0.00220
Catenibacterium	0.43729	0.49058	0.47948
Clostridium_IV	0.00071	0.00222	0.07799
Clostridium_sensu_stricto	0.01140	0.28414	0.02801
Clostridium_XI	1.42510	0.63487	0.00604
Clostridium_XlVa	2.18501	0.03996	0.08898
Clostridium_XlVb	0.00071	0.00777	0.03515
Collinsella	0.00142	0.19979	0.05657
Comamonas	0.00000	0.00777	0.00000
Coprococcus	0.06766	0.06327	0.41247
Dialister	0.00712	0.64375	0.27956
Dorea	0.11182	0.03663	0.29878
Elusimicrobium	0.00142	0.02220	0.00000
Enterobacter	0.00285	0.00333	0.00000
Erysipelotrichaceae_incertae_sedis	0.00356	0.04440	0.44103
Escherichia_Shigella	0.09401	0.09434	0.00769
Faecalibacterium	0.04131	4.57063	11.24340
Flavonifractor	0.03490	0.00000	0.00000
Fusicatenibacter	0.00142	0.00444	0.06261
Fusobacterium	0.00214	0.00999	0.00055
Gemella	0.00071	0.01554	0.00000
Gemmiger	0.00214	0.38736	0.28505
Granulicatella	0.00356	0.00444	0.00330
Haemophilus	0.58685	1.18317	0.01648
Hallella	0.00783	0.00444	0.00659
Hespellia	0.00000	0.00000	0.00220
Howardella	0.00356	0.00111	0.00000
Intestinimonas	0.00000	0.00444	0.04778
Klebsiella	0.02137	0.32077	0.00000
Lachnospiracea_incertae_sedis	0.03703	0.60934	0.52122
Lactobacillus	0.40951	0.62821	0.08129
Leptotrichia	0.00356	0.00333	0.00055
Megamonas	0.95933	0.00000	0.31032
Megasphaera	0.00285	0.22420	0.11040
Methylobacterium	0.00356	0.00666	0.00000
Mitsuokella	0.00071	0.34851	0.01593
Neisseria	0.00855	0.03219	0.00989
Odoribacter	0.00285	0.00000	0.08568
Olsenella	0.00285	0.03996	0.01648
Oribacterium	0.00142	0.17648	0.00384

Genus	W_India_T60_57	W_India_T60_58	W_India_T60_59
**Oscillibacter**	0.00000	0.10766	0.32350
**Parabacteroides**	0.02778	0.00333	0.49816
**Paraprevotella**	0.00000	0.00000	0.50530
**Parasutterella**	0.00000	0.00000	0.00055
**Peptostreptococcus**	0.00000	0.00222	0.00110
**Prevotella**	72.01790	68.58500	66.37700
**Pseudoflavonifractor**	0.00000	0.00222	0.00165
**Pseudomonas**	0.00000	0.00000	0.00000
**Ralstonia**	0.00000	0.00000	0.00000
**Rhizobium**	0.00000	0.00000	0.00000
**Roseburia**	1.99201	3.58281	2.89556
**Rothia**	0.02920	0.01998	0.00330
**Ruminococcus**	0.00427	0.15428	0.60086
**Ruminococcus2**	0.02635	0.03663	0.09502
**Saccharibacteria_genera_incertae_sedis**	0.00783	0.00777	0.00275
**Slackia**	0.00000	0.00222	0.00604
**Solobacterium**	0.00000	0.00000	0.00000
**Streptococcus**	1.27697	0.12209	0.08348
**Streptophyta**	0.00570	0.00555	0.00769
**Succinivibrio**	0.00926	4.88474	2.71541
**Sutterella**	2.38443	0.00888	1.19788
**Turicibacter**	1.65941	0.26083	0.00000
**Veillonella**	0.09900	0.09434	0.06810
**Weissella**	0.00000	0.03996	0.00000

Genus	W_India_T60_6	W_India_T60_60	W_India_T60_61
**Acinetobacter**	0.00000	0.00000	0.00109
**Actinomyces**	0.00071	0.00739	0.00054
**Akkermansia**	0.00000	0.00046	0.00326
**Alistipes**	0.00000	0.01016	0.40680
**Allisonella**	0.00000	0.00000	0.00435
**Alloprevotella**	0.15286	2.46729	2.10417
**Anaerostipes**	0.00849	0.01016	0.00054
**Bacteroides**	0.44231	0.70435	0.74182
**Barnesiella**	0.00071	0.08452	0.04242
**Bifidobacterium**	0.83507	2.58784	0.70592
**Bilophila**	0.00000	0.00046	0.05112
**Blautia**	0.18258	0.10531	0.10986
**Butyricicoccus**	0.03468	0.03972	0.03535
**Butyricimonas**	0.00000	0.00647	0.03807
**Campylobacter**	0.00000	0.00139	0.00163
**Catenibacterium**	0.29015	0.47665	0.29912
**Clostridium_IV**	0.00000	0.16581	0.04188
**Clostridium_sensu_stricto**	3.10322	0.00046	0.07668
**Clostridium_XI**	0.73529	0.00092	0.04786
**Clostridium_XlVa**	1.16415	0.13995	0.20666
**Clostridium_XlVb**	0.05662	0.02448	0.03209
**Collinsella**	0.00212	0.09376	0.07342
**Comamonas**	0.00071	0.00046	0.03644
**Coprococcus**	0.00708	0.49697	0.26323
**Dialister**	2.35236	0.70342	2.06719
**Dorea**	0.10262	0.05773	0.20449
**Elusimicrobium**	0.00000	1.71075	0.00109
**Enterobacter**	0.00000	0.00000	0.00381
**Erysipelotrichaceae_incertae_sedis**	0.00637	0.00785	0.15554
**Escherichia_Shigella**	1.86335	0.00416	0.28824
**Faecalibacterium**	4.27656	11.56330	8.77399
**Flavonifractor**	0.00071	0.00000	0.00761
**Fusicatenibacter**	0.19461	0.02725	0.02121
**Fusobacterium**	0.00354	0.00277	0.00707
**Gemella**	0.00071	0.00139	0.00054
**Gemmiger**	0.01628	0.24156	0.39266
**Granulicatella**	0.00212	0.00092	0.00054
**Haemophilus**	0.06511	0.32654	0.04242
**Hallella**	0.00000	0.00739	0.00598
**Hespellia**	0.00000	0.00185	0.00054
**Howardella**	0.00000	0.00416	0.00761
**Intestinimonas**	0.00000	0.10069	0.03589
**Klebsiella**	0.00142	0.00924	0.50415
**Lachnospiracea_incertae_sedis**	0.25123	0.36811	0.16153
**Lactobacillus**	0.60083	0.90156	0.65371
**Leptotrichia**	0.00000	0.00046	0.00000
**Megamonas**	0.00000	0.01155	0.13651
**Megasphaera**	4.86607	0.00185	0.33719
**Methylobacterium**	0.00142	0.00600	0.00326
**Mitsuokella**	1.19387	0.15565	0.76411
**Neisseria**	0.00071	0.00416	0.00054
**Odoribacter**	0.00142	0.00046	0.08756
**Olsenella**	0.00000	0.08545	0.02121
**Oribacterium**	0.00354	0.00139	0.00109

Genus	W_India_T60_6	W_India_T60_60	W_India_T60_61
**Oscillibacter**	0.00071	0.04157	0.05874
**Parabacteroides**	0.00142	0.09237	0.22352
**Paraprevotella**	0.00071	0.14226	0.13433
**Parasutterella**	0.00000	0.01293	0.02611
**Peptostreptococcus**	0.01415	0.00046	0.00054
**Prevotella**	75.80480	67.91420	67.66840
**Pseudoflavonifractor**	0.00000	0.00369	0.00000
**Pseudomonas**	0.00000	0.00000	0.00000
**Ralstonia**	0.00000	0.00000	0.00000
**Rhizobium**	0.00000	0.00046	0.00000
**Roseburia**	0.99784	3.30604	3.74226
**Rothia**	0.00000	0.00831	0.00435
**Ruminococcus**	0.00142	0.40968	0.40463
**Ruminococcus2**	0.01486	0.04249	0.08430
**Saccharibacteria_genera_incertae_sedis**	0.02123	0.00323	0.00381
**Slackia**	0.00000	0.00000	0.00272
**Solobacterium**	0.00000	0.00000	0.00000
**Streptococcus**	0.13022	0.18937	0.31870
**Streptophyta**	0.06723	0.11824	0.06091
**Succinivibrio**	0.01911	0.01986	6.18144
**Sutterella**	0.02831	1.23180	0.97676
**Turicibacter**	0.00000	0.00323	0.01849
**Veillonella**	0.03397	0.04572	0.02447
**Weissella**	0.00000	0.00000	0.00707

Genus	W_India_T60_62	W_India_T60_63	W_India_T60_64
**Acinetobacter**	0.00000	0.00075	0.00061
**Actinomyces**	0.00181	0.00075	0.00061
**Akkermansia**	0.00453	0.00000	0.00000
**Alistipes**	0.39519	0.00299	0.00306
**Allisonella**	0.00634	0.00000	0.00000
**Alloprevotella**	4.93190	0.02240	0.01715
**Anaerostipes**	0.00091	0.00000	0.00122
**Bacteroides**	1.17198	0.88493	0.57076
**Barnesiella**	0.04979	0.00075	0.00245
**Bifidobacterium**	0.46580	0.22553	0.18005
**Bilophila**	0.01720	0.00000	0.00061
**Blautia**	0.10864	0.16504	0.17637
**Butyricicoccus**	0.05070	0.04481	0.02817
**Butyricimonas**	0.00905	0.14413	0.09799
**Campylobacter**	0.00045	0.00000	0.00122
**Catenibacterium**	0.14893	0.27855	0.42072
**Clostridium_IV**	0.02580	0.00000	0.00061
**Clostridium_sensu_stricto**	0.07741	0.03136	0.03123
**Clostridium_XI**	0.13671	0.12397	0.06736
**Clostridium_XlVa**	0.13399	0.39131	0.26823
**Clostridium_XlVb**	0.02444	0.01867	0.01715
**Collinsella**	0.07695	0.06796	0.04471
**Comamonas**	0.04481	0.00672	0.00367
**Coprococcus**	0.14667	1.26205	1.08335
**Dialister**	0.27477	0.43313	0.25048
**Dorea**	0.18424	0.13890	0.22782
**Elusimicrobium**	0.00136	0.00000	0.00061
**Enterobacter**	0.00226	0.00000	0.00000
**Erysipelotrichaceae_incertae_sedis**	0.29786	0.50407	0.24680
**Escherichia_Shigella**	0.25757	0.06273	0.03307
**Faecalibacterium**	12.41600	8.76117	7.22028
**Flavonifractor**	0.00362	0.00597	0.00306
**Fusicatenibacter**	0.02671	0.00000	0.02205
**Fusobacterium**	0.01086	0.00149	0.00000
**Gemella**	0.00136	0.00000	0.00061
**Gemmiger**	0.33679	0.33978	0.16229
**Granulicatella**	0.00091	0.00149	0.00061
**Haemophilus**	0.07243	0.17848	0.13718
**Hallella**	0.01992	0.00000	0.00000
**Hespellia**	0.00000	0.00000	0.00061
**Howardella**	0.00498	0.00000	0.00000
**Intestinimonas**	0.02082	0.02315	0.01654
**Klebsiella**	0.43231	0.01867	0.00796
**Lachnospiracea_incertae_sedis**	0.15074	0.19491	0.37051
**Lactobacillus**	0.39564	0.01270	0.00980
**Leptotrichia**	0.00091	0.00000	0.00000
**Megamonas**	0.08737	0.00075	0.00122
**Megasphaera**	0.11226	0.00149	0.00122
**Methylobacterium**	0.00317	0.00821	0.00000
**Mitsuokella**	0.31325	0.19939	0.18250
**Neisseria**	0.00317	0.00821	0.00429
**Odoribacter**	0.09733	0.00000	0.00061
**Olsenella**	0.01765	0.00075	0.00000
**Oribacterium**	0.00543	0.00000	0.00184

Genus	W_India_T60_62	W_India_T60_63	W_India_T60_64
**Oscillibacter**	0.07831	0.09036	0.02450
**Parabacteroides**	0.27794	0.28751	0.19107
**Paraprevotella**	0.23584	0.00000	0.00000
**Parasutterella**	0.00226	0.00149	0.00000
**Peptostreptococcus**	0.00136	0.00000	0.00000
**Prevotella**	64.42970	73.67620	78.51060
**Pseudoflavonifractor**	0.00136	0.00000	0.00000
**Pseudomonas**	0.00000	0.00000	0.00000
**Ralstonia**	0.00000	0.00000	0.00000
**Rhizobium**	0.00000	0.00448	0.00061
**Roseburia**	3.55078	9.36382	7.93864
**Rothia**	0.00407	0.00597	0.00245
**Ruminococcus**	0.91667	0.13069	0.11146
**Ruminococcus2**	0.07152	0.04929	0.04532
**Saccharibacteria_genera_incertae_sedis**	0.00679	0.00000	0.00061
**Slackia**	0.00000	0.00149	0.00061
**Solobacterium**	0.00000	0.00000	0.00000
**Streptococcus**	0.38885	0.15458	0.10411
**Streptophyta**	0.07786	0.00075	0.00000
**Succinivibrio**	5.37144	0.02390	0.00122
**Sutterella**	0.75099	1.44725	1.06191
**Turicibacter**	0.05885	0.10903	0.07349
**Veillonella**	0.03893	0.00523	0.01102
**Weissella**	0.00996	0.00000	0.00061

Genus	W_India_T60_65	W_India_T60_66	W_India_T60_67
**Acinetobacter**	0.00000	0.00000	0.00000
**Actinomyces**	0.00254	0.00138	0.00277
**Akkermansia**	0.88027	0.00000	0.00000
**Alistipes**	0.34473	0.18803	0.00000
**Allisonella**	0.00000	0.00000	0.00000
**Alloprevotella**	0.00763	11.19180	3.06777
**Anaerostipes**	0.00000	0.00138	0.00553
**Bacteroides**	7.66041	0.32006	0.24066
**Barnesiella**	0.00000	0.00899	0.00000
**Bifidobacterium**	0.04198	0.57238	0.44537
**Bilophila**	0.08650	0.04286	0.00000
**Blautia**	0.07124	0.25024	0.15214
**Butyricicoccus**	0.09541	0.19840	0.04149
**Butyricimonas**	0.24169	0.00553	0.00000
**Campylobacter**	0.00254	0.00000	0.00000
**Catenibacterium**	0.00000	0.88898	0.14938
**Clostridium_IV**	0.75052	0.08848	0.04703
**Clostridium_sensu_stricto**	1.04055	0.03802	0.25173
**Clostridium_XI**	0.00254	0.08710	0.27109
**Clostridium_XlVa**	0.33583	0.17628	0.11895
**Clostridium_XlVb**	0.59151	0.03180	0.01936
**Collinsella**	0.02035	0.11821	0.01936
**Comamonas**	0.00000	0.01452	0.09405
**Coprococcus**	0.08141	0.83714	0.18534
**Dialister**	0.06869	0.24817	1.61549
**Dorea**	0.36381	0.24679	0.04979
**Elusimicrobium**	0.00000	3.98175	0.00000
**Enterobacter**	0.00127	0.07604	0.00000
**Erysipelotrichaceae_incertae_sedis**	0.00000	0.00000	0.50069
**Escherichia_Shigella**	3.99685	0.38988	0.61964
**Faecalibacterium**	8.53432	6.71989	7.06777
**Flavonifractor**	0.02290	0.00207	0.00000
**Fusicatenibacter**	0.02417	0.01244	0.00553
**Fusobacterium**	0.00382	0.00000	0.00830
**Gemella**	0.00000	0.00622	0.00000
**Gemmiger**	0.37653	0.36085	0.15491
**Granulicatella**	0.00000	0.00415	0.00553
**Haemophilus**	0.58261	0.35186	0.28492
**Hallella**	0.01018	0.00622	0.00000
**Hespellia**	0.00382	0.00415	0.00000
**Howardella**	0.00254	0.00000	0.01107
**Intestinimonas**	0.16410	0.01866	0.07746
**Klebsiella**	1.00494	0.35463	0.53942
**Lachnospiracea_incertae_sedis**	0.28113	0.53228	0.01660
**Lactobacillus**	0.01526	0.21775	0.05256
**Leptotrichia**	0.00000	0.00000	0.00000
**Megamonas**	0.00127	0.00138	0.59751
**Megasphaera**	0.00000	0.45693	0.84647
**Methylobacterium**	0.00254	0.00553	0.00000
**Mitsuokella**	0.00000	0.00000	0.08852
**Neisseria**	0.00254	0.00069	0.00830
**Odoribacter**	2.31644	0.04770	0.00000
**Olsenella**	0.00000	0.03802	0.01383
**Oribacterium**	0.01781	0.00622	0.00000

Genus	W_India_T60_65	W_India_T60_66	W_India_T60_67
**Oscillibacter**	0.12212	0.11475	1.34440
**Parabacteroides**	3.24251	0.62215	0.00830
**Paraprevotella**	0.16410	0.03042	0.00000
**Parasutterella**	0.00636	0.00691	0.00000
**Peptostreptococcus**	0.00127	0.00207	0.00553
**Prevotella**	59.73010	64.68060	80.09680
**Pseudoflavonifractor**	0.00890	0.00000	0.00553
**Pseudomonas**	0.00000	0.00000	0.00000
**Ralstonia**	0.00000	0.00000	0.00000
**Rhizobium**	0.00000	0.00000	0.00000
**Roseburia**	2.78711	4.08337	0.53389
**Rothia**	0.00254	0.00415	0.00553
**Ruminococcus**	2.72732	0.34564	0.01383
**Ruminococcus2**	0.19336	0.09194	0.00553
**Saccharibacteria_genera_incertae_sedis**	0.00763	0.00138	0.00000
**Slackia**	0.00000	0.00277	0.00000
**Solobacterium**	0.00000	0.00000	0.00000
**Streptococcus**	0.11830	0.07051	0.18534
**Streptophyta**	0.00000	0.00000	0.00553
**Succinivibrio**	0.01145	0.00277	0.02213
**Sutterella**	0.18572	0.47836	0.01936
**Turicibacter**	0.00000	0.16038	0.00830
**Veillonella**	0.12848	0.00968	0.03596
**Weissella**	0.00000	0.00000	0.00000

Genus	W_India_T60_68	W_India_T60_69	W_India_T60_7
**Acinetobacter**	0.00159	0.00256	0.00130
**Actinomyces**	0.00478	0.00256	0.00000
**Akkermansia**	0.00000	0.00000	0.00000
**Alistipes**	0.16969	0.00128	0.53283
**Allisonella**	0.00000	0.00000	0.00000
**Alloprevotella**	14.56170	10.33650	0.00130
**Anaerostipes**	0.00239	0.01409	0.00520
**Bacteroides**	2.67844	0.01793	44.79060
**Barnesiella**	0.01115	0.00000	0.73687
**Bifidobacterium**	6.24915	1.28486	0.37818
**Bilophila**	0.02629	0.00000	0.02859
**Blautia**	0.41029	0.09992	0.46526
**Butyricicoccus**	0.23104	0.01793	0.01430
**Butyricimonas**	0.00000	0.00128	0.42627
**Campylobacter**	0.00478	0.00128	0.00000
**Catenibacterium**	2.72544	1.46676	0.18064
**Clostridium_IV**	0.09719	0.00000	0.16895
**Clostridium_sensu_stricto**	0.03107	0.37790	0.00130
**Clostridium_XI**	0.02231	0.45732	0.00000
**Clostridium_XlVa**	0.07887	0.12554	0.34959
**Clostridium_XlVb**	0.05656	0.00897	0.05198
**Collinsella**	0.10835	0.08583	0.01170
**Comamonas**	0.07568	0.00000	0.00000
**Coprococcus**	1.10499	0.03971	0.03249
**Dialister**	1.70569	2.14442	0.16895
**Dorea**	0.45650	0.12042	0.31320
**Elusimicrobium**	0.00080	0.00769	0.00000
**Enterobacter**	0.00000	0.00000	0.00000
**Erysipelotrichaceae_incertae_sedis**	0.47243	0.03587	0.01170
**Escherichia_Shigella**	0.39037	3.40750	0.26382
**Faecalibacterium**	16.49600	1.20672	8.98411
**Flavonifractor**	0.00239	0.00000	0.01560
**Fusicatenibacter**	0.07170	0.01922	0.22093
**Fusobacterium**	0.00000	0.01537	0.00130
**Gemella**	0.00080	0.00128	0.00000
**Gemmiger**	0.49155	0.05765	0.43147
**Granulicatella**	0.00080	0.00256	0.00000
**Haemophilus**	0.17527	0.86981	0.00910
**Hallella**	0.01514	0.00000	0.00000
**Hespellia**	0.00398	0.00000	0.00000
**Howardella**	0.00000	0.02050	0.00000
**Intestinimonas**	0.08206	0.00769	0.08057
**Klebsiella**	0.00717	0.03715	0.01819
**Lachnospiracea_incertae_sedis**	0.38161	0.07046	0.05588
**Lactobacillus**	0.33859	2.43393	0.06498
**Leptotrichia**	0.00000	0.00384	0.00000
**Megamonas**	0.00000	0.00000	0.10527
**Megasphaera**	0.00080	0.00384	0.05198
**Methylobacterium**	0.00558	0.00769	0.00000
**Mitsuokella**	0.00159	3.84561	0.09227
**Neisseria**	0.00478	0.03203	0.00000
**Odoribacter**	0.04780	0.00128	0.20144
**Olsenella**	0.10915	0.01025	0.00000
**Oribacterium**	0.00080	0.00128	0.00000

Genus	W_India_T60_68	W_India_T60_69	W_India_T60_7
**Oscillibacter**	0.05497	0.01281	0.11956
**Parabacteroides**	0.23183	0.00256	1.29700
**Paraprevotella**	0.11393	0.00256	0.04809
**Parasutterella**	1.11933	0.00000	0.00000
**Peptostreptococcus**	0.00000	0.00512	0.00000
**Prevotella**	37.24320	65.32160	29.98820
**Pseudoflavonifractor**	0.00159	0.00000	0.00390
**Pseudomonas**	0.00000	0.00000	0.00000
**Ralstonia**	0.00000	0.00000	0.00000
**Rhizobium**	0.00000	0.00128	0.00000
**Roseburia**	4.94180	1.34122	7.77548
**Rothia**	0.00239	0.01025	0.00000
**Ruminococcus**	1.29699	0.00512	0.02339
**Ruminococcus2**	0.08763	0.00641	0.13516
**Saccharibacteria_genera_incertae_sedis**	0.00717	0.00256	0.00130
**Slackia**	0.00319	0.00256	0.00000
**Solobacterium**	0.00000	0.00000	0.00000
**Streptococcus**	4.82628	2.90022	0.92661
**Streptophyta**	0.00159	0.00769	0.00260
**Succinivibrio**	0.00398	0.01537	0.00130
**Sutterella**	0.00000	1.11577	0.04029
**Turicibacter**	0.05736	0.00256	0.00390
**Veillonella**	0.07807	0.48679	0.00650
**Weissella**	0.00000	0.00256	0.00000

Genus	W_India_T60_70	W_India_T60_71	W_India_T60_72
**Acinetobacter**	0.00113	0.03989	0.00000
**Actinomyces**	0.00113	0.00000	0.00174
**Akkermansia**	4.54096	0.00886	0.00000
**Alistipes**	0.72777	0.16546	0.06893
**Allisonella**	0.00270	0.00000	0.00087
**Alloprevotella**	0.69106	8.11210	4.17565
**Anaerostipes**	0.01892	0.00591	0.00523
**Bacteroides**	2.24728	0.97357	0.07591
**Barnesiella**	0.02343	0.14035	0.03228
**Bifidobacterium**	1.48843	2.15840	1.27555
**Bilophila**	0.07456	0.05762	0.00436
**Blautia**	0.23336	0.38559	0.13523
**Butyricicoccus**	0.04640	0.21274	0.13087
**Butyricimonas**	0.12817	0.12705	0.07154
**Campylobacter**	0.00023	0.00148	0.00262
**Catenibacterium**	0.47302	0.00739	0.21288
**Clostridium_IV**	0.09956	0.55548	0.09772
**Clostridium_sensu_stricto**	0.03221	0.08421	0.00000
**Clostridium_XI**	0.00698	0.02364	0.00000
**Clostridium_XlVa**	0.09438	0.25706	0.12564
**Clostridium_XlVb**	0.05519	0.07978	0.02268
**Collinsella**	0.04482	0.10194	0.01309
**Comamonas**	0.00631	0.00148	0.00087
**Coprococcus**	0.21173	0.96914	0.10382
**Dialister**	0.19484	0.26592	0.29751
**Dorea**	0.21579	0.48900	0.20416
**Elusimicrobium**	0.00090	0.00000	0.00000
**Enterobacter**	0.00000	0.00443	0.00000
**Erysipelotrichaceae_incertae_sedis**	0.23471	3.05072	0.20329
**Escherichia_Shigella**	0.05158	0.23933	0.00436
**Faecalibacterium**	14.60340	14.63900	8.71424
**Flavonifractor**	0.32481	0.14921	0.00611
**Fusicatenibacter**	0.06510	0.11376	0.00785
**Fusobacterium**	0.00135	0.00295	0.00262
**Gemella**	0.00068	0.00443	0.00611
**Gemmiger**	0.16691	0.31172	0.09335
**Granulicatella**	0.00135	0.00148	0.00262
**Haemophilus**	0.06847	0.00295	0.02181
**Hallella**	0.01284	0.00886	0.00523
**Hespellia**	0.00068	0.00295	0.00436
**Howardella**	0.00023	0.00000	0.00349
**Intestinimonas**	0.05248	0.16990	0.03054
**Klebsiella**	0.02523	0.65446	0.00087
**Lachnospiracea_incertae_sedis**	0.14889	2.10522	0.38563
**Lactobacillus**	0.71628	0.39002	0.32281
**Leptotrichia**	0.00180	0.00148	0.00349
**Megamonas**	0.00360	0.00000	0.00523
**Megasphaera**	0.34463	0.00148	0.01221
**Methylobacterium**	0.00180	0.00443	0.00087
**Mitsuokella**	0.16713	0.00443	0.00087
**Neisseria**	0.00203	0.00886	0.00262
**Odoribacter**	0.11397	0.19501	0.04450
**Olsenella**	0.01149	0.01034	0.00174
**Oribacterium**	0.00135	0.01034	0.00087

Genus	W_India_T60_70	W_India_T60_71	W_India_T60_72
**Oscillibacter**	0.37571	0.43729	0.02617
**Parabacteroides**	0.78904	0.61310	0.08812
**Paraprevotella**	0.05023	0.73424	0.14658
**Parasutterella**	0.01126	0.00739	0.00087
**Peptostreptococcus**	0.00023	0.00295	0.00174
**Prevotella**	64.98660	49.76290	52.61780
**Pseudoflavonifractor**	0.01284	0.00000	0.00960
**Pseudomonas**	0.00000	0.00000	0.00000
**Ralstonia**	0.00000	0.00000	0.00000
**Rhizobium**	0.00023	0.00000	0.00000
**Roseburia**	1.99816	6.75885	1.62629
**Rothia**	0.00608	0.00295	0.00262
**Ruminococcus**	1.63146	3.00049	0.38912
**Ruminococcus2**	0.03739	0.11376	0.07591
**Saccharibacteria_genera_incertae_sedis**	0.00360	0.00295	0.00000
**Slackia**	0.00338	0.01477	0.00000
**Solobacterium**	0.00000	0.00000	0.00000
**Streptococcus**	0.10249	0.25115	0.03228
**Streptophyta**	0.04618	0.66333	0.00087
**Succinivibrio**	0.81179	0.00443	27.82050
**Sutterella**	0.14326	0.01182	0.15879
**Turicibacter**	0.01104	0.02807	0.00000
**Veillonella**	0.02748	0.02511	0.00785
**Weissella**	0.00090	0.00148	0.00000

Genus	W_India_T60_73	W_India_T60_74	W_India_T60_75
Acinetobacter	0.00236	0.01275	0.00000
Actinomyces	0.00000	0.02550	0.05793
Akkermansia	0.00236	0.00000	0.00000
Alistipes	0.28660	1.16786	0.00000
Allisonella	0.00473	0.00000	0.00341
Alloprevotella	0.13946	0.74705	11.22540
Anaerostipes	0.00709	0.00000	0.00886
Bacteroides	1.28527	1.68218	0.33597
Barnesiella	0.00118	0.00531	0.01908
Bifidobacterium	3.07638	2.36334	0.05384
Bilophila	0.07682	0.12433	0.06270
Blautia	0.24937	0.40275	0.44773
Butyricicoccus	0.02659	0.06801	0.13766
Butyricimonas	0.10755	0.00000	0.00068
Campylobacter	0.00000	0.01913	0.00545
Catenibacterium	0.41011	1.14767	0.32847
Clostridium_IV	0.01536	0.67160	0.37959
Clostridium_sensu_stricto	0.15423	0.00850	0.27055
Clostridium_XI	0.57616	0.02444	0.15197
Clostridium_XlVa	0.25587	0.14665	0.16696
Clostridium_XlVb	0.02836	0.02550	0.13698
Collinsella	0.53538	0.10308	0.13289
Comamonas	0.00000	0.00000	0.22216
Coprococcus	0.88344	2.66514	0.59153
Dialister	1.72256	1.32300	0.30394
Dorea	0.18082	0.46544	0.45523
Elusimicrobium	0.00118	0.00000	0.00000
Enterobacter	0.00000	0.00000	0.00204
Erysipelotrichaceae_incertae_sedis	0.69848	0.48138	0.30735
Escherichia_Shigella	0.84089	0.22316	0.09336
Faecalibacterium	13.91930	12.63070	9.36084
Flavonifractor	0.01241	0.00213	0.11858
Fusicatenibacter	0.03900	0.28054	0.05861
Fusobacterium	0.00118	0.12221	0.03953
Gemella	0.00118	0.00850	0.00818
Gemmiger	0.45561	0.62059	0.32643
Granulicatella	0.00000	0.01488	0.02181
Haemophilus	0.09396	0.21784	0.12471
Hallella	0.01300	0.00956	0.00681
Hespellia	0.00000	0.00106	0.00341
Howardella	0.00000	0.07332	0.00750
Intestinimonas	0.02127	0.10095	0.06747
Klebsiella	0.03073	0.00213	0.04430
Lachnospiracea_incertae_sedis	0.35338	0.32411	0.95612
Lactobacillus	0.68607	0.27417	0.05179
Leptotrichia	0.00118	0.02657	0.08246
Megamonas	0.06382	0.00106	0.00068
Megasphaera	0.21746	1.93191	0.00409
Methylobacterium	0.01714	0.01594	0.00750
Mitsuokella	0.54247	0.57596	0.18468
Neisseria	0.00118	0.36449	0.11245
Odoribacter	0.21864	0.16684	0.05247
Olsenella	0.10282	0.22103	0.00954
Oribacterium	0.00473	0.00744	0.02385

Genus	W_India_T60_73	W_India_T60_74	W_India_T60_75
**Oscillibacter**	0.07918	0.05101	0.08996
**Parabacteroides**	0.24228	0.88625	0.08314
**Paraprevotella**	0.37820	0.07970	0.01090
**Parasutterella**	0.00591	0.00106	0.00681
**Peptostreptococcus**	0.00000	0.00106	0.01295
**Prevotella**	45.04120	58.90290	52.15450
**Pseudoflavonifractor**	0.00000	0.00213	0.00341
**Pseudomonas**	0.00000	0.00000	0.00000
**Ralstonia**	0.00000	0.00000	0.00000
**Rhizobium**	0.00118	0.00000	0.00068
**Roseburia**	4.67957	6.86793	6.29621
**Rothia**	0.00473	0.06057	0.13357
**Ruminococcus**	0.44261	0.29436	2.47923
**Ruminococcus2**	0.05496	0.02019	0.17923
**Saccharibacteria_genera_incertae_sedis**	0.00236	0.02444	0.05793
**Slackia**	0.00591	0.01275	0.00273
**Solobacterium**	0.00000	0.00000	0.00000
**Streptococcus**	0.84030	0.18490	0.30326
**Streptophyta**	0.00945	0.00531	0.00204
**Succinivibrio**	18.73950	0.00744	10.02660
**Sutterella**	0.91830	0.13602	0.20785
**Turicibacter**	0.04255	0.00106	0.04770
**Veillonella**	0.08214	0.14027	0.06542
**Weissella**	0.00118	0.00000	0.00068

Genus	W_India_T60_76	W_India_T60_77	W_India_T60_78
**Acinetobacter**	0.00157	0.00000	0.00042
**Actinomyces**	0.00565	0.00691	0.01127
**Akkermansia**	0.00000	0.07777	0.05675
**Alistipes**	0.00534	0.16936	0.06865
**Allisonella**	0.02010	0.00000	0.00209
**Alloprevotella**	5.66428	0.01383	0.53853
**Anaerostipes**	0.00031	0.00864	0.00104
**Bacteroides**	0.34795	1.37389	0.39957
**Barnesiella**	0.00000	0.00691	0.01022
**Bifidobacterium**	1.96650	0.74138	0.99381
**Bilophila**	0.00031	0.04666	0.00584
**Blautia**	0.13127	0.47179	0.24329
**Butyricicoccus**	0.05464	0.05530	0.60217
**Butyricimonas**	0.00063	0.00346	0.00855
**Campylobacter**	0.00722	0.01383	0.00605
**Catenibacterium**	1.33120	0.00173	0.23682
**Clostridium_IV**	0.00126	0.02074	0.01419
**Clostridium_sensu_stricto**	0.48927	0.06567	0.87237
**Clostridium_XI**	0.10583	0.15035	0.49784
**Clostridium_XlVa**	0.07066	0.36464	0.05279
**Clostridium_XlVb**	0.00408	0.10023	0.01273
**Collinsella**	0.03863	0.08814	0.02024
**Comamonas**	0.00000	0.00000	0.00981
**Coprococcus**	0.15828	0.40785	0.09055
**Dialister**	0.54580	0.21084	0.85276
**Dorea**	0.18403	0.44241	0.14334
**Elusimicrobium**	0.00031	0.00173	0.00271
**Enterobacter**	0.24872	0.00000	0.01940
**Erysipelotrichaceae_incertae_sedis**	0.11086	0.00346	0.11643
**Escherichia_Shigella**	0.27887	0.50462	0.12164
**Faecalibacterium**	5.34114	13.69050	9.13873
**Flavonifractor**	0.00063	0.00691	0.02462
**Fusicatenibacter**	0.03737	0.07777	0.04736
**Fusobacterium**	0.01476	0.01901	0.00104
**Gemella**	0.00534	0.00346	0.00209
**Gemmiger**	0.12405	0.41649	0.19300
**Granulicatella**	0.01162	0.00518	0.00417
**Haemophilus**	0.62964	0.16763	3.17901
**Hallella**	0.00879	0.00346	0.02212
**Hespellia**	0.00408	0.00000	0.00021
**Howardella**	0.01507	0.00000	0.02587
**Intestinimonas**	0.01853	0.06913	0.03338
**Klebsiella**	4.80885	0.76903	1.58283
**Lachnospiracea_incertae_sedis**	0.45316	0.29897	0.18528
**Lactobacillus**	0.83722	0.22121	3.60049
**Leptotrichia**	0.00628	0.00173	0.00042
**Megamonas**	0.00000	0.00691	0.06176
**Megasphaera**	0.57500	0.00518	2.31541
**Methylobacterium**	0.00283	0.00346	0.00250
**Mitsuokella**	0.52193	0.16072	0.07073
**Neisseria**	0.12750	0.03975	0.03693
**Odoribacter**	0.00031	0.04839	0.00647
**Olsenella**	0.13252	0.01728	0.03672
**Oribacterium**	0.07191	0.00000	0.00793

Genus	W_India_T60_76	W_India_T60_77	W_India_T60_78
**Oscillibacter**	0.01288	0.38020	0.18069
**Parabacteroides**	0.00377	0.27305	0.08263
**Paraprevotella**	0.00063	0.02938	0.03213
**Parasutterella**	0.00000	0.01728	0.00417
**Peptostreptococcus**	0.00157	0.00000	0.00939
**Prevotella**	66.87570	65.88270	62.30750
**Pseudoflavonifractor**	0.00251	0.00346	0.00063
**Pseudomonas**	0.00000	0.00000	0.00000
**Ralstonia**	0.00000	0.00000	0.00000
**Rhizobium**	0.00063	0.00000	0.00146
**Roseburia**	5.05568	5.33137	0.85067
**Rothia**	0.07474	0.00691	0.02274
**Ruminococcus**	0.02858	0.11406	0.10641
**Ruminococcus2**	0.04114	0.14517	0.02045
**Saccharibacteria_genera_incertae_sedis**	0.03172	0.01210	0.01398
**Slackia**	0.00659	0.00000	0.00188
**Solobacterium**	0.00000	0.00000	0.00000
**Streptococcus**	0.35643	0.59967	4.36269
**Streptophyta**	0.03329	0.01210	0.03818
**Succinivibrio**	0.04459	4.97192	1.63541
**Sutterella**	0.88684	0.57548	2.07796
**Turicibacter**	0.19439	0.06567	0.78683
**Veillonella**	0.34010	0.01728	0.62929
**Weissella**	0.11494	0.05012	0.15920

Genus	W_India_T60_79	W_India_T60_8	W_India_T60_80
**Acinetobacter**	0.00000	0.00000	0.00043
**Actinomyces**	0.00000	0.00000	0.00258
**Akkermansia**	0.04872	0.00000	0.19120
**Alistipes**	0.26799	0.00445	0.03144
**Allisonella**	0.00348	0.00297	0.00388
**Alloprevotella**	0.42112	18.14600	0.01163
**Anaerostipes**	0.00348	0.00000	0.00172
**Bacteroides**	3.57081	0.14682	0.74154
**Barnesiella**	0.02088	0.00000	0.00000
**Bifidobacterium**	1.75408	0.60655	0.99560
**Bilophila**	0.05917	0.00297	0.00086
**Blautia**	0.43852	0.07563	0.11196
**Butyricicoccus**	0.00696	0.01780	0.04177
**Butyricimonas**	0.11833	0.00000	0.00517
**Campylobacter**	0.00000	0.00148	0.00904
**Catenibacterium**	0.23318	0.44193	0.79752
**Clostridium_IV**	0.28887	0.00000	0.05598
**Clostridium_sensu_stricto**	0.06613	0.37520	0.20239
**Clostridium_XI**	0.03828	0.33219	0.11928
**Clostridium_XlVa**	0.09397	0.37075	0.10636
**Clostridium_XlVb**	0.01044	0.00593	0.01981
**Collinsella**	0.35151	0.09195	0.00904
**Comamonas**	0.00000	0.00000	0.00043
**Coprococcus**	0.32367	0.04597	0.45948
**Dialister**	0.66474	0.30846	0.94479
**Dorea**	0.12877	0.04004	0.10421
**Elusimicrobium**	0.00000	0.00000	0.00000
**Enterobacter**	0.00000	0.00000	0.03144
**Erysipelotrichaceae_incertae_sedis**	0.19142	0.08453	0.03445
**Escherichia_Shigella**	0.40720	0.18093	0.07191
**Faecalibacterium**	15.48390	5.94830	13.92040
**Flavonifractor**	0.01044	0.00000	0.00000
**Fusicatenibacter**	0.02436	0.00000	0.02885
**Fusobacterium**	0.00000	0.00000	0.04909
**Gemella**	0.00000	0.00000	0.00646
**Gemmiger**	0.27495	0.16313	0.23814
**Granulicatella**	0.00696	0.00000	0.00172
**Haemophilus**	0.09745	0.02818	0.21015
**Hallella**	0.01044	0.00741	0.00301
**Hespellia**	0.00000	0.00000	0.00129
**Howardella**	0.00000	0.00000	0.00000
**Intestinimonas**	0.01740	0.01335	0.01206
**Klebsiella**	0.12877	0.01631	0.53311
**Lachnospiracea_incertae_sedis**	0.15313	0.05042	0.38239
**Lactobacillus**	0.33063	0.14830	1.48135
**Leptotrichia**	0.00696	0.00000	0.03574
**Megamonas**	0.10093	0.00445	0.07837
**Megasphaera**	0.34803	0.68366	0.01895
**Methylobacterium**	0.01044	0.00445	0.00301
**Mitsuokella**	0.42808	1.59422	0.00861
**Neisseria**	0.00000	0.00000	0.24933
**Odoribacter**	0.12181	0.00000	0.01809
**Olsenella**	0.05569	0.00148	0.00431
**Oribacterium**	0.00348	0.00000	0.01206

Genus	W_India_T60_79	W_India_T60_8	W_India_T60_80
**Oscillibacter**	0.08005	0.04004	0.02325
**Parabacteroides**	0.45592	0.02966	0.15933
**Paraprevotella**	0.21578	0.00741	0.00043
**Parasutterella**	0.03480	0.00000	0.01809
**Peptostreptococcus**	0.00696	0.00000	0.00388
**Prevotella**	47.07830	65.75310	69.62030
**Pseudoflavonifractor**	0.00348	0.00148	0.00344
**Pseudomonas**	0.00000	0.00000	0.00000
**Ralstonia**	0.00000	0.00000	0.00000
**Rhizobium**	0.00000	0.00000	0.00043
**Roseburia**	3.43507	2.37280	2.38566
**Rothia**	0.00000	0.00445	0.02325
**Ruminococcus**	0.73783	0.04301	0.17268
**Ruminococcus2**	0.12529	0.01186	0.02067
**Saccharibacteria_genera_incertae_sedis**	0.01044	0.00297	0.00000
**Slackia**	0.01044	0.00000	0.00129
**Solobacterium**	0.00000	0.00000	0.00000
**Streptococcus**	0.20534	0.12012	0.12876
**Streptophyta**	0.00348	0.00148	3.96304
**Succinivibrio**	19.75430	0.01631	0.01507
**Sutterella**	0.48725	1.27093	0.71441
**Turicibacter**	0.02088	0.00445	0.19593
**Veillonella**	0.11137	0.01186	0.04823
**Weissella**	0.00000	0.00148	0.00043

Genus	W_India_T60_9
Acinetobacter	0.00080
Actinomyces	0.00637
Akkermansia	0.00000
Alistipes	0.00000
Allisonella	0.00000
Alloprevotella	0.13942
Anaerostipes	0.01593
Bacteroides	10.15960
Barnesiella	0.00080
Bifidobacterium	9.89189
Bilophila	0.00000
Blautia	0.27725
Butyricicoccus	0.06533
Butyricimonas	0.00000
Campylobacter	0.00000
Catenibacterium	0.01753
Clostridium_IV	0.00000
Clostridium_sensu_stricto	0.73616
Clostridium_XI	0.56566
Clostridium_XlVa	0.74173
Clostridium_XlVb	0.03904
Collinsella	0.00398
Comamonas	0.00000
Coprococcus	0.00398
Dialister	0.23981
Dorea	0.27646
Elusimicrobium	0.00000
Enterobacter	0.23104
Erysipelotrichaceae_incertae_sedis	0.00319
Escherichia_Shigella	1.10822
Faecalibacterium	22.22810
Flavonifractor	0.00159
Fusicatenibacter	0.33063
Fusobacterium	0.20396
Gemella	0.00000
Gemmiger	0.21989
Granulicatella	0.00558
Haemophilus	0.19758
Hallella	0.00000
Hespellia	0.00080
Howardella	0.00000
Intestinimonas	0.00000
Klebsiella	1.26437
Lachnospiracea_incertae_sedis	0.72341
Lactobacillus	4.30061
Leptotrichia	0.00000
Megamonas	7.12732
Megasphaera	6.19836
Methylobacterium	0.00637
Mitsuokella	0.04780
Neisseria	0.00159
Odoribacter	0.00000
Olsenella	0.00080
Oribacterium	0.00478

Genus	W_India_T60_9
**Oscillibacter**	0.00080
**Parabacteroides**	0.00319
**Paraprevotella**	0.00239
**Parasutterella**	0.00000
**Peptostreptococcus**	0.01753
**Prevotella**	5.41281
**Pseudoflavonifractor**	0.00000
**Pseudomonas**	0.00000
**Ralstonia**	0.00000
**Rhizobium**	0.00080
**Roseburia**	18.87790
**Rothia**	0.00159
**Ruminococcus**	0.00797
**Ruminococcus2**	0.10676
**Saccharibacteria_genera_incertae_sedis**	0.02310
**Slackia**	0.00000
**Solobacterium**	0.00000
**Streptococcus**	0.11632
**Streptophyta**	0.01514
**Succinivibrio**	0.00478
**Sutterella**	6.45490
**Turicibacter**	0.20157
**Veillonella**	0.06135
**Weissella**	0.00000
