# Supplementary material for: A snapshot of gut microbiota of an adult urban population from Western region of India
Source: PLoS One. 2018 Apr 6;13(4):e0195643. doi: 10.1371/journal.pone.0195643 (PMC5889170; doi:10.1371/journal.pone.0195643)
Supplement: S3 Table — (PDF) [file pone.0195643.s009.pdf]

S3 Table: Details of various studies (and corresponding number of samples) that were used for comparative analysis with Indian microbiome samples obtained in the present study

**Study Tag**	**Number of Samples**	**16S Variable region**	**Reference**
Japan_16	16*	V1-V2	Kato *et al.*, 2013
Japan_18	18*	V1-V2	Kim *et al.*, 2014
China	81*	V3	Xiao *et al.*, 2013
USA	80$	V1-V3 & V3-V5	Turnbaugh *et al.*, 2007
Finland	54^	V1-V3	Scheperjans *et al.*, 2015
India_18	18#	V3	Bhute *et al.*, 2016
Present Study	160	V3-V4	-

* Only those samples which were taken from healthy subjects (prior to administration of any form of pre/probiotics) were considered

$ In all studies (including this one), only those samples were considered that had a minimum of 1000 assigned sequences

^ In this study, only those samples which were taken from healthy (control) subjects were considered for analysis

18 samples whose metadata clearly indicated the subjects as 'healthy' were only considered.
