# Supplementary material for: A snapshot of gut microbiota of an adult urban population from Western region of India
Source: PLoS One. 2018 Apr 6;13(4):e0195643. doi: 10.1371/journal.pone.0195643 (PMC5889170; doi:10.1371/journal.pone.0195643)
Supplement: S4 Table — List of differentially abundant pathways (identified between Cluster-1a, Cluster-1b and Cluster-2). Significantly different pathways were identified using Kruskal-Wallis rank sum test (with Benjamini-Hochberg corrected p-values < 0.001 at a False Discovery Rate of 0.0001) coupled with a bootstrap approach. Pathways with significantly different median abundances in at least 99% of iterations are shown in this table. For ease of readers, the corresponding pathway classes are also indicated. The last column titled ‘Vikodak Pathway Exclusion Cut-off (PEC) threshold’ indicates the PEC value thresholds at which the pathway was reported by Vikodak. Green indicates presence and red indicates absence. (PDF) [file pone.0195643.s010.pdf]

S4 Table: List of differentially abundant pathways (identified between Cluster-1a, Cluster-1b and Cluster-2). Significantly different pathways were identified using Kruskal-Wallis rank sum test (with Benjamini-Hochberg corrected p-values < 0.001 at a False Discovery Rate of 0.0001) coupled with a bootstrap approach. Pathways with significantly different median abundances in at least 99% of iterations are shown in this table. For ease of readers, the corresponding pathway classes are also indicated. The last column titled ‘Vikodak Pathway Exclusion Cut-off (PEC) threshold’ indicates the PEC value thresholds at which the pathway was reported by Vikodak. Green indicates presence and red indicates absence.

**Pathway Class**	**Pathways that significantly differentiate between the three clusters**	**Median Pathway Abundance (at PEC 70)**			**Vikodak Pathway Exclusion Cut-off (PEC) threshold**				
		**Cluster 1a**	**Cluster 1b**	**Cluster 2**	**50**	**60**	**70**	**80**	**90**
Membrane transport	Phosphotransferase system PTS	1227	1147	742					
Carbohydrate metabolism	Glycolysis or Gluconeogenesis	40270	49509	29060					
Nucleotide metabolism	Pyrimidine metabolism	37800	46761	32265					
Amino acid metabolism	Alanine aspartate and glutamate	28782	34174	18836					
Carbohydrate	Pyruvate	20237	24359	14641					
Energy metabolism	Carbon fixation pathways in prokaryotes	19114	23560	13591					
Carbohydrate	Butanoate	18008	21509	11308					
Translation	Aminoacyl tRNA	16815	19736	15286					
Energy metabolism	Carbon fixation in photosynthetic organisms	15203	19199	10202					
Metabolism of cofactors and vitmains	Pantothenate and CoA biosynthesis	14256	18111	11505					
Carbohydrate metabolism	Citrate cycle TCA cycle	13957	17563	9960					
Carbohydrate metabolism	Pentose phosphate pathway	13436	17101	9502					

Energy metabolism	Oxidative phosphorylation	12633	16007	9807
Glycan biosynthesis and metabolism	Other glycan degradation	10308	14466	4153
Metabolism of cofactors and vitamins	Folate biosynthesis	10168	13260	8159
Glycan biosynthesis and metabolism	Peptidoglycan biosynthesis	10350	12637	8623
Metabolism of other amino acids	Selenocompound metabolism	9603	12268	7111
Lipid metabolism	Fatty acid biosynthesis	9087	12131	5429
Metabolism of cofactors and vitamins	One carbon pool by folate	9492	11976	7798
Folding, sorting and degradation	RNA degradation	10297	11228	9104
Glycan biosynthesis and [illegible]	Lipopolysaccharide biosynthesis	8089	10602	6178
Replication and repair	Mismatch repair	8610	10568	7440
Replication and repair	DNA replication	7660	9503	6522
Cell growth and death	Cell cycle Caulobacter	7081	8942	5820
Metabolism of terpenoids and polyketides	Tetracycline biosynthesis	6453	8485	4027
Replication and repair	Base excision repair	7139	8396	5891
Membrane transport	Bacterial secretion system	6951	8067	4653
Replication and repair	Nucleotide excision repair	6495	7899	5712
Metabolism of terpenoids and polyketides	Biosynthesis of ansamycins	5919	7053	4167

Metabolism of cofactors and vitamins	Thiamine metabolism	5362	6441	4988
Amino acid metabolism	Valine leucine and isoleucine biosynthesis	4419	5259	3001
Translation	RNA transport	4336	5108	3495
Xenobiotics biodegradation and metabolism	Nitrotoluene degradation	3906	4869	2538
Folding, sorting and degradation	Sulfur relay system	3452	4327	2402
Metabolism of terpenoids and polyketides	Biosynthesis of type II polyketide backbone	3063	4161	1762
Cell motility	Flagellar assembly	3299	3893	1976
Membrane transport	ABC transporters	3032	3516	1991
Lipid metabolism	Synthesis and degradation of ketone bodies	2760	3436	1936
Transcription	RNA polymerase	2715	3342	2544
Folding, sorting and degradation	Protein export	2455	3200	1769
Immune diseases	Primary immunodeficiency	2366	3056	1393
Metabolism of terpenoids and polyketides	Biosynthesis of siderophore group nonribosomal peptides	1913	2646	1491
Infectious diseases : Bacterial	Pathogenic Escherichia coli infection	1168	1508	790
Cell motility	chemotaxis	1282	1473	632
Signaling molecules and interaction	Cell adhesion molecules CAMs	840	1069	391
Signaling molecules and interaction	ECM receptor interaction	736	966	575

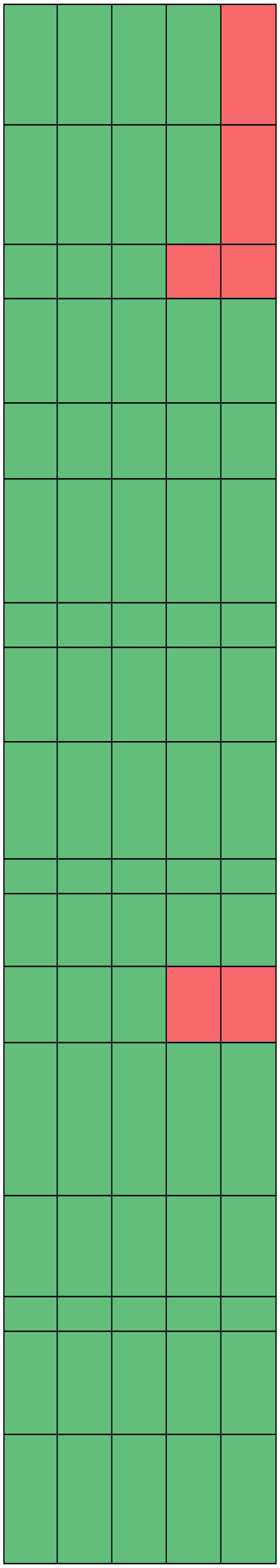

Biosynthesis of other secondary metabolites	beta Lactam resistance	431	543	241
Metabolism of cofactors and vitamins	Lipoic acid metabolism	318	482	113
Metabolism of terpenoids and polyketides	Nonribosomal peptide structures	194	199	161
Nucleotide metabolism	Purine metabolism	0	0	35803
Carbohydrate metabolism	Starch and sucrose metabolism	0	0	33378
Carbohydrate metabolism	Amino sugar and nucleotide sugar metabolism	0	0	25339
Amino acid metabolism	Arginine and proline metabolism	0	0	20503
Amino acid metabolism	Glycine serine and threonine metabolism	0	0	16017
Amino acid metabolism	Cysteine and methionine [illegible]	0	0	12343
Carbohydrate metabolism	Propanoate metabolism	0	0	9810
Metabolism of cofactors and vitamins	Porphyrin and chlorophyll metabolism	0	0	9084
Xenobiotics biodegradation and metabolism	Benzoate degradation	0	0	6971
Amino acid metabolism	Lysine biosynthesis	0	0	6716
Metabolism of cofactors and vitamins	Riboflavin metabolism	0	0	6063

Xenobiotics biodegradation and metabolism	Drug metabolism : other enzymes	0	0	5650
Amino acid metabolism	Valine leucine and isoleucine degradation	0	0	4867
Xenobiotics biodegradation and metabolism	Xylene degradation	0	0	4771
Metabolism of cofactors and vitamins	Biotin metabolism	0	0	4390
Xenobiotics biodegradation and metabolism	Dioxin degradation	0	0	4252
Biosynthesis of other secondary metabolites	Betalain biosynthesis	0	0	3469
Xenobiotics biodegradation and metabolism	Furfural degradation	0	0	663
Xenobiotics biodegradation and metabolism	Styrene degradation	0	0	594
Biosynthesis of other secondary metabolites	Puromycin biosynthesis	0	0	549
Metabolism of terpenoids and polyketides	Geraniol degradation	0	0	483
Chemical structure transformation maps	Biosynthesis of terpenoids and steroids	3	2	176
